# Supplementary material for: Rare earth stibolyl and bismolyl sandwich complexes
Source: Nat Commun. 2025 Feb 10;16:983. doi: 10.1038/s41467-024-55474-6 (PMC11811170; doi:10.1038/s41467-024-55474-6)
Supplement: Supplementary file 1 — Supplementary Information [file 41467_2024_55474_MOESM1_ESM.pdf]

Rare Earth Stibolyl and Bismolyl Sandwich Complexes

Noah Schwarz,[1] Florian Bruder,[2] Valentin Bayer,[3,4], Eufemio Moreno-Pineda,[5,6,7] Sebastian Gillhuber,[1] Xiaofei Sun,[1] Joris van Slageren,*[3] Florian Weigend,*[2] Peter W. Roesky*[1,8]

[1] Institute of Inorganic Chemistry, Karlsruhe Institute of Technology, Kaiserstraße 12, 76131 Karlsruhe, Germany

[2] Fachbereich Chemie, Philipps-Universität Marburg, Hans-Meerwein-Straße 4, 35043 Marburg, Germany

[3] Institute of Physical Chemistry, University of Stuttgart, Pfaffenwaldring 55, 70569 Stuttgart, Germany

[4] Institute of Inorganic Chemistry, University of Stuttgart, Pfaffenwaldring 55, 70569 Stuttgart, Germany

[5] Universidad de Panamá, Facultad de Ciencias Naturales, Exactas y Tecnología, Depto. de Química-Física, 0824, Panama

[6] Universidad de Panamá, Facultad de Ciencias Naturales, Exactas y Tecnología, Depto. Física, 0824, Panama

[7] Physikalisches Institut, Karlsruhe Institute of Technology, Kaiserstraße 12, 76131, Karlsruhe, Germany

[8] Institute of Nanotechnology, Karlsruhe Institute of Technology, Kaiserstraße 12, 76131, Karlsruhe, Germany

Corresponding author: roesky@kit.edu*, slageren@ipc.uni-stuttgart.de*, florian.weigend@chemie.uni-marburg.de*

Table of Contents

Supplementary Methods S3
Synthesis and characterization S3
General procedures S3
Synthesis of starting materials S4
Synthesis of ligands[7] S5
Synthesis of complexes[7] S7
NMR Spectra S12
IR spectra S18
X-ray crystallography S24
Supplementary Notes S32
Quantum Chemical Calculations S32
Magnetic Measurements S36
General Methodology S36
DC SQUID measurements S37
AC SQUID and DC relaxation measurements S43
ZFC and FC measurements S71
CASSCF Calculations S73
Supplementary References S78

Supplementary Methods

Synthesis and characterization

General procedures

All air- and moisture-sensitive manipulations were performed under dry N_2 or Ar atmosphere using standard Schlenk techniques or in an argon-filled MBraun glovebox, unless otherwise stated. Solvents (THF, *n*-pentane) were dried using an MBraun solvent purification system (SPS-800) and degassed. THF was additionally distilled under nitrogen from potassium benzophenone ketyl before storage over 4 Å molecular sieve. C_6D_6 and THF-d_8 were dried over Na-K alloy. All deuterated solvents were degassed by freeze-pump-thaw cycles. The starting materials [LnI(COT)(thf)$_2$] (Ln = Y, Er, Tb),[1] 1,4-bis(*tert*-butyl)-1,4-diiodo-2,3-dimethyl-1,3-butadiene,[2] 1,4-bis(trimethylsilyl)-1,4-diiodo-2,3-dimethyl-1,3-butadiene,[3] $PhSbCl_2$[4] and $PhBiCl_2$[5] were prepared according to literature known procedures.

Please note that the THF content in [LnI(COT)(thf)$_2$] (Ln = Y, Er, Tb) can vary depending on the drying duration and temperature as previously reported.[6] In our case, the used compounds typically retain two coordinating THF molecules when dried at room temperature.

All other chemicals were obtained from commercial sources and used without further purification. NMR spectra were recorded on Bruker spectrometers (Avance III 300 MHz, Avance Neo 400 MHz or Avance III 400 MHz). Chemical shifts are referenced using signals of the residual protio solvent (^{1}H) or the solvent (^{13}C) and are reported relative to tetramethylsilane (^{1}H, ^{13}C, ^{29}Si). All NMR spectra were measured at 298 K, unless otherwise specified. The multiplicity of the signals is indicated as s = singlet, d = doublet, t = triplet, m = multiplet and br = broad. Assignments were determined based on unambiguous chemical shifts, coupling patterns and ^{13}C-DEPT experiments or 2D correlations (^{1}H^{1}H COSY, ^{1}H^{13}C HMQC, ^{1}H^{13}C HMBC). Infrared (IR) spectra were recorded in the region 3600–400 cm^{-1} on a Bruker Tensor 37 FTIR spectrometer equipped with a room temperature DLaTGS detector and a diamond attenuated total reflection (ATR) unit. Elemental analyses were carried out with an elementar Vario Micro Cube.

Synthesis of starting materials

Synthesis of [LnI(COT)(thf)$_2$][1]

40 mL of THF were added to a Schlenk flask containing 4.70 mmol (1.00 eq) of finely grated lanthanide metal (Y, Er, Tb), which is sealed with a J. Young valve. Following this, 2.35 mmol (0.50 eq) of iodine, 4.70 mmol (1.00 eq) of cyclooctatetraene, and one drop of mercury are added to the stirred suspension. The reaction mixture is then sonicated at 80 °C for 2 hours. Afterwards, it is stirred for two weeks in an oil bath at 80 °C until most of the lanthanide metal has disappeared. After filtering the suspension over a glass frit, colorless (Y, Tb) and pink (Er) crystals were obtained from saturated THF solutions and the products were used without further purification. Please note that the THF content in [LnI(COT)(thf)$_2$] (Ln = Y, Er, Tb) can vary depending on the drying duration and temperature as previously reported.[6] In our case, the used compounds typically retain two coordinating THF molecules when dried at room temperature.

Synthesis of 1,4-bis(tert-butyl)-1,4-diiodo-2,3-dimethyl-1,3-butadiene[2]

Solid iodine (18.7 g, 73.8 mmol, 2.00 eq.) was added in small portions to a solution of 1,1-Bis(η^5-cyclopentadienyl)-2,5-bis(*tert*-butyl)-3,4-dimethyl-1-zirconacyclopentadiene (15.3 g, 36.9 mmol, 1.00 eq.) in 250 mL THF at 0 °C. After stirring the reaction mixture for two days, the solvent was evaporated, the resulting residue extracted with *n*-pentane and washed with a saturated $Na_2S_2O_3$ solution, water and brine (50 mL each). After drying the organic phase over $MgSO_4$ and evaporation of the solvent, the product was obtained as an off-white, microcrystalline solid.

Yield: 13.6 g, 36.9 mmol (83%)

The NMR data was consistent with the reported values in the literature.[2]

Synthesis of 1,4-bis(trimethylsilyl)-1,4-diiodo-2,3-dimethyl-1,3-butadiene[3]

Solid iodine (9.68 g, 38.1 mmol, 2.00 eq.) was added in small portions to a solution of 1,1-Bis(η^5-cyclopentadienyl)-2,5-bis(trimethylsilyl)-3,4-dimethyl-1-zirconacyclopentadiene (8.50 g, 19.1 mmol, 1.00 eq.) in 250 mL THF at 0 °C. After stirring the reaction mixture for two days, the solvent was evaporated, the resulting residue extracted with *n*-pentane and washed with a saturated $Na_2S_2O_3$ solution, water and brine (50 mL each). After drying the organic phase over $MgSO_4$ and evaporation of the solvent, the product was obtained as a yellow, microcrystalline solid.

Yield: 7.80 g, 16.3 mmol (86%)

The NMR data was consistent with the reported values in the literature.[3]

Synthesis of $PhSbCl_2$[4]

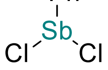

$SbPh_3$ (11.6 g, 50.9 mmol, 1.00 eq) was added to $SbCl_3$ and the two solids were stirred together forming a yellow oil, which was subsequently recrystallized from chloroform to give the desired product as a white solid.

Yield: 17.6 g, 65.2 mmol (85%)

The NMR data was consistent with the reported values in the literature.[4]

Synthesis of $PhBiCl_2$[5]

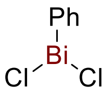

A solution of $BiPh_3$ (5.00 g, 11.4 mmol, 1.00 eq.) in 50 mL Et_2O was added to a suspension of $BiCl_3$ (7.16 g, 22.7 mmol, 2.00 eq.) in 50 mL Et_2O. The mixture was then stirred for 12 h and afterwards filtered through a glass frit. The remaining solid was then washed with Et_2O and dried under vacuum to give $PhBiCl_2$ as an off-white solid.

Yield: 11.1 g, 31.1 mmol (91%)

The NMR data was consistent with the reported values in the literature.[5]

Synthesis of ligands[7]

Synthesis of [K(Dtsb)] (1-Sb)

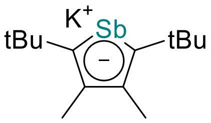

To a solution of 1,4-bis(*tert*-butyl)-1,4-diiodo-2,3-dimethyl-1,3-butadiene (5.14 g, 11.5 mmol, 1.00 eq) in 50 mL of Et_2O, *n*-BuLi (9.22mL , 23.0 mmol, 2.00 eq) was added at -78°C. After stirring for 1 h, $PhSbCl_2$ (3.11 g, 11.5 mmol, 1.00 eq) was added and the solution turned yellow with a white precipitate. After stirring for 12 h, the solvent was removed under reduced pressure and the yellow residue was redissolved in *n*-pentane. After filtering over a glass frit, the solvent was removed under reduced pressure again and 50 mL of THF was added to the remaining yellow solid. Then, an excess of potassium metal (1.80 g, 46.1 mmol, 4.00 eq) was added to the flask and the solution was stirred for three days at room temperature. After filtering, a green solution was obtained, and removal of the solvent gave **K(Dtsb)** as a green solid.

Yield: 3.10 g, 8.78 mmol (76%)

^{1}H NMR (THF-d_8, 400 MHz): δ [ppm] = 2.09 (s, 6 H, CH_3), 1.39 (s, 18 H, $C(CH_3)_3$).

^{13}C{^{1}H} NMR (THF-d_8, 100 MHz): δ [ppm] = 173.3 ($C^q(C(CH_3)_3)$), 132.8 ($C^q(CH_3)$), 39.3 ($C^q(CH_3)_3$), 34.2 ($C^q(CH_3)_3$), 21.1 (CH_3)).

IR (ATR): ($\tilde{\nu}$) [cm^{-1}] = 2950 (w), 2904 (w), 2863 (w), 2166 (vw), 1981 (vw), 1588 (w), 1461 (w), 1387 (vw), 1355 (w), 1311 (w), 1279 (vw), 1264 (vw), 1220 (w), 1079 (vw), 1053 (w), 1010 (vw), 982 (vw), 950 (vw), 898 (vw), 844 (vw), 763 (vw), 707 (vw), 578 (vw), 560 (vw), 475 (vw).

EI-MS m/z (%) = 313.0 $[M - K]^+$ (76).

Synthesis of [K(Dssb)] (2-Sb)

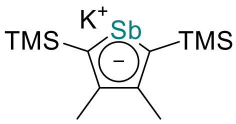

To a solution of 1,4-bis(trimethylsilyl)-1,4-diiodo-2,3-dimethyl-1,3-butadiene (2.66 g, 5.56 mmol, 1.00 eq) in 50 mL of Et_2O, *n*-BuLi (4.45mL , 11.1 mmol, 2.00 eq) was added at -78°C. After stirring for 1 h, $PhSbCl_2$ (1.50 g, 5.56 mmol, 1.00 eq) was added and the solution turned yellow with a white precipitate. After stirring for 12 h, the solvent was removed under reduced pressure and the yellow residue was redissolved in *n*-pentane. After filtering over a glass frit, the solvent was removed under reduced pressure again and 50 mL of THF was added to the remaining yellow solid. Then, an excess of potassium metal (0.87 g, 22.2 mmol, 4.00 eq) was added to the flask and the solution was stirred for three days at room temperature. After filtering, a red-brown solution was obtained, and removal of the solvent gave **K(Dssb)** as a light-brown solid.

Yield: 0.71 g, 1.84 mmol (33%)

^{1}H NMR (THF-d$_8$, 400 MHz): *δ* [ppm] = 2.22 (s, 6 H, C*H*$_3$), 0.22 (s, 18 H, Si(C*H*$_3$)$_3$).

^{13}C{^{1}H} NMR (THF-d$_8$, 100 MHz): *δ* [ppm] = 161.4 (*C*^{q}(Si(CH$_3$)$_3$)), 145.2 (*C*^{q}(CH$_3$)), 24.5 (*C*H$_3$), 3.3 (Si(*C*H$_3$)$_3$).

IR (ATR): ($\tilde{\nu}$) [cm^{-1}] = 2949 (w), 2891 (vw), 2858 (vw), 2166 (vw), 1594 (vw), 1410 (vw), 1325 (vw), 1307 (vw), 1244 (w), 1110 (w), 1051 (w), 1021 (w), 992 (vw), 898 (vw), 825 (m), 742 (w), 679 (w), 629 (w), 487 (w), 448 (w).

EA Calcd (%) for [$C_{12}H_{24}SbSi_2K$] (385.35 g mol^{-1}): C 37.40, H 6.28; found: C 37.37, H 6.18.

Synthesis of [K(Dtbi)] (1-Bi)

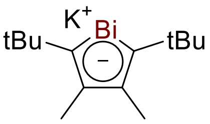

To a solution of 1,4-bis(*tert*-butyl)-1,4-diiodo-2,3-dimethyl-1,3-butadiene (2.69 g, 6.02 mmol, 1.00 eq) in 50 mL of Et$_2$O, *n*-BuLi (4.82 mL, 12.1 mmol, 2.00 eq) was added at -78°C. After stirring for 1 h, PhBiCl$_2$ (2.15 g, 6.02 mmol, 1.00 eq) was added and the solution turned brown with a white precipitate. After stirring for 12 h, the solvent was removed under reduced pressure and the yellow residue was redissolved in *n*-pentane. After filtering over a glass frit, the solvent was removed under reduced pressure again and 50 mL of THF was added to the remaining brown-orange solid. Then, an excess of potassium metal (0.94 g, 24.1 mmol, 4.00 eq) was added to the flask and the solution was stirred for three days at room temperature. After filtering, a red-brown solution was obtained, and removal of the solvent gave **K(Dtbi)** as a brown solid.

Yield: 1.57 g, 3.56 mmol (59%)

^{1}H NMR (THF-d$_8$, 400 MHz): *δ* [ppm] = 1.99 (s, 6 H, C*H*$_3$), 1.32 (s, 18 H, C(C*H*$_3$)$_3$).

^{13}C{^{1}H} NMR (THF-d$_8$, 100 MHz): *δ* [ppm] = 186.8 (*C*^{q}(C(CH$_3$)$_3$)), 146.0 (*C*^{q}(CH$_3$)), 44.4 (*C*^{q}(CH$_3$)$_3$), 34.1 (C^q(*C*H$_3$)$_3$), 26.4 (*C*H$_3$)).

IR (ATR): ($\tilde{\nu}$) [cm^{-1}] =2947 (w), 2901 (w), 2861 (w), 1574 (w), 1459 (w), 1391 (w), 1353 (w), 1262 (vw), 1222 (w), 1189 (vw), 1052 (w), 1010 (vw), 934 (vw), 898 (w), 843 (vw), 775 (vw), 723 (vw), 643 (vw), 564 (w).

EI-MS m/z (%) = 401.2 [M - K]$^+$ (94).

Synthesis of complexes[7]

General procedure for synthesis of [Ln(COT)(stibole)] complexes

At room temperature, 10 mL of *n*-pentane was condensed onto a mixture of [LnI(COT)(thf)$_2$] (Ln = Y, Er, Tb) and the potassium stibolide (K(dtsb), K(dssb)). After stirring for 2 d, the product was filtered and concentrated. Crystals could be obtained from concentrated *n*-pentane or toluene solutions at room temperature.

Synthesis of [Y(COT)(dtsb)] (3-Y)

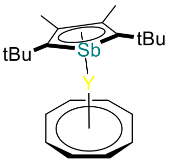

According to the general procedure, 100 mg (0.22 mmol, 1.00 eq.) of [YI(COT)(thf)$_2$] and 76.1 mg (0.22 mmol, 1.00 eq.) of K(dtsb) were reacted in *n*-pentane. Crystals suitable for XRD analysis were grown from a saturated *n*-pentane solution at room temperature.

Crystalline yield: 54.0 mg, 0.11 mmol (49%)

^{1}H NMR (C_6D_6, 400 MHz): δ [ppm] = 6.35 (s, 8 H, C_8H_8), 1.94 (s, 6 H, CH_3), 1.30 (s, 18 H, $C(CH_3)_3$).

^{13}C{^{1}H} NMR (C_6D_6, 100 MHz): δ [ppm] = 187.5 (d, $^1J_{CY}$ = 2.2 Hz, $C^q(C(CH_3)_3)$), 139.8 ($C^q(CH_3)$), 94.5 (d, $^1J_{CY}$ = 3.0 Hz, C_8H_8), 39.5 ($C^q(CH_3)_3$), 34.4 ($C^q(CH_3)_3$), 20.8 (CH_3)).

IR (ATR): ($\tilde{\nu}$) [cm^{-1}] = 3039 (vw), 2979 (vw), 2952 (m), 2861 (w), 2165 (vw), 1850 (vw), 1744 (vw), 1601 (vw), 1459 (w), 1377 (w), 1356 (m), 1310 (w), 1228 (w), 1192 (w), 1117 (vw), 1026 (vw), 1006 (w), 943 (vw), 896 (m), 767 (w), 749 (w), 703 (s), 579 (w), 508 (vw).

EA Calcd (%) for [$C_{22}H_{32}SbY$] (507.16 g mol^{-1}): C 52.10, H 6.36; found C 53.12, H 6.16.

No better elemental analysis data could be obtained despite repeated attempts. The bulk purity of [**Y(COT)(Dtsb)**] can be assessed from its NMR spectra (see Fig. S7-S8).

Synthesis of [Er(COT)(dtsb)] (3-Er)

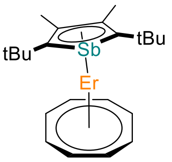

According to the general procedure, 100 mg (0.18 mmol, 1.00 eq.) of [ErI(COT)(thf)$_2$] and 65.1 mg (0.18 mmol, 1.00 eq.) of K(dtsb) were reacted in *n*-pentane. Crystals suitable for XRD analysis were grown from a saturated *n*-pentane solution at room temperature.

Crystalline yield: 73.4 mg, 0.13 mmol (68%)

IR (ATR): ($\tilde{\nu}$) [cm^{-1}] = 2952 (m), 2900 (w), 2862 (w), 2166 (vw), 1850 (vw), 1746 (vw), 1604 (vw), 1463 (w), 1375 (w), 1357 (w), 1310 (vw), 1228 (w), 1195 (vw), 1116 (vw), 1007 (vw), 944 (vw), 895 (w), 771 (w), 749 (w), 706 (m), 643 (w), 577 (w), 507 (vw).

EA Calcd (%) for [$C_{22}H_{32}SbEr$] (585.52 g mol^{-1}): C 45.13, H 5.51; found C 45.89, H 5.93.

Synthesis of [Tb(COT)(dtsb)] (3-Tb)

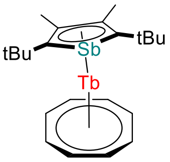

According to the general procedure, 125 mg (0.23 mmol, 1.00 eq.) of [TbI(COT)(thf)$_2$] and 82.7 mg (0.23 mmol, 1.00 eq.) of K(dtsb) were reacted in *n*-pentane. Crystals suitable for XRD analysis were grown from a saturated *n*-pentane solution at room temperature.

Crystalline yield: 35.3 mg, 0.06 mmol (26%)

IR (ATR): ($\tilde{\nu}$) [cm^{-1}] = 3036 (vw), 2950 (m), 2901 (m), 2862 (w), 1852 (vw), 1749 (vw), 1605 (vw), 1463 (w), 1389 (vw), 1376 (w), 1357 (w), 1309 (vw), 1229 (w), 1193 (vw), 1115 (vw), 1024 (vw), 1005 (vw), 944 (vw), 895 (w), 770 (vw), 747 (vw), 705 (m), 675 (w), 578 (vw).

EA Calcd (%) for [$C_{22}H_{32}SbTb$] (577.18 g mol^{-1}): C 45.78, H 5.59; found C 45.35, H 5.57.

Synthesis of [Y(COT)(dssb)] (4-Y)

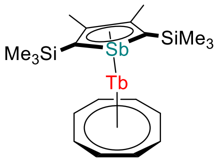

According to the general procedure, 100 mg (0.22 mmol, 1.00 eq.) of [YI(COT)(thf)$_2$] and 83.0 mg (0.22 mmol, 1.00 eq.) of K(dssb) were reacted in *n*-pentane. Crystals suitable for XRD analysis were grown from a saturated *n*-pentane solution at room temperature.

Crystalline yield: 61.2 mg, 0.11 mmol (53%)

^{1}H NMR (C_6D_6, 400 MHz): δ [ppm] = 6.31 (s, 8 H, C_8H_8), 2.01 (s, 6 H, CH_3), 0.32 (s, 18 H, Si(CH_3)$_3$).

^{13}C{^{1}H} NMR (C_6D_6, 100 MHz): δ [ppm] = 174.5 (d, $^1J_{CY}$ = 2.5 Hz, C^q(Si(CH$_3$)$_3$)), 151.0 (C^q(CH$_3$)), 94.3 (d, $^1J_{CY}$ = 3.2 Hz, C_8H$_8$), 23.8 (CH$_3$), 2.96 (Si(CH$_3$)$_3$).

IR (ATR): ($\tilde{\nu}$) [cm^{-1}] = 3037 (vw), 2950 (w), 2893 (vw), 1848 (vw), 1745 (vw), 1600 (vw), 1396 (w), 1375 (vw), 1309 (vw), 1242 (m), 1117 (w), 1022 (w), 894 (w), 825 (s), 752 (m), 701 (s), 631 (m), 497 (w), 444 (w).

EA Calcd (%) for [$C_{20}H_{32}SbSi_2Y$] (539.31 g mol^{-1}): C 44.54, H 5.98; found C 43.87, H 5.68.

No better elemental analysis data could be obtained despite repeated attempts. The bulk purity of [**Y(COT)(Dtsb)**] can be assessed from its NMR spectra (see Fig. S9-S10).

Synthesis of [Er(COT)(dssb)] (4-Er)

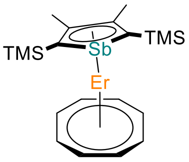

According to the general procedure, 100 mg of [ErI(COT)(thf)$_2$] (0.18 mmol, 1.00 eq.) and 71.0 mg (0.18 mmol, 1.00 eq.) of K(dssb) were reacted in *n*-pentane. Crystals suitable for XRD analysis were grown from a saturated *n*-pentane solution at room temperature.

Crystalline yield: 43.4 mg, 0.07 mmol (38%)

IR (ATR): ($\tilde{\nu}$) [cm^{-1}] = 3041 (vw), 2991 (vw), 2947 (w), 2891 (vw), 2166 (vw), 1848 (vw), 1744 (vw), 1599 (vw), 1441 (vw), 1399 (w), 1377 (vw), 1308 (vw), 1244 (m), 1120 (w), 1020 (w), 911 (w), 894 (w), 826 (s), 747 (m), 702 (s), 629 (m), 567 (vw), 498 (w), 444 (m).

EA Calcd (%) for [$C_{20}H_{32}SbSi_2Er$] (617.66 g mol^{-1}): C 38.89, H 5.22 found C 39.46, H 4.97.

Synthesis of [Tb(COT)(dssb)] (4-Tb)

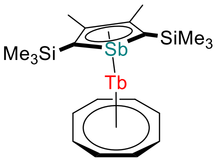

According to the general procedure, 43.0 mg (0.08 mmol, 1.00 eq.) of [TbI(COT)(thf)$_2$] and 31.0 mg (0.08 mmol, 1.00 eq.) of K(dssb) were reacted in *n*-pentane. Crystals suitable for XRD analysis were grown from a saturated *n*-pentane solution at room temperature.

Crystalline yield: 8.05 mg, 0.01 mmol (16%)

IR (ATR): ($\tilde{\nu}$) [cm^{-1}] = 3036 (vw), 3007 (vw), 2949 (m), 2893 (w), 1851 (vw), 1750 (vw), 1606 (vw), 1513 (vw), 1431 (vw), 1398 (w), 1375 (vw), 1308 (vw), 1243 (m), 1116 (w), 1086 (vw), 1022 (w), 931 (vw), 910 (vw), 893 (vw), 828 (s), 773 (w), 752 (w), 702 (m), 684 (w), 629 (m), 495 (vw), 444 (vw).

EA Calcd (%) for [$C_{20}H_{32}SbSi_2Tb$] (609,33 g mol^{-1}): C 39.42, H 5.29; found C 39.79, H 5.15.

General procedure for synthesis of [Ln(COT)(bismole)] complexes

At room temperature, 10 mL of n-n-pentane was condensed onto a mixture of [LnI(COT)(thf)$_2$] (Ln = Y, Er, Tb, Er) and the potassium bismolide **1-Bi**. After stirring for 2 d, the product was filtered and concentrated. Crystals could be obtained from concentrated *n*-pentane solutions at room temperature.

Synthesis of [Y(COT)(dtbi)] (5-Y)

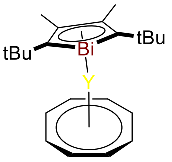

According to the general procedure, 100 mg (0.22 mmol, 1.00 eq.) of [YI(COT)(thf)$_2$] and 94.9 mg (0.22 mmol, 1.00 eq.) of K(dtbi) were reacted in *n*-pentane. Crystals suitable for XRD analysis were grown from a saturated *n*-pentane solution at room temperature.

Crystalline yield: 45.0 mg, 0.08 mmol (35%).

^{1}H NMR (C_6D_6, 400 MHz): δ [ppm] = 6.39 (s, 8 H, C_8H_8), 1.87 (s, 6 H, CH_3), 1.28 (s, 18 H, $C(CH_3)_3$).

^{13}C{^{1}H} NMR (C_6D_6, 100 MHz): δ [ppm] = 210.0 ($C^q(C(CH_3)_3)$, 152.9 ($C^q(CH_3)$), 94.6 (d, $^1J_{CY}$ = 2.8 Hz, C_8H_8), 44.8 ($C^q(CH_3)_3$), 34.7 ($C^q(CH_3)_3$), 27.3 (CH_3)).

IR (ATR): ($\tilde{\nu}$) [cm^{-1}] = 3037 (vw), 2981 (vw), 2951 (m), 2899 (w), 2860 (w), 2165 (vw), 1849 (vw), 1744 (vw), 1601 (vw), 1541 (vw), 1460 (w), 1419 (vw), 1377 (w), 1355 (m), 1310 (w), 1274 (vw), 1222 (w), 1199 (w), 1107 (vw), 1025 (vw), 1004 (w), 927 (vw), 894 (m), 836 (vw), 767 (w), 748 (w), 704 (s), 635 (w), 565 (m), 503 (vw), 417 (vw).

EA Calcd (%) for [$C_{22}H_{32}BiY$] (594.38 g mol^{-1}): C 44.46, H 5.43; found C 43.80, H 5.24.

No better elemental analysis data could be obtained despite repeated attempts. The bulk purity of [**Y(COT)(dtbi)**] can be assessed from its NMR spectra (see Fig. S11-S12).

Synthesis of [Tb(COT)(dtbi)] (5-Tb)

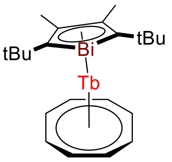

According to the general procedure, 160 mg (0.30 mmol, 1.00 eq.) of [TbI(COT)(thf)$_2$] and 131.9 mg (0.30 mmol, 1.00 eq.) of K(dtbi) were reacted in *n*-pentane. Crystals suitable for XRD analysis were grown from a saturated *n*-pentane solution at room temperature.

Crystalline yield: 76.3 mg, 0.11 mmol (38%)

IR (ATR): ($\tilde{\nu}$) [cm^{-1}] = 3036 (vw), 2950 (m), 2890 (w), 2860 (w), 1852 (vw), 1748 (vw), 1605 (vw), 1459 (w), 1420 (vw), 1376 (w), 1355 (m), 1309 (w), 1273 (vw), 1221 (w), 1199 (w), 1107 (vw), 1024 (vw), 1003 (w), 926 (vw), 894 (m), 841 (vw), 770 (w), 748 (w), 704 (s), 634 (w), 564 (w), 504 (vw), 417 (vw).

EA Calcd (%) for [$C_{22}H_{32}BiTb$] (664.40 g mol^{-1}): C 39.77, H 4.85; found C 39.12, H 4.49.

Synthesis of [Er(COT)(dtbi)] (5-Er)

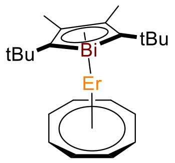

According to the general procedure, 100 mg (0.18 mmol, 1.00 eq.) of [ErI(COT)(thf)$_2$] and 81.2 mg (0.18 mmol, 1.00 eq.) of K(dtbi) were reacted in *n*-pentane. Crystals suitable for XRD analysis were grown from a saturated *n*-pentane solution at room temperature.

Crystalline yield: 68.0 mg, 0.10 mmol (55%)

IR (ATR): ($\tilde{\nu}$) [cm^{-1}] = 3039 (vw), 2950 (m), 2902 (w), 2861 (w), 2165 (vw), 1850 (vw), 1745 (vw), 1603 (vw), 1461 (w), 1388 (vw), 1375 (w), 1355 (m), 1310 (vw), 1273 (vw), 1221 (w), 1189 (w), 1107 (vw),1007 (vw), 916 (vw), 894 (m), 768 (w), 749 (w), 705 (s), 634 (vw), 564 (w), 474 (vw).

EA Calcd (%) for [$C_{22}H_{32}BiEr$] (672.74 g mol^{-1}): C 39.28, H 4.79; found C 39.19, H 4.58.

NMR Spectra

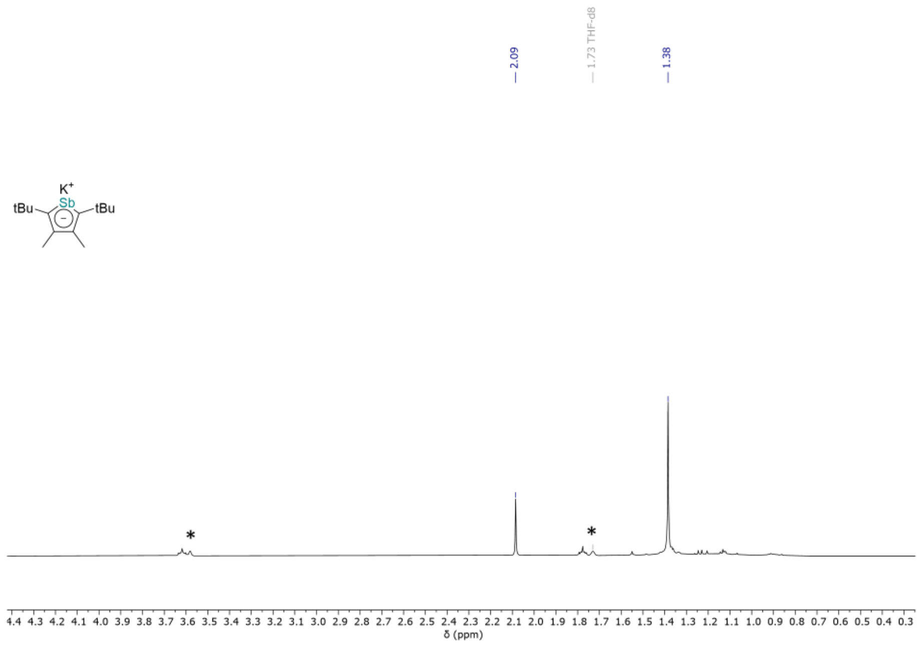

Figure S1: ^{1}H NMR spectrum (400 MHz, THF-d_8, 298 K) of **1-Sb [K(dtsb)]**. *: residual protio solvent signal.

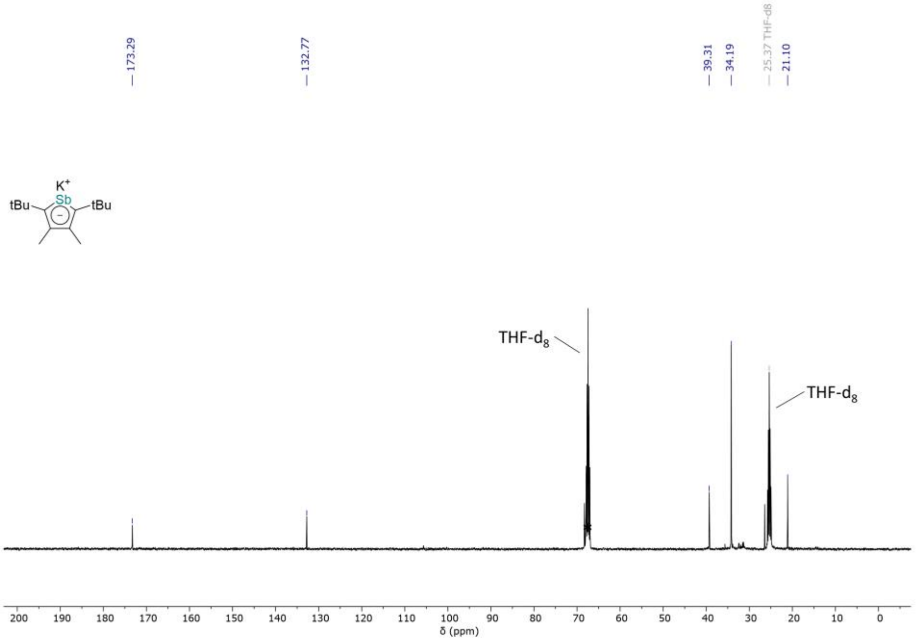

Figure S2: ^{13}C NMR spectrum (100 MHz, THF-d_8, 298 K) of **1-Sb** [**K(dtsb)**].

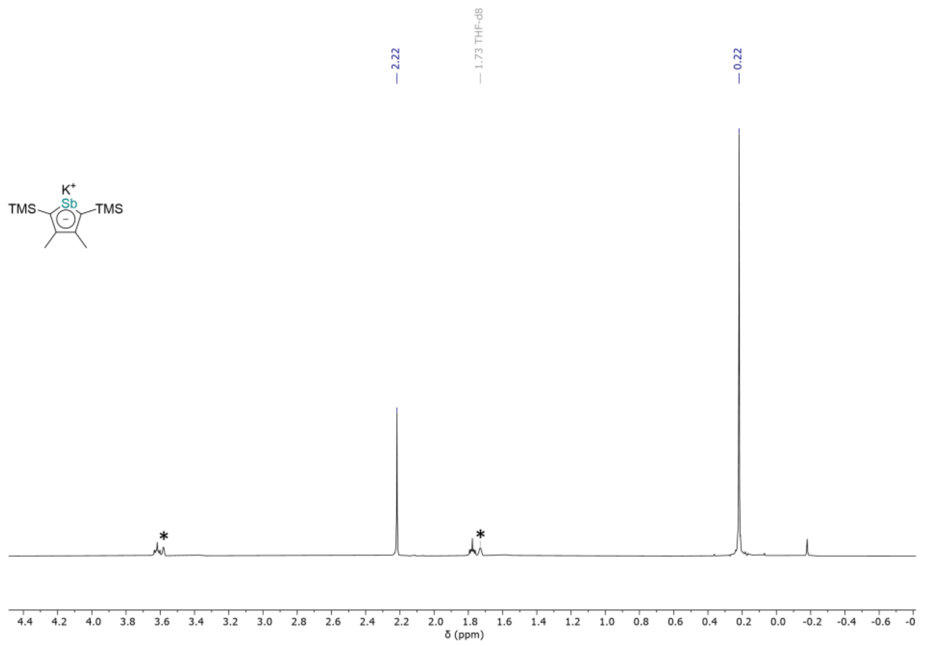

Figure S3: ^1H NMR spectrum (400 MHz, THF-d_8, 298 K) of **2-Sb** [**K(dssb)]**. *: residual protio solvent signal.

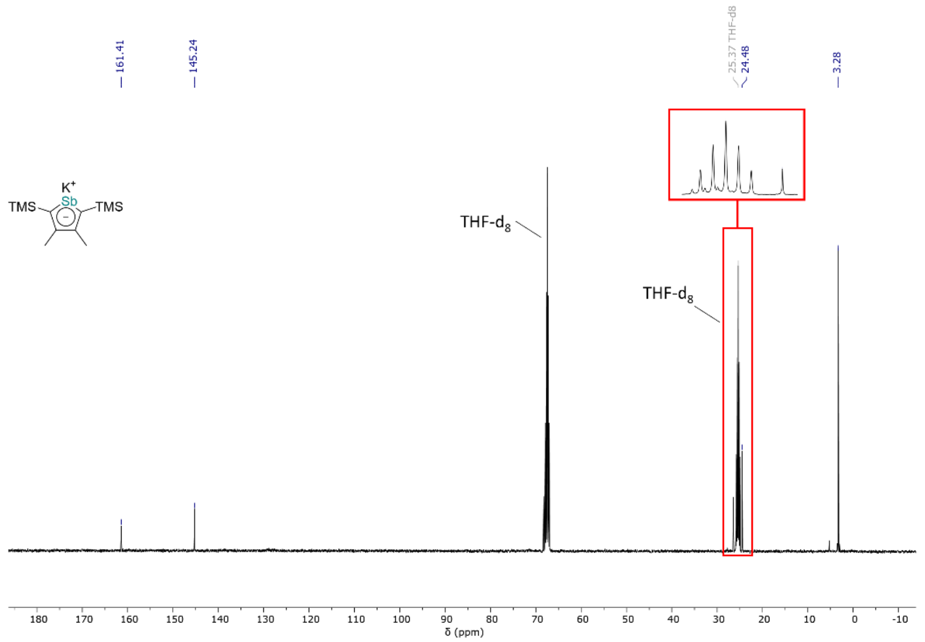

Figure S4: $^{13}C\{^1H\}$ NMR spectrum (100 MHz, THF-d_8, 298 K) of **2-Sb** [**K(dssb)]**.

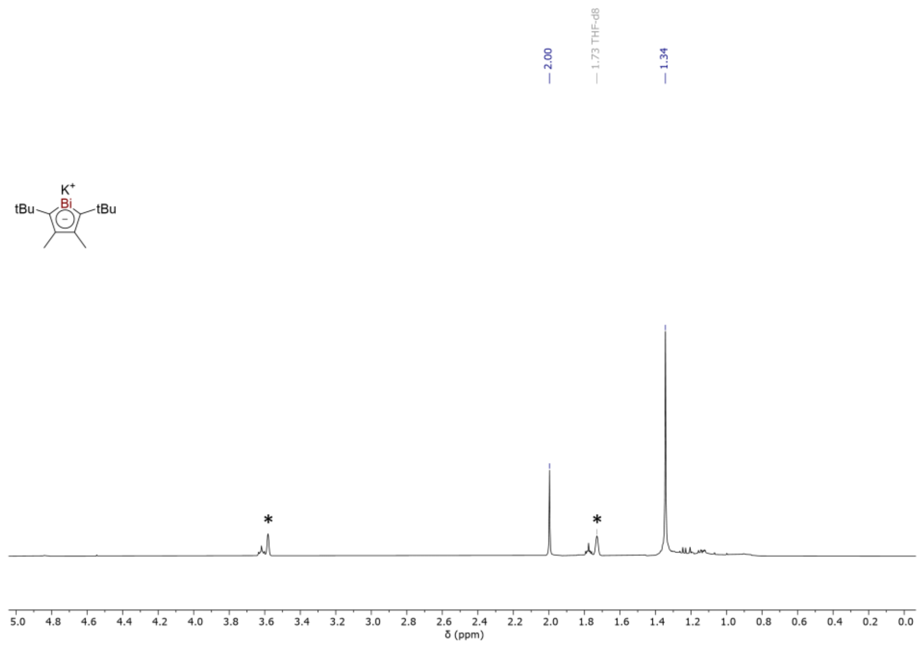

Figure S5: ^{1}H NMR spectrum (400 MHz, THF-d_8, 298 K) of **1-Bi [K(dtbi)]**. *: residual protio solvent signal.

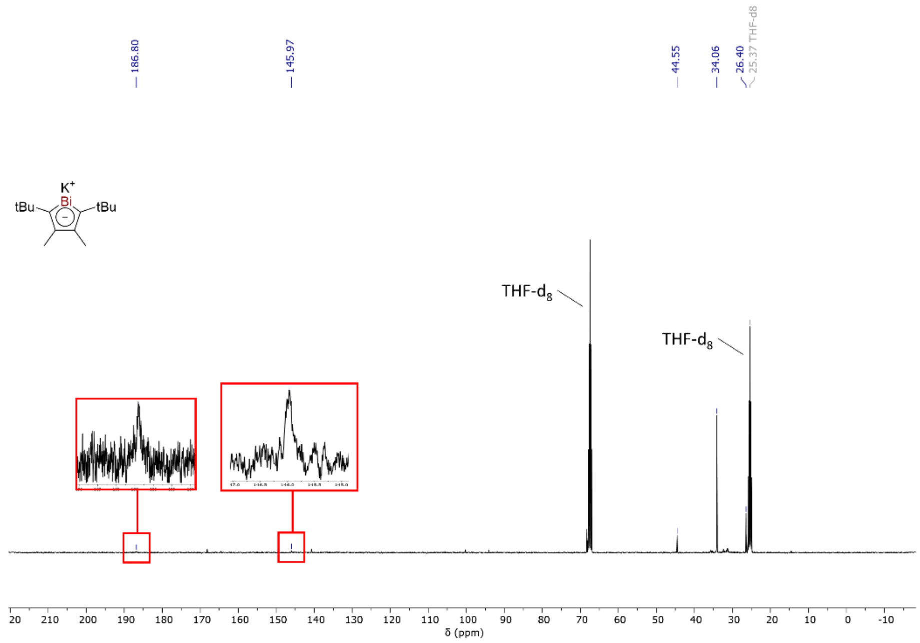

Figure S6: $^{13}C\{^{1}H\}$ NMR spectrum (100 MHz, THF-d_8, 298 K) of **1-Bi [K(dtbi)]**.

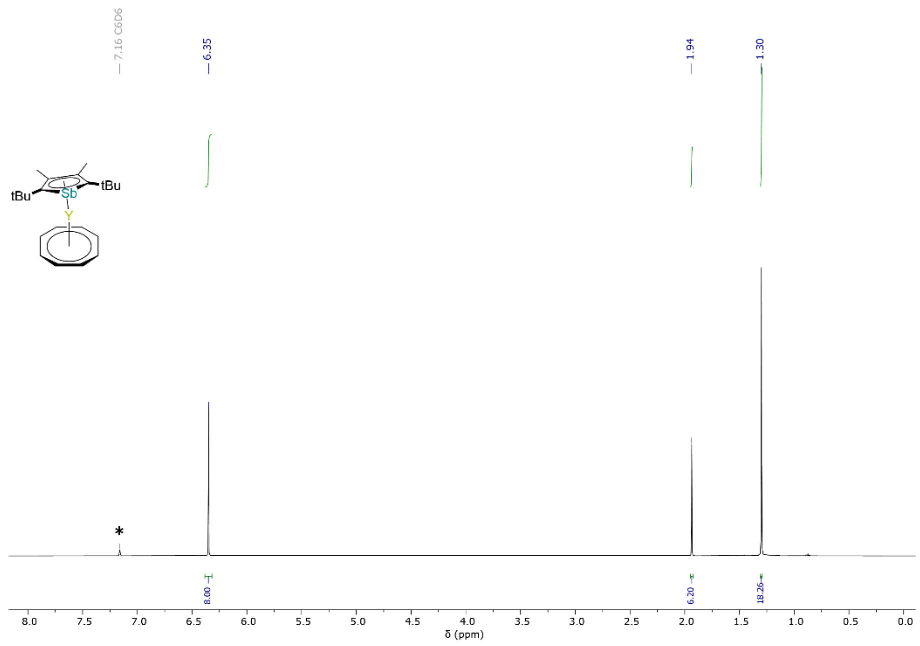

Figure S7: ^{1}H NMR spectrum (400 MHz, C_6D_6, 298 K) of **3-Y [Y(COT)(dtsb)]**. *: residual protio solvent signal.

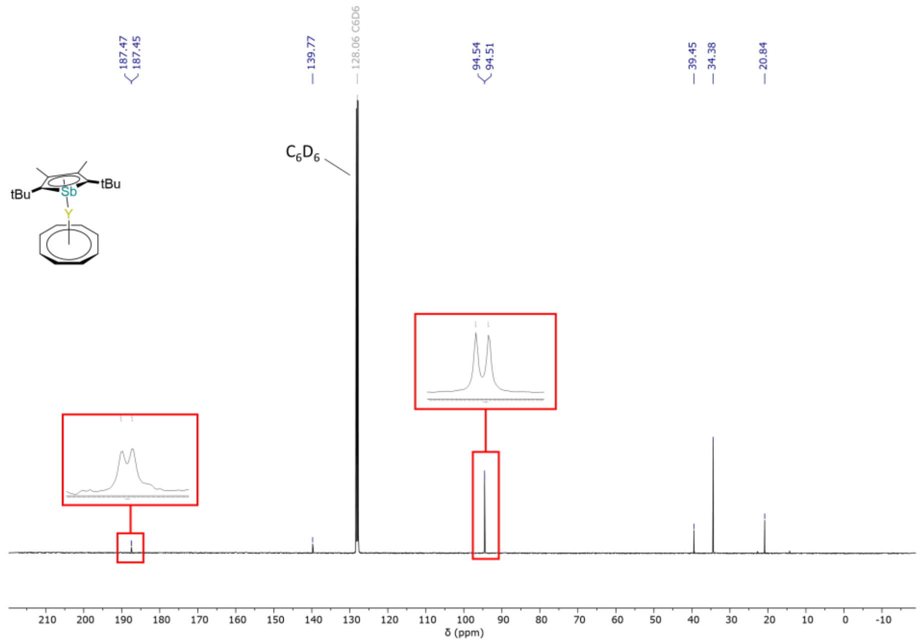

Figure S8: $^{13}C\{^1H\}$ NMR spectrum (100 MHz, C_6D_6, 298 K) of **3-Y [Y(COT)(dtsb)]**.

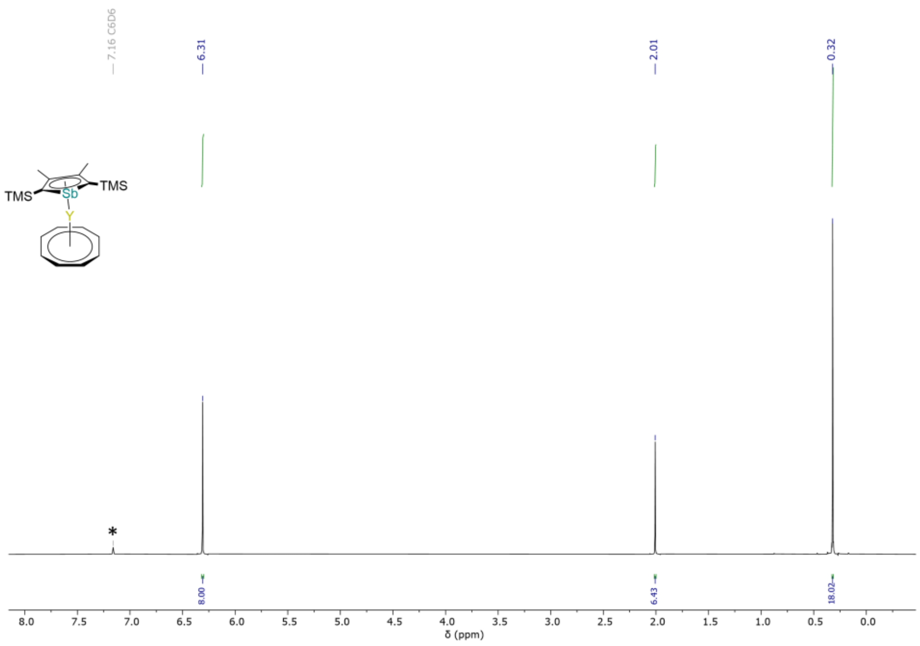

Figure S9: ^{1}H NMR spectrum (400 MHz, C_6D_6, 298 K) of **4-Y [Y(COT)(dssb)]**. *: residual protio solvent signal.

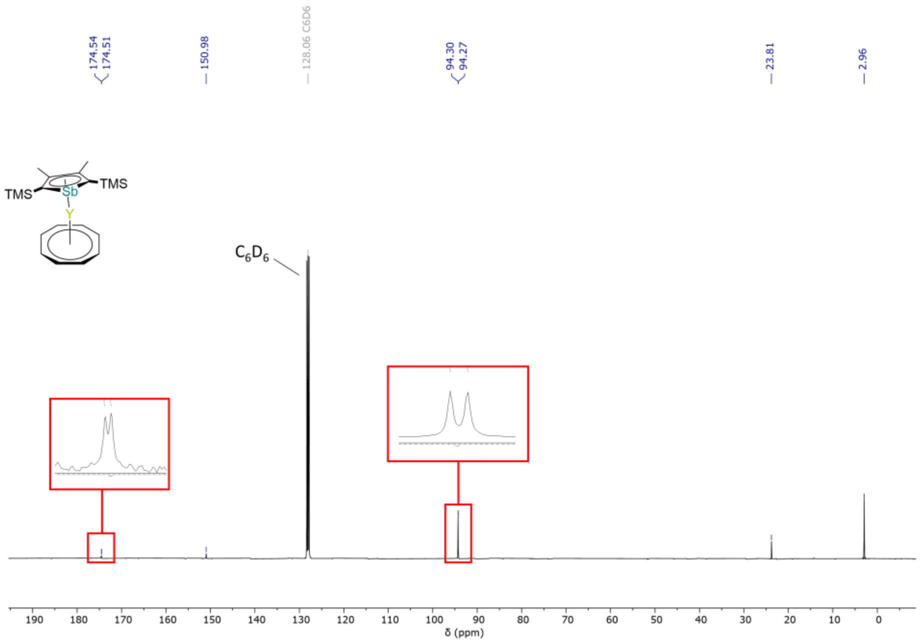

Figure S10: $^{13}C\{^{1}H\}$ NMR spectrum (100 MHz, C_6D_6, 298 K) of **4-Y [Y(COT)(dssb)]**.

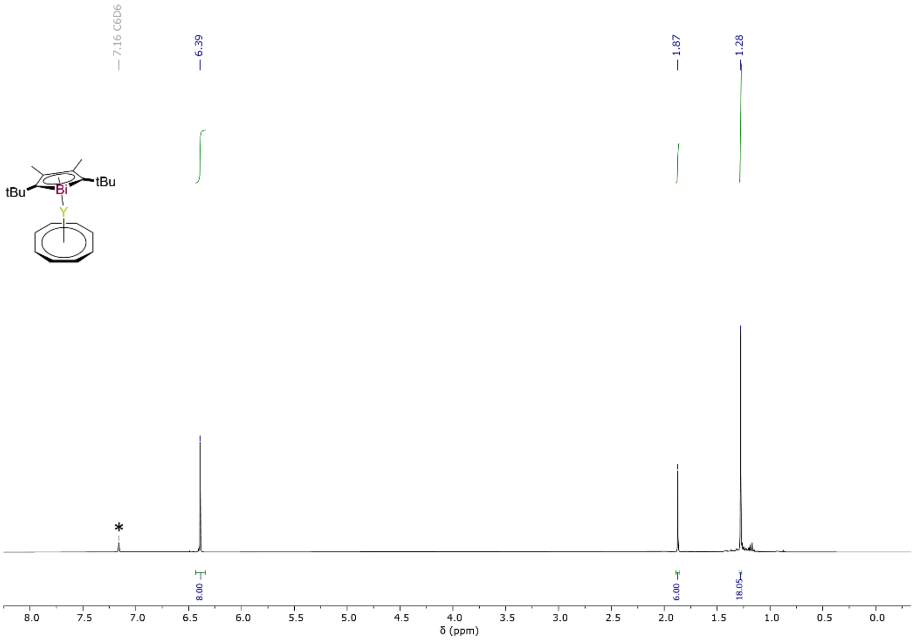

Figure S11: ^{1}H NMR spectrum (400 MHz, C_6D_6, 298 K) of **5-Y [Y(COT)(dtbi)]**. *: residual protio solvent signal.

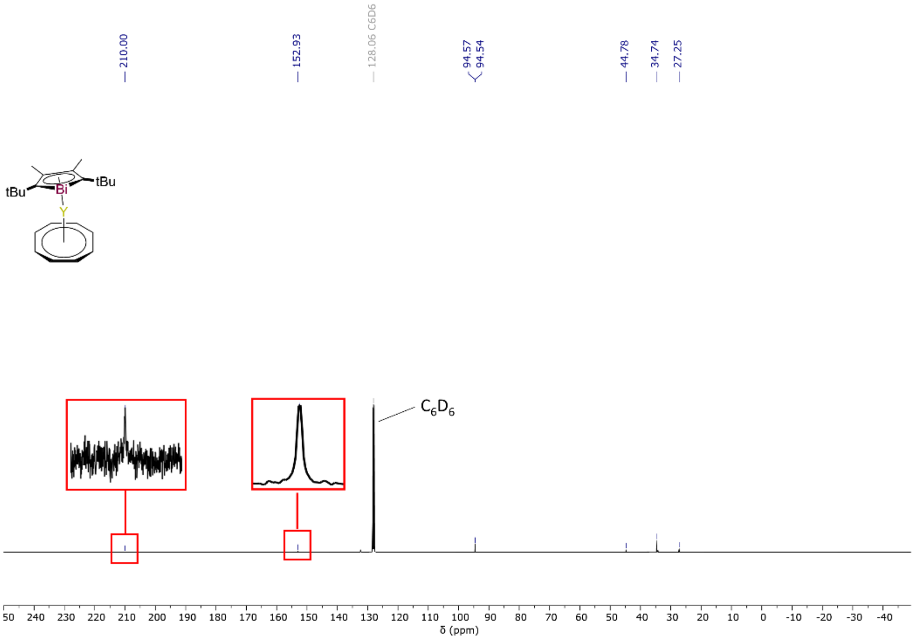

Figure S12: $^{13}C\{^{1}H\}$NMR spectrum (100 MHz, C_6D_6, 298 K) of **5-Y [Y(COT)(dtbi)]**.

IR spectra

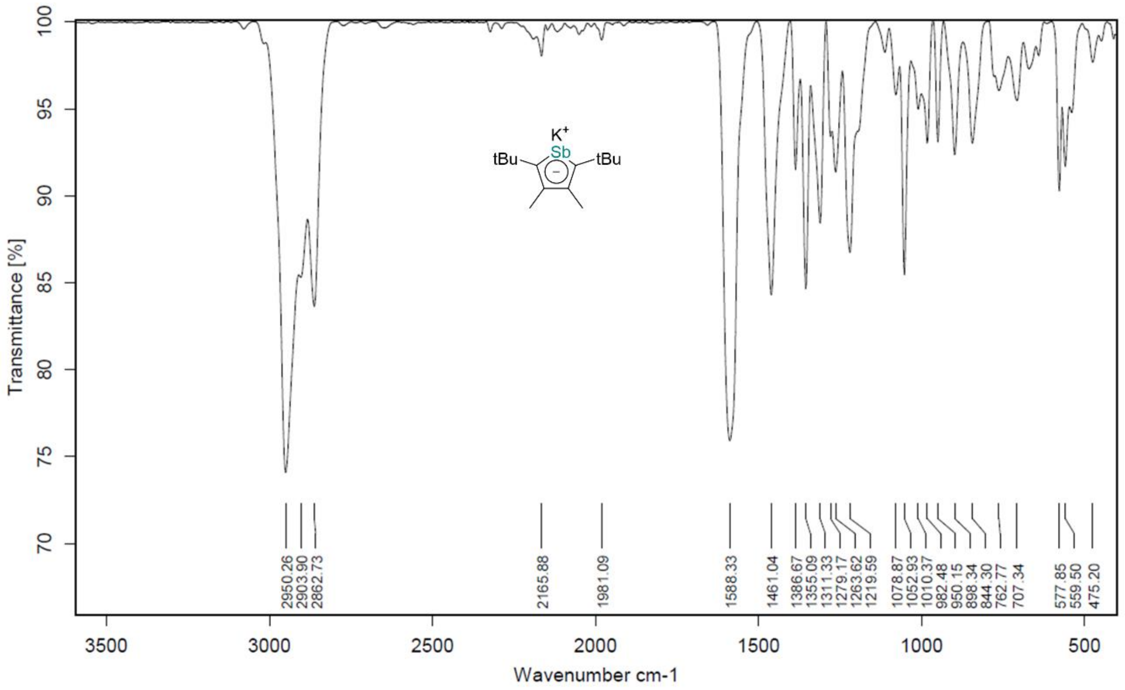

Figure S13: IR spectrum of **1-Sb [K(dtsb)].**

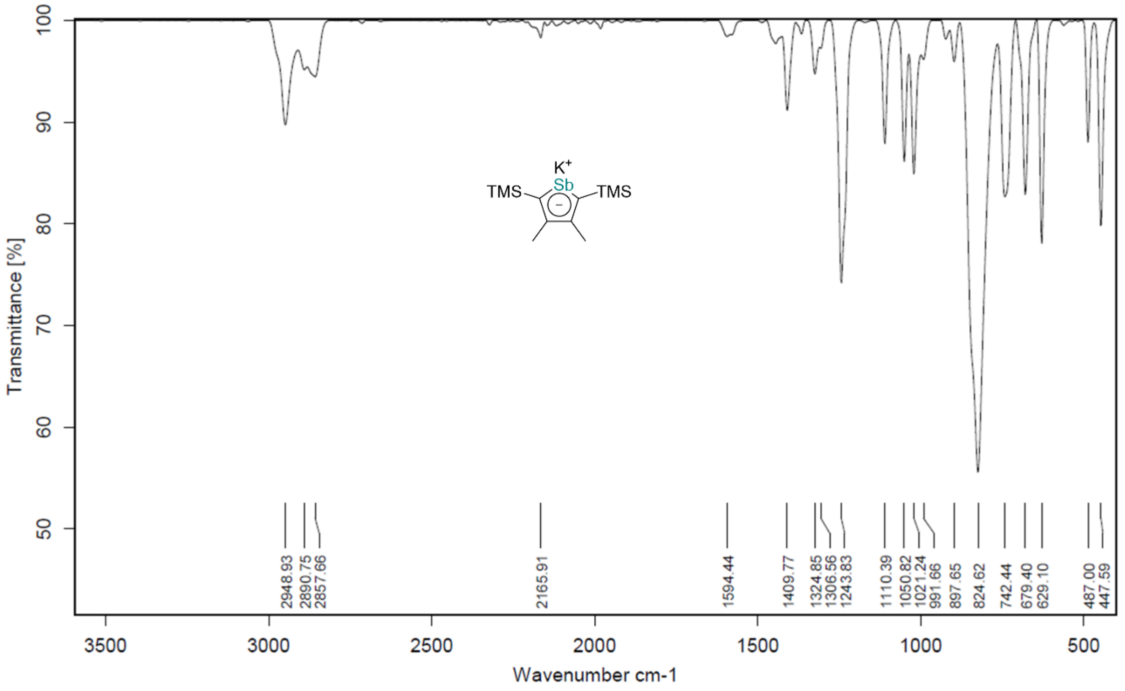

Figure S14: IR spectrum of **2-Sb [K(dssb)].**

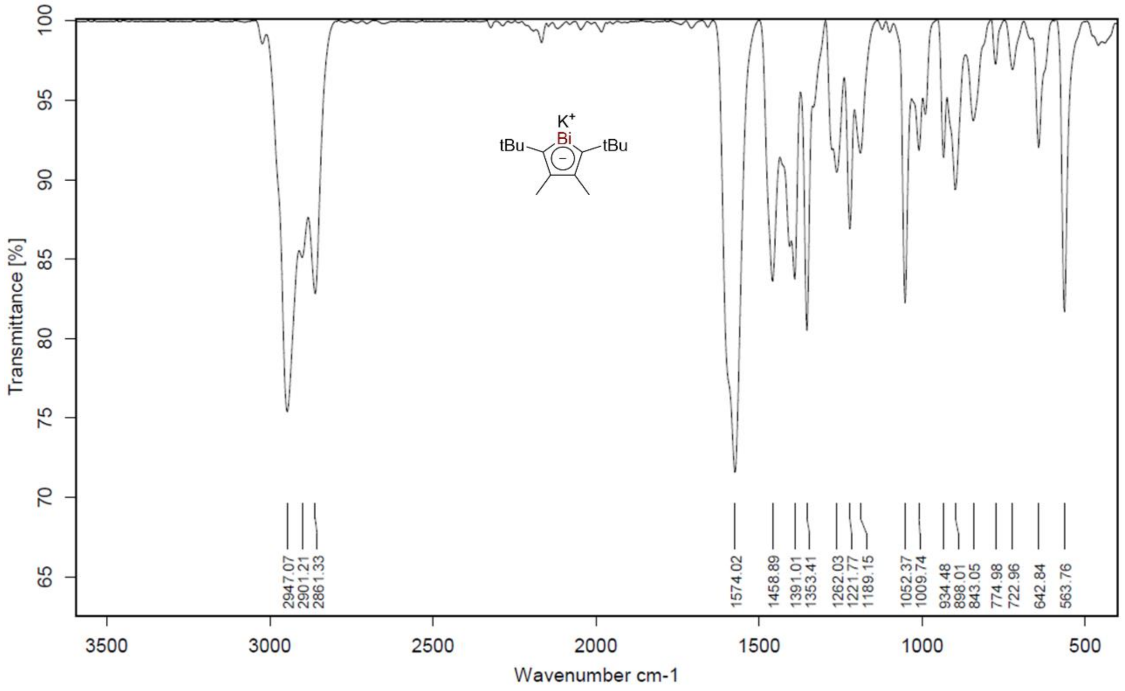

Figure S15: IR spectrum of **1-Bi [K(dtbi)].**

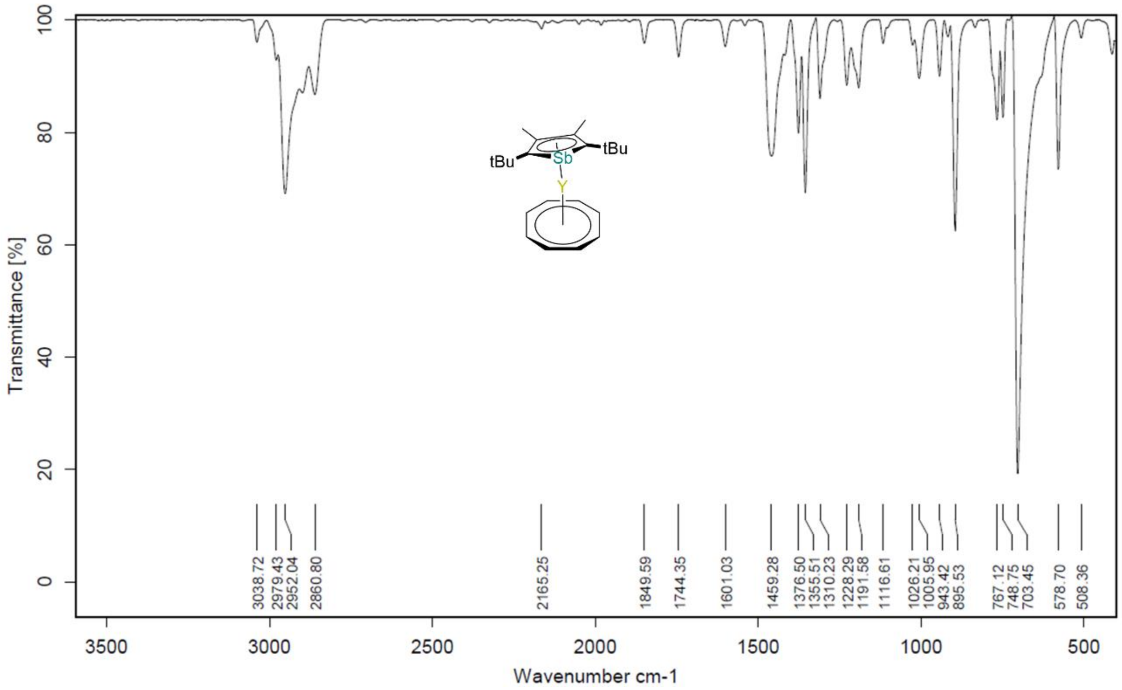

Figure S16: IR spectrum of **3-Y [Y(COT)(dtsb)].**

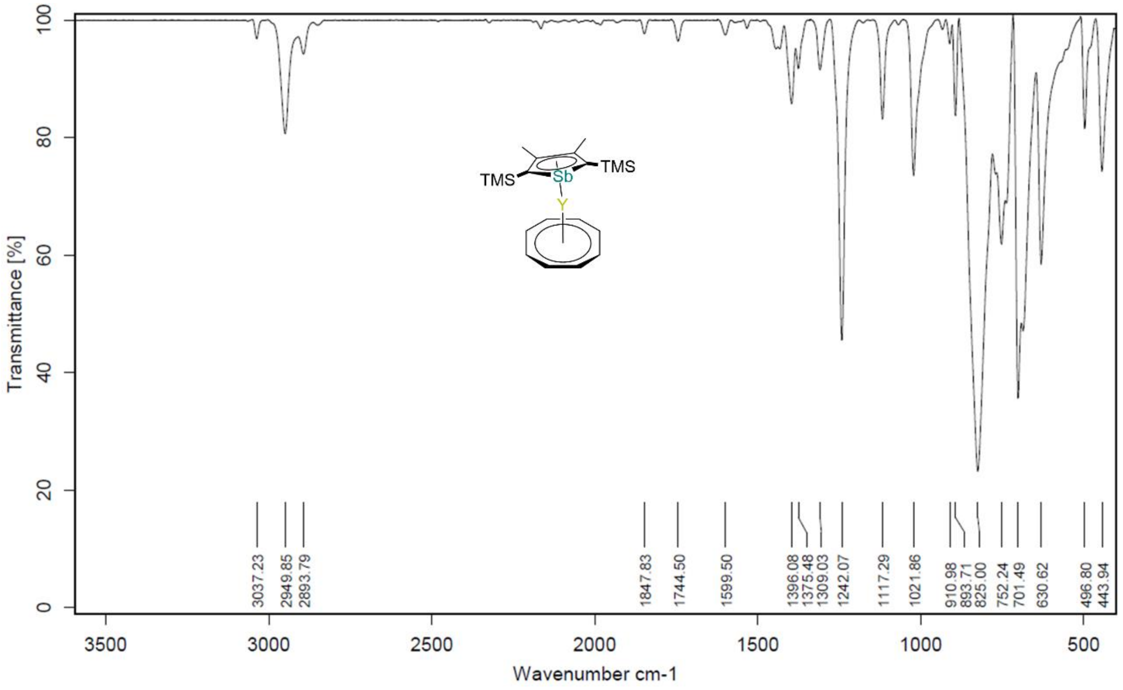

Figure S17: IR spectrum of **4-Y [Y(COT)(dssb)].**

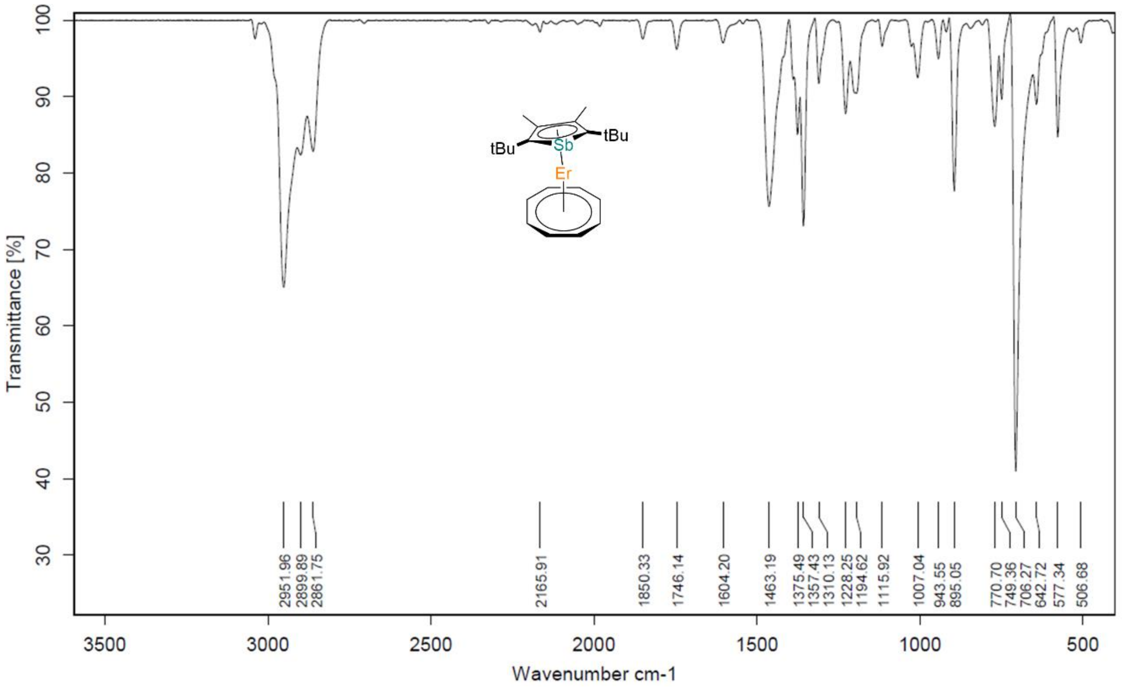

Figure S18: IR spectrum of **3-Er [Er(COT)(dtsb)].**

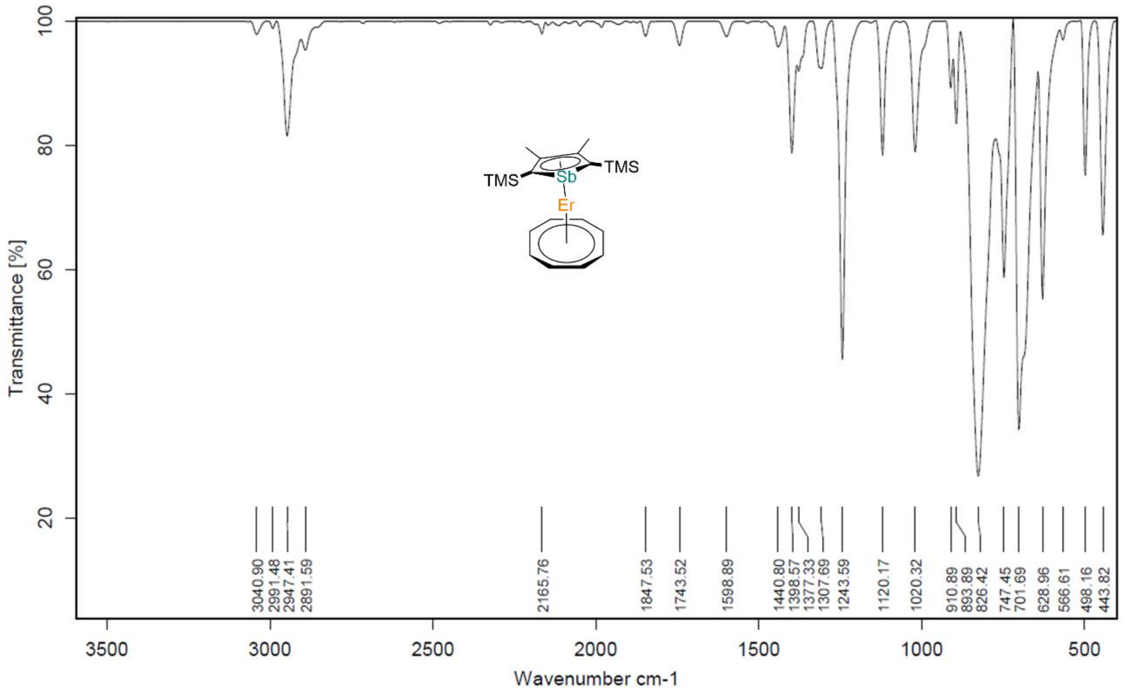

Figure S19: IR spectrum of **4-Er [Er(COT)(dssb)].**

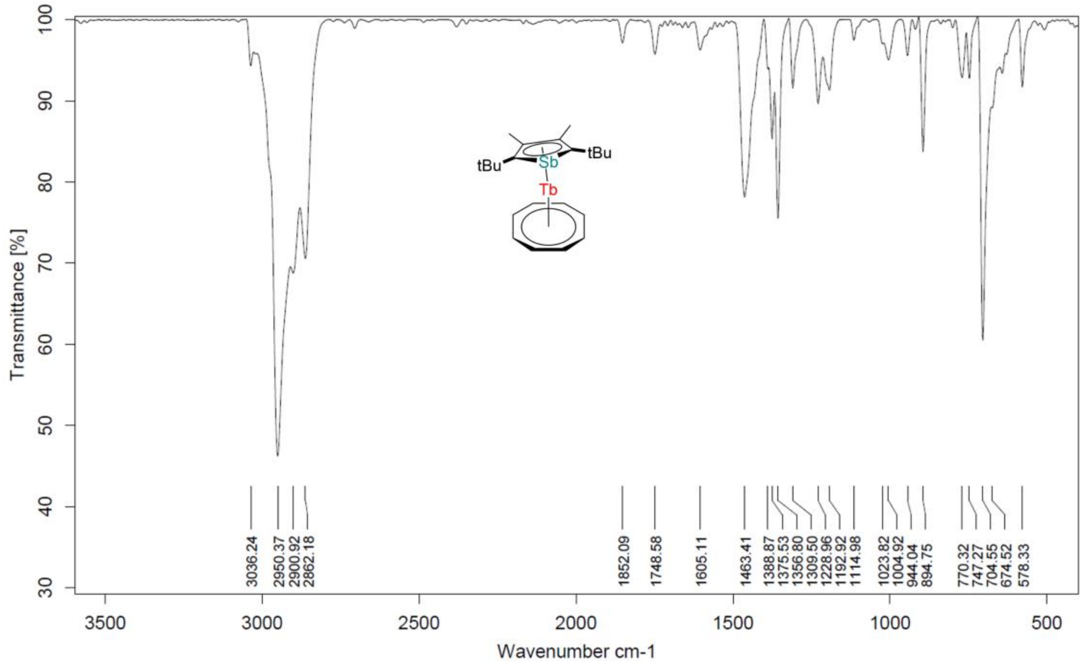

Figure S20: IR spectrum of **3-Tb [Tb(COT)(dtsb)].**

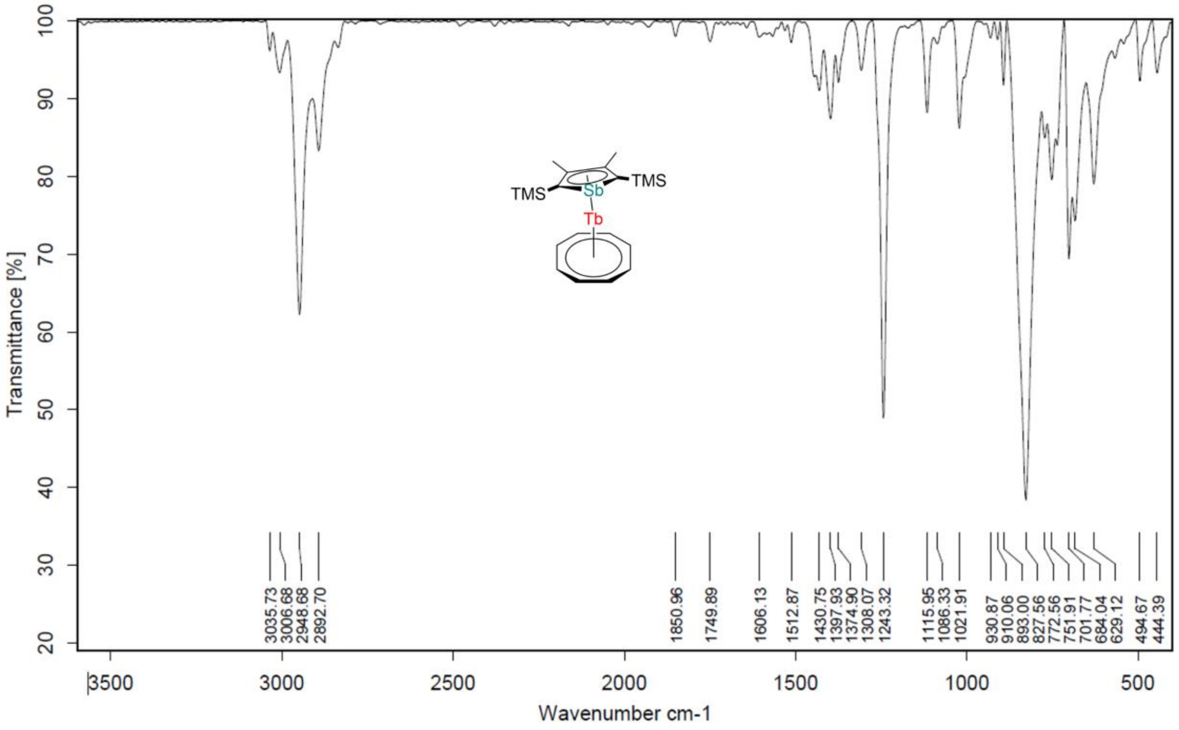

Figure S21: IR spectrum of **4-Tb [Tb(COT)(dssb)].**

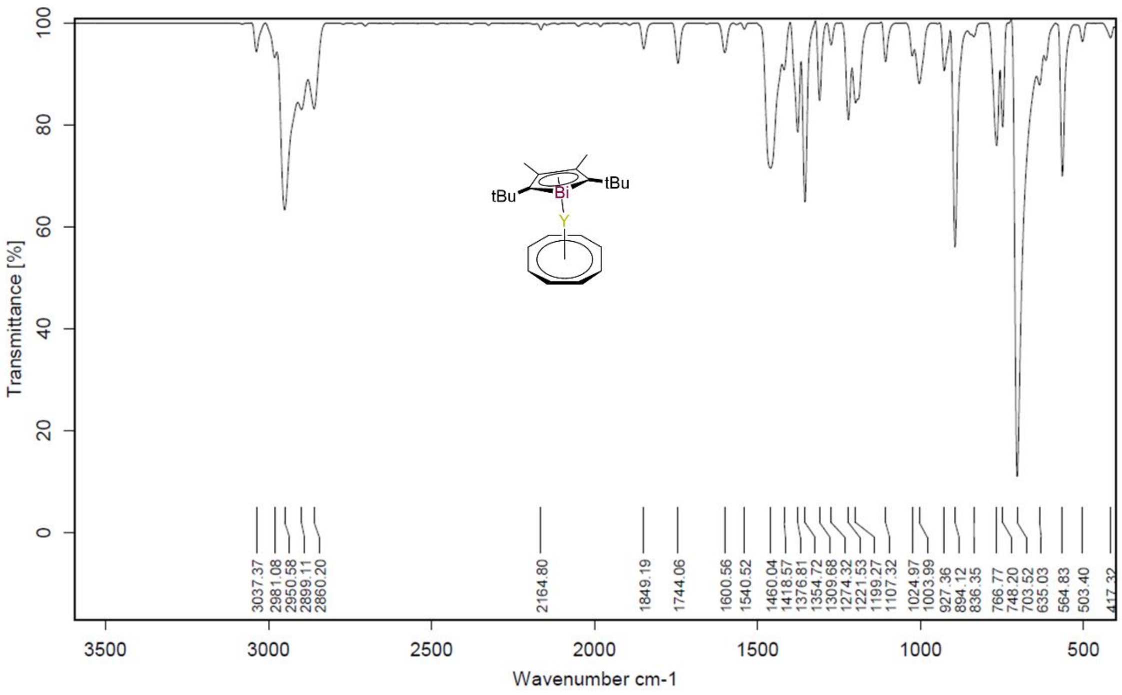

Figure S22: IR spectrum of **5-Y [Y(COT)(dtbi)].**

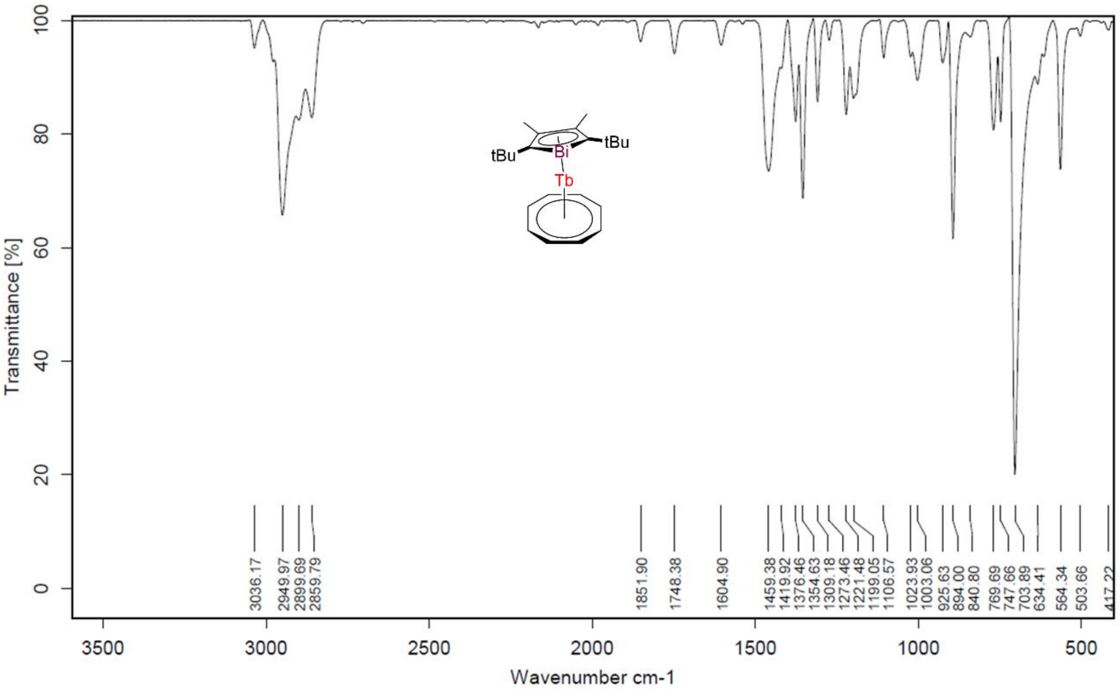

Figure S23: IR spectrum of **5-Tb [Tb(COT)(dtbi)].**

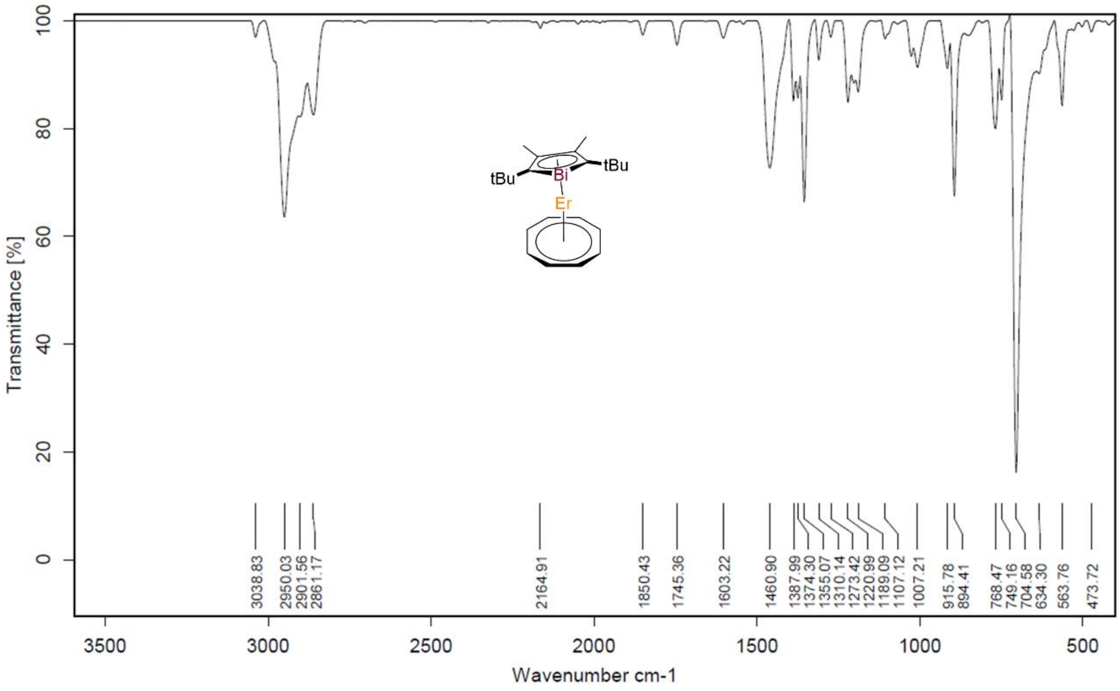

Figure S24: IR spectrum of **5-Er [Er(COT)(dtbi)].**

X-ray crystallography

General methods

Suitable crystals for the X-ray analysis of all compounds were obtained as described above. A suitable crystal was covered in mineral oil (Aldrich) and mounted on a glass fibre. The crystal was transferred directly to the cold stream of a STOE StadiVari (100 or 110 K) diffractometer. All structures were solved by using the program SHELXS/T and Olex2.[8-10] The remaining non-hydrogen atoms were located from successive difference Fourier map calculations. The refinements were carried out by using full-matrix least-squares techniques on F^2 by using the program SHELXL.[11] The H-atoms were introduced into the geometrically calculated positions (SHELXL procedures) unless otherwise stated and refined riding on the corresponding parent atoms. In each case, the locations of the largest peaks in the final difference Fourier map calculations, as well as the magnitude of the residual electron densities, were of no chemical significance. Specific comments for each data set are given below. Summary of the crystal data, data collection and refinement for compounds are given in Table S1. Crystallographic data for the structures reported in this paper have been deposited with the Cambridge Crystallographic Data Centre as a supplementary publication no. CCDC 2358764-2358770 and 2381646-2381647. Copies of the data can be obtained free of charge on application to CCDC, 12 Union Road, Cambridge CB21EZ, UK (fax: (+(44)1223-336-033; email: deposit@ccdc.cam.ac.uk)

The following special comments were applied to the models of the structures:

In the structure of **4-Er**, one $SiMe_3$ group (Si8, C68-C70) is disordered over two positions with an occupancy of 0.31/0.69.

Additionally, the CheckCIF of **4-Er** shows two B-level alerts due to a Fourier Truncation Error.

<u>Note</u>: While both the Y and Tb compounds and **3-Er** crystallize in the orthorhombic space group *Pnma* with the asymmetric unit containing half of the molecule, the Er compound **4-Er** crystallizes in the monoclinic space group $P2_1/c$. The asymmetric unit of **4-Er** consists of five independent molecules, each exhibiting slightly different geometries. Since none of the other compounds showed this crystallization behavior and all attempts to recrystallize the complex resulted in the same space group and asymmetric unit, we have deduced that packing effects may contribute to this unique behavior.

Summary of crystal data

Table S1: Crystal data, data collection and refinement for the rare-earth stibolyl bismolyl complexes **3-Y**, **3-Er**, **3-Tb**, **4-Y**, **4-Er**, **4-Tb**, **5-Y**, **5-Tb**, and **5-Er**.

Compounds	**3-Y**	**3-Er**	**3-Tb**	**4-Y**	**4-Er**	**4-Tb**	**5-Y**	**5-Tb**	**5-Er**
Chemical formula	$C_{22}H_{32}SbY$	$C_{22}H_{32}ErSb$	$C_{22}H_{32}SbTb$	$C_{20}H_{32}SbSi_2Y$	$C_{20}H_{32}ErSi_2Sb$	$C_{20}H_{32}SbSi_2Tb$	$C_{22}H_{32}BiY$	$C_{22}H_{32}BiTb$	$C_{22}H_{32}BiEr$
CCDC Number	2358764	2358765	2381646	2358766	2358767	2381647	2358768	2358769	2358770
Formula Mass	507.13	585.48	577.18	539.29	617.64	609.33	594.36	664.37	672.71
Crystal system	orthorhombic	orthorhombic	orthorhombic	orthorhombic	monoclinic	orthorhombic	orthorhombic	orthorhombic	orthorhombic
a/Å	12.5007(10)	12.5174(6)	12.5494(6)	12.706(2)	34.5947(7)	12.8317(6)	12.6035(6)	12.6225(3)	12.6117(8)
b/Å	17.743(2)	17.8450(11)	17.8216(8)	19.205(2)	10.6214(2)	19.2599(13)	17.8923(8)	17.8836(5)	17.9464(9)
c/Å	9.2990(10)	9.2658(5)	9.3169(6)	9.1893(12)	32.3168(7)	9.3113(4)	9.2740(6)	9.2957(3)	9.2513(4)
α/°									
β/°					99.871(2)				
γ/°									
Unit cell volume/Å^3	2062.5(3)	2070.8(2)	2083.7(2)	2242.4(5)	11698.8(4)	2301.2(2)	2091.3(2)	2098.37(10)	2093.9(2)
Temperature/K	100	110	110	110	100	110	100	100	100
Space group	*Pnma*	*Pnma*	*Pnma*	*Pnma*	$P2_1/c$	*Pnma*	*Pnma*	*Pnma*	*Pnma*
No. of formula units per unit cell, Z	4	4	4	4	20	4	4	4	4
Absorption coefficient, μ/mm^{-1}	4.110	5.330	4.663	3.887	4.819	4.326	11.165	11.718	12.374
No. of reflections measured	5933	8849	8284	9103	87762	8582	7034	19825	6539
No. of independent reflections	1978	2697	2680	2280	21117	2572	1950	2010	1957
R_{int}	0.0384	0.0207	0.0299	0.0350	0.0250	0.0304	0.0520	0.0397	0.0493
Final R_1 values ($I > 2\sigma(I)$)	0.0396	0.0260	0.0241	0.0310	0.0314	0.0312	0.0433	0.0190	0.0467
Final $wR(F^2)$ values ($I > 2\sigma(I)$)	0.0953	0.0728	0.0538	0.0671	0.0697	0.0785	0.1121	0.0165	0.1228
Final R_1 values (all data)	0.0500	0.0285	0.0329	0.0398	0.0488	0.0347	0.0523	0.0221	0.0550
Final $wR(F^2)$ values (all data)	0.1013	0.0748	0.0549	0.0716	0.0780	0.0793	0.1194	0.0461	0.1286
Goodness of fit on F^2	1.035	1.085	0.958	1.042	1.006	1.016	1.063	1.056	1.059

Crystal structures

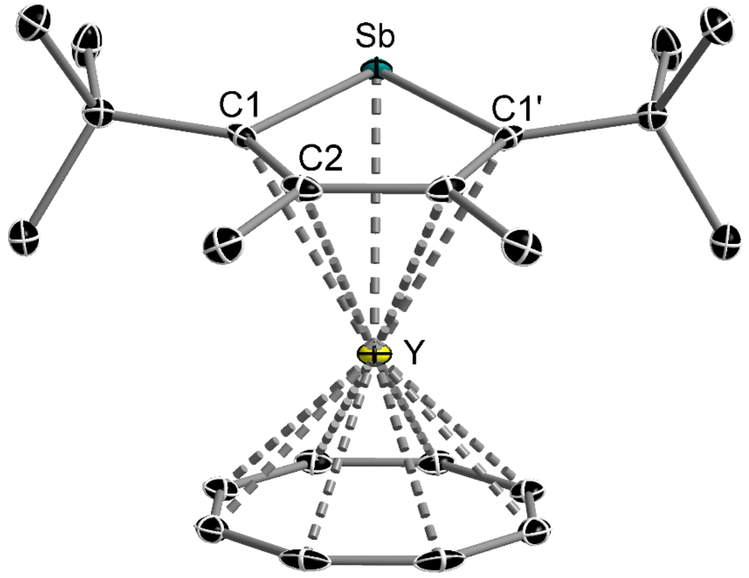

Figure S25: Molecular structure of **3-Y** (CCDC: 2358764) in the solid state with ellipsoids drawn at 30% probability. Hydrogen atoms are omitted for clarity. Selected bond lengths [Å] and angles [°]: Sb-Y 3.1141(8), Y-C_{COT} 2.514(5)-2.524(4), Y-Ct_{COT} 1.731(2), Y-C1 2.776(4), Y-C2 2.659(4), Y-Ct_{Dtsb} 2.408(3), Ct_{COT}-Y-Ct_{Dtsb} 169.38(10).

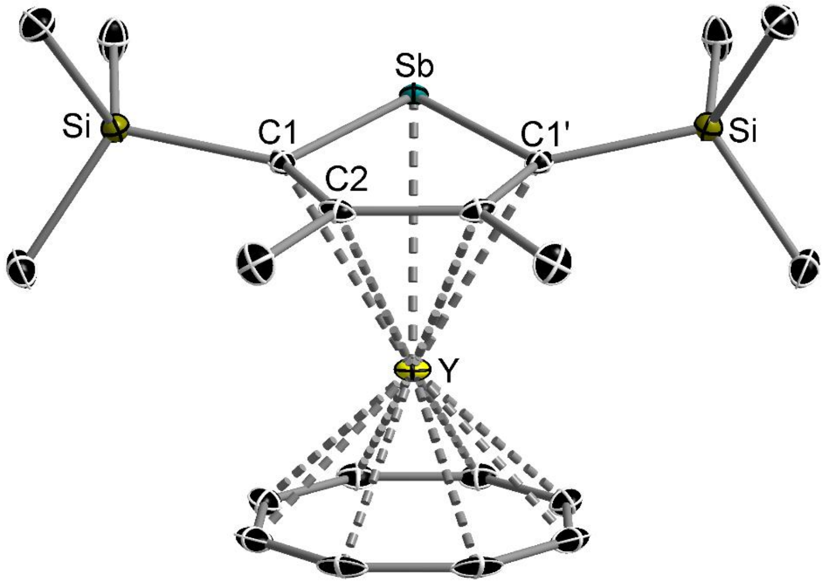

Figure S26: Molecular structure of **4-Y** (CCDC: 2358766) in the solid state with ellipsoids drawn at 30% probability. Hydrogen atoms are omitted for clarity. Selected bond lengths [Å] and angles [°]: Sb-Y 3.0795(7), Y-C_{COT} 2.489(4)-2.514(4), Y-Ct_{COT} 1.714(2), Y-C1 2.731(3), Y-C2 2.648(3), Y-Ct_{Dssb} 2.376(2), Ct_{COT}-Y-Ct_{Dssb} 168.92(3).

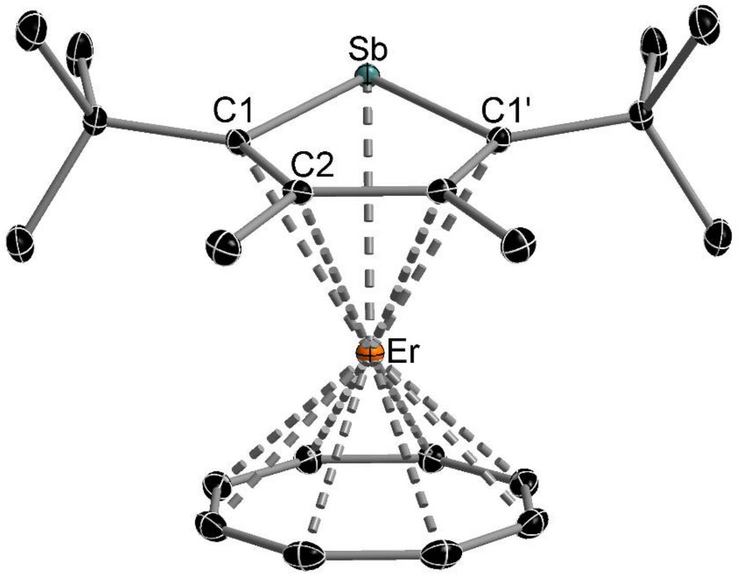

Figure S27: Molecular structure of **3-Er** (CCDC: 2358765) in the solid state with ellipsoids drawn at 30% probability. Hydrogen atoms are omitted for clarity. Selected bond lengths [Å] and angles [°]: Sb-Er 3.0883(4), Er-C_{COT} 2.509(3)-2.524(4), Er-Ct_{COT} 1.714(2), Er-C1 2.757(3), Er-C2 2.650(2), Er-Ct_{Dtsb} 2.385(2), Ct_{COT}-Er-Ct_{Dtsb} 169.440(12).

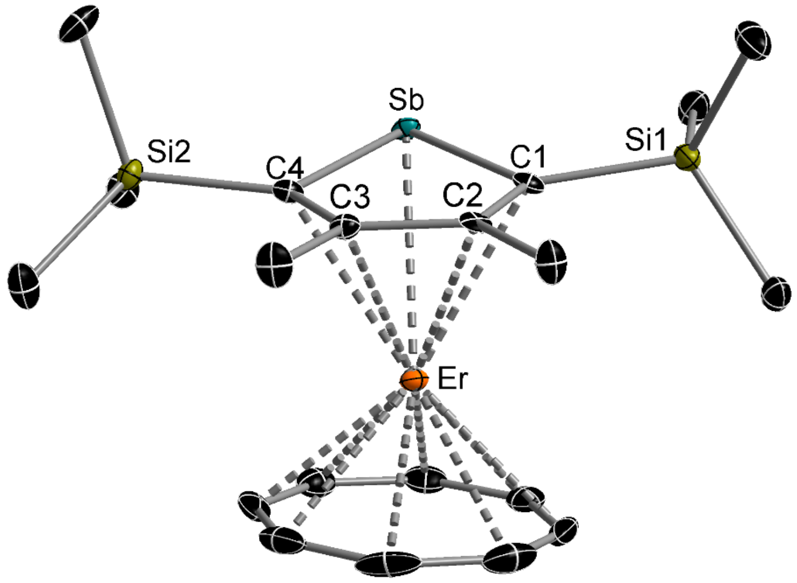

Figure S28: Molecular structure of **4-Er** (CCDC: 2358767) in the solid state with ellipsoids drawn at 30% probability. Hydrogen atoms are omitted for clarity. Only one molecule of the asymmetric unit is depicted. Selected bond lengths [Å] and angles [°] are displayed in Table S2.

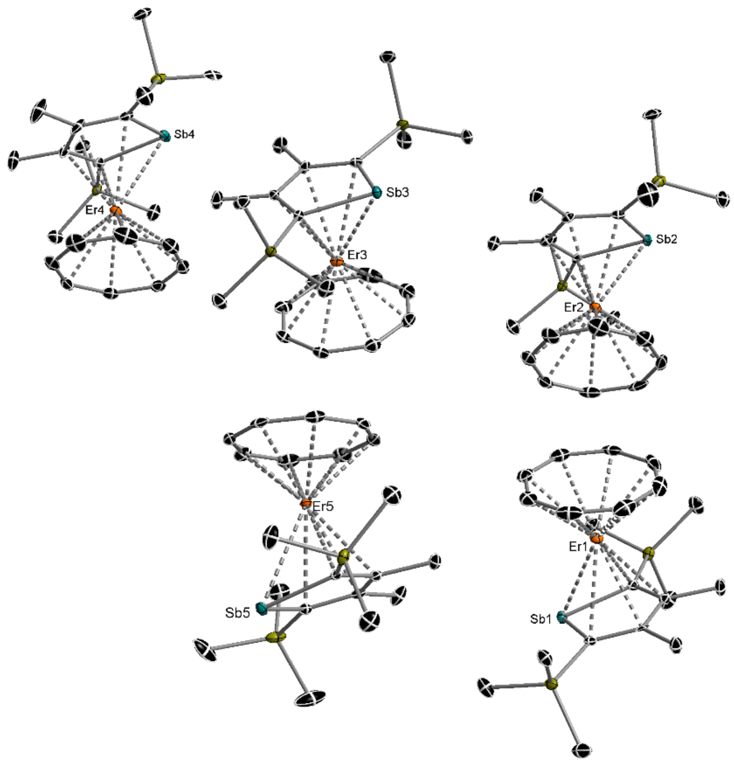

Figure S29: Asymmetric unit of **4-Er** (CCDC: 2358767) in the solid state with ellipsoids drawn at 30% probability. Hydrogen atoms are omitted for clarity. Selected bond lengths [Å] and angles [°] are displayed in the table below.

Table S2: Selected bond lengths and angles of all molecules in the asymmetric unit of **[Er(COT)(Dssb)]** (**4-Er**).

	1	2	3	4	5
Er-Sb [Å]	3.0636(4)	3.0478(4)	3.0593(4)	3.0563(4)	3.0595(4)
Er-C_{COT} [Å]	2.481(6)-2.499(6)	2.484(6)-2.509(6)	2.481(6)-2.508(5)	2.491(6)-2.505(6)	2.485(5)-2.510(5)
Er-Ct_{COT} [Å]	1.6864	1.6925	1.6937	1.6912	1.692
Er-Ct_{Dssb} [Å]	2.3473	2.3377	2.3476	2.3384	2.3473
Ct_{COT}-Er-Ct_{Dssb} [°]	170.334(2)	175.046(1)	178.415(1)	175.505(1)	170.373(2)

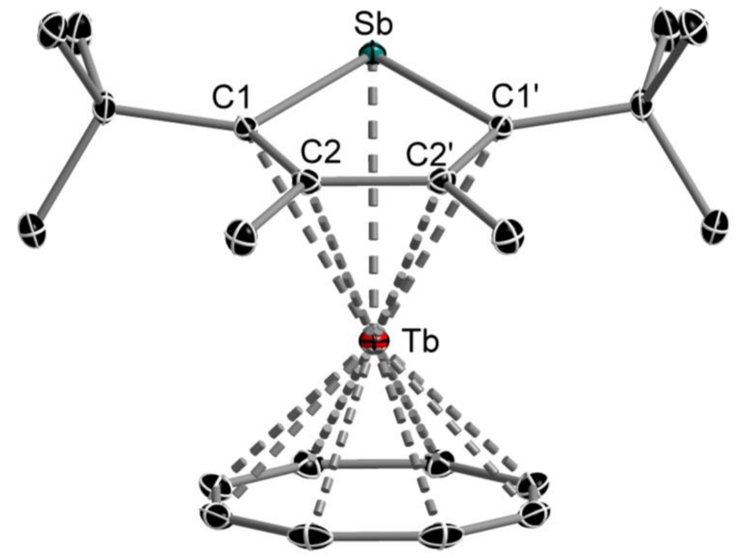

Figure S30: Molecular structure of **3-Tb** (CCDC: 2381646) in the solid state with ellipsoids drawn at 30% probability. Hydrogen atoms are omitted for clarity. Selected bond lengths [Å] and angles [°]: Sb-Tb 3.1283(4), Tb-C_{COT} 2.550(3)-2.557(3), Tb-Ct_{COT} 1.7622(14), Tb-C1 2.809(3), Tb-C2 2.699(3), Tb-Ct_{Dtsb} 2.442(2), Ct_{COT}-Tb-Ct_{Dtsb} 168.62(6).

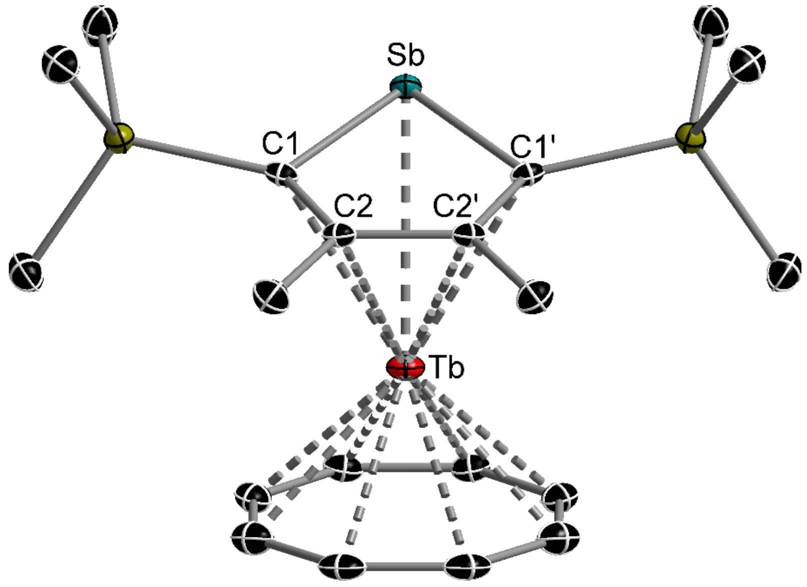

Figure S31: Molecular structure of **4-Tb** (CCDC: 2381647) in the solid state with ellipsoids drawn at 30% probability. Hydrogen atoms are omitted for clarity. Selected bond lengths [Å] and angles [°]: Sb-Tb 3.1286(4), Tb-C_{COT} 2.538(4)-2.556(4), Tb-Ct_{COT} 1.759(2), Tb-C1 2.784(3), Tb-C2 2.695(3), Tb-Ct_{Dssb} 2.4286(2), Ct_{COT}-Tb-Ct_{Dssb} 168.08(7).

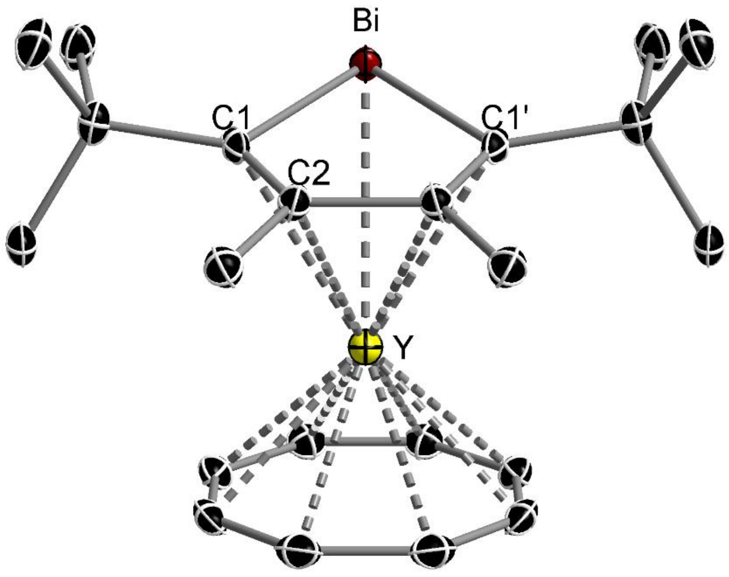

Figure S32: Molecular structure of **5-Y** (CCDC: 2358768) in the solid state with ellipsoids drawn at 30% probability. Hydrogen atoms are omitted for clarity. Selected bond lengths [Å] and angles [°]: Bi-Y 3.1716(2), Y-C_{COT} 2.517(8)-2.542(8), Y-Ct_{COT} 1.738(4), Y-C1 2.772(7), Y-C2 2.675(7), Y-Ct_{Dtbi} 2.405(4), Ct_{COT}-Y-Ct_{Dtbi} 169.63(2).

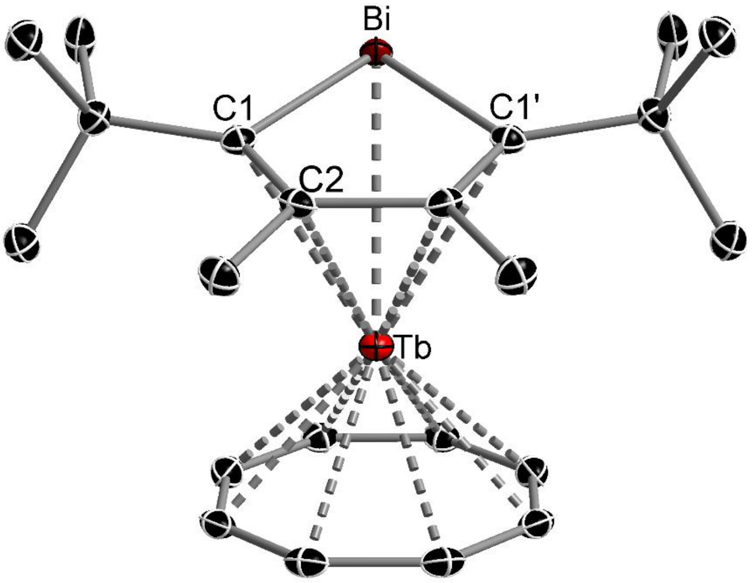

Figure S33: Molecular structure of **5-Tb** (CCDC: 2358769) in the solid state with ellipsoids drawn at 30% probability. Hydrogen atoms are omitted for clarity. Selected bond lengths [Å] and angles [°]: Bi-Tb 3.1879(3), Tb-C_{COT} 2.546(4)-2.555(4), Tb-Ct_{COT} 1.763(2), Tb-C1 2.805(3), Tb-C2 2.690(3), Tb-Ct_{Dtbi} 2.432(2), Ct_{COT}-Tb-Ct_{Dtbi} 168.594(1).

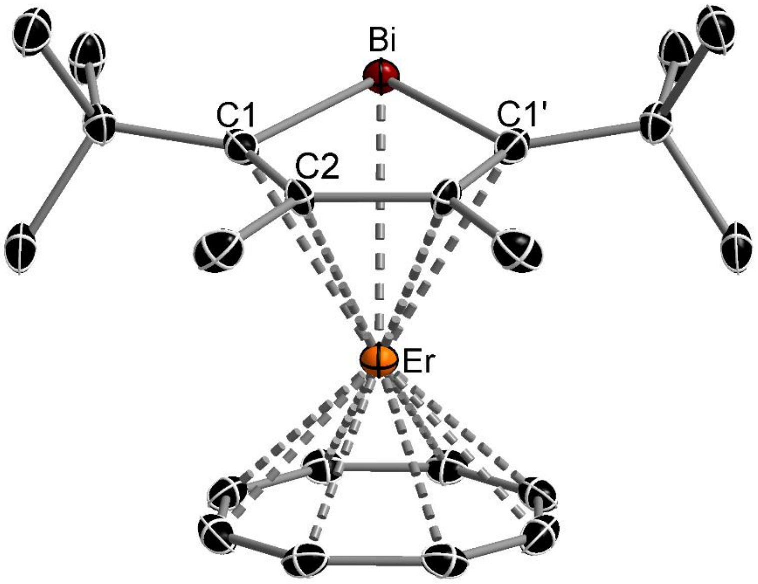

Figure S34: Molecular structure of **5-Er** (CCDC: 2358770) in the solid state with ellipsoids drawn at 30% probability. Hydrogen atoms are omitted for clarity. Selected bond lengths [Å] and angles [°]: Bi-Er 3.1502(8), Er-C_{COT} 2.487(9)-2.521(10), Er-Ct_{COT} 1.701(5), Er-C1 2.761(9), Er-C2 2.638(8), Er-Ct_{Dtbi} 2.374(5), Ct_{COT}-Er-Ct_{Dtbi} 169.4(2).

Supplementary Notes

Quantum Chemical Calculations

Calculations were performed with TURBOMOLE[12] at the def2-TZVP[13,14]/PBE0[15,16] level with grid 3.[17] Effective core potentials (ECPs) were employed for the description of the heavy elements, namely an ECP-60[18] for Bi, an ECP-28[18] for Sb, an ECP-28[19] for Er and Tb and an ECP-28[20] for Y. As usual, the ECP for Bi is used in a modified form within TURBOMOLE, which does not include the g part of the ECP.[13] A self-consistent field threshold of 10^{-8} E_h was chosen for all calculations. Structures were optimized in C_s symmetry with thresholds of 10^{-6} E_h and 10^{-3} E_h/a_0 and with use of the D4 dispersion correction.[21] The final calculations of the energies and the current densities were done without exploiting the symmetry. All calculations employed the multipole accelerated RI-*J* (MARI-*J*) approximation[22,23] with the def2-TZVP auxiliary basis sets.[14,24] The origin of the Cartesian coordinates was chosen to be in the middle of the bismolyl/stibolyl ring and the molecule was oriented in a way that the C-C bond opposite to the Bi/Sb atom is directed parallel to the x-axis and that the z-direction is perpendicular to the ring. The calculation of the current densities was done with the GIMIC program[25-27] using the perturbed densities calculated by the module mpshift[22,28] of TURBOMOLE with a threshold of 10^{-7} for the norm of the residuum in the coupled-perturbed Kohn-Sham procedure.

In Table S3, calculated ring currents for all complexes are listed. For the numerical integration of the currents, the [0.19, 0.19, 1] spacing within GIMIC was chosen. Cur1 denotes the current in the bismolyl/stibolyl ring within the sandwich complex and Cur2 that in the bismolyl/stibolyl anion separated from the complex but with the same structure and integration boundaries as in the complex. Details on the choice of the integration boundaries are described in later paragraphs and in Table S4. Cur3 denotes the current for the bare bismolyl/stibolyl anion obtained with the default integration area set by GIMIC.

Table S3: Calculated ring currents for different bismolyl/stibolyl rings. Cur1: Current of the bismolyl/stibolyl ring within the complex. Cur2: Current of the sole bismolyl/stibolyl ring, calculated with the same smaller integration boundaries used for Cur1, which are needed to minimize the influence of the central atom. Cur3: Current of the sole bismolyl/stibolyl ring with greater integration boundaries. Details on the integration boundaries can be found in Table S4 and the following paragraphs.

	Cur1 / nA/T	Cur2 / nA/T	Cur3 / nA/T
**5-Er**	8.5	8.1	8.4
**5-Tb**	8.3	7.9	8.4
**5-Y**	8.8	8.1	8.5
**3-Er**	9.1	8.8	9.2
**4-Er**	10.2	10.2	10.6
**3-Y**	9.2	8.8	9.2
**4-Y**	10.5	10.1	10.6

A comparison between Cur2 and Cur3 demonstrates that most of the ring current flows within the boundaries chosen for the complex. The choices of the boundaries are detailed in the following paragraphs. For clarity, the directions of the different integration boundaries are depicted in Figure S35.

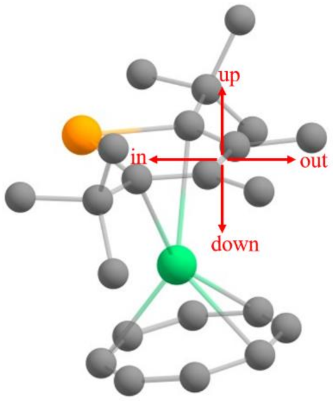

Figure S35: Schematic depiction of the directions of the different integration boundaries for the calculation of the current densities. As an example, **5-Er** is shown. Hydrogen atoms are omitted for clarity. Black: C, orange: Bi, green Er.

To minimize the influence of the current arising from the central atom, the integration area must be cut off before the influence of this atom grows too large. This means that a reasonable integration boundary in the “down” direction in Figure S35 has to be chosen. To determine such a boundary, different schemes for the different central atoms had to be employed. In case of Er, the atomic ring current – and thus the contribution to Cur1 arising from the central atom – is mainly paratropic, which means that the total ring current of the bismolyl/stibolyl ligand starts to decrease, if the integration boundary “down” in Figure S35 comes close to the atom. Therefore, the results for the total bismolyl/stibolyl ring currents were screened by increasing the integration boundary towards the central atom in small steps of 0.05 a_0. The last maximum value for the total ring current before the starting decrease was chosen and the resulting integration boundary was also used for the calculation of Cur2 to ensure comparability between the values. In case of Tb and Y in contrast, the atomic ring current – and thus the contribution to Cur1 arising from the central atom – is mainly diatropic, which means it increases the ring current in an erroneous way. In order to choose an integration boundary, the fact that the growth of the ring current should decline with an increasing integration boundary was used. The ring currents were screened the same way as for the erbium complexes. The integration boundary of the saddle point of the resulting data, where the difference to the value with a smaller integration boundary is smaller than the difference to the value with the next greater integration boundary, was used for the determination of the ring current.

The chosen boundaries towards the central atoms are listed in Table S4. For the boundaries “up” and “out” in Figure S35 the default value of 8 a_0 provided by GIMIC was chosen. This value was also chosen for “down” for the calculation of Cur3 in Table S3. The integration boundary for “in” was chosen in a way that the boundary reached approximately the center of the ring current. The boundaries for “in” are also listed in Table S4. Here, in1 gives the boundary for the sandwich complex (used for Cur1 in Table S3) and in2 gives the value for the sole bismolyl/stibolyl ring (used for Cur2 and Cur3 in Table S3). The origin (0,0,0) was chosen as the fixed coordinate for the construction of the integration area within GIMIC.

Table S4: Chosen integration boundaries for the ring currents in Table S3. The directions of the boundaries are depicted in Figure S35. down: Boundary in the "down" direction towards the central atom of the sandwich complex (Cur1 and Cur2). in1: Boundary in the "in" direction for the sandwich complex (Cur1). in2: Boundary in the "in" direction for the sole bismolyl/stibolyl ring (Cur2 and Cur3). Boundaries not listed here were set to the default value of 8 a_0.

	down / a_0	in1 / a_0	in2 / a_0
**5-Er**	2.600	1.789	1.859
**5-Tb**	2.300	1.833	1.853
**5-Y**	2.450	1.827	1.857
**3-Er**	2.600	1.785	1.855
**4-Er**	2.550	1.788	1.848
**3-Y**	2.450	1.814	1.854
**4-Y**	2.450	1.807	1.847

The Mulliken overlap populations[29] discussed in the main text are listed in Table S5. For the open-shell compounds, the contributions of the α and β orbitals were summed up. The considered orbitals for the different compounds are depicted in Figures 4a and 4b in the main text. The two considered orbitals contain between 42% and 69% of the total Mulliken overlap population between Bi/Sb and the central atom.

Table S5: Mulliken overlap populations between Bi/Sb and the central atom for the HOMO, the energetically next MO with a significant Mulliken population of both Bi and the central atom (2nd MO) and for the sum of all orbitals (Total). For **5-Tb** the overlap in the second column is zero as here the orbital which exhibits a Bi or Sb population for all other compounds does not include a significant Bi population.

	HOMO	2nd MO	Total
**5-Er**	0.17	0.02	0.33
**5-Tb**	0.18	0.00	0.26
**5-Y**	0.14	0.03	0.34
**3-Er**	0.12	0.04	0.32
**4-Er**	0.04	0.11	0.35
**3-Y**	0.11	0.05	0.36
**4-Y**	0.02	0.12	0.30

In order to investigate which atomic orbitals of Bi/Sb and the respective central atom contribute to the bond, the contributions of these atoms were broken down into contributions from s, p, d and f orbitals according to a Mulliken population analysis. In case of the open-shell compounds, the contributions to the α- and β-MOs were added together. This was done for the HOMO of each compound (Table S6) as well as for the second relevant MO (Table S7). The contributions in Table S6 and Table S7 demonstrate that Bi and Sb mostly contribute with a p-type orbital to the considered MOs, while the central atom mostly contributes with a d-type orbital.

Table S6: Contributions of Bi/Sb and the respective central atoms to the HOMOs according to a Mulliken population analysis. In case of open-shell compounds, the contributions to the α- and β-HOMO were added together.

Molecule	Atom	Total	s	p	d	f
**5-Er**	Bi	1.141	-0.000	1.134	0.006	0.001
	Er	0.222	0.031	0.035	0.151	0.005
**5-Tb**	Bi	1.189	-0.000	1.181	0.006	0.001
	Tb	0.244	0.032	0.041	0.158	0.013
**5-Y**	Bi	1.119	-0.002	1.114	0.006	0.001
	Y	0.227	0.025	0.055	0.143	0.004
**3-Er**	Sb	0.945	0.000	0.937	0.006	0.001
	Er	0.211	0.020	0.028	0.155	0.008
**4-Er**	Sb	0.508	0.000	0.503	0.003	0.001
	Er	0.224	0.010	0.019	0.185	0.010
**3-Y**	Sb	0.975	-0.002	0.970	0.006	0.001
	Y	0.223	0.019	0.047	0.151	0.005
**4-Y**	Sb	0.412	-0.002	0.411	0.002	0.001
	Y	0.240	0.007	0.021	0.205	0.007

Table S7: Contributions of Bi/Sb and the respective central atoms to the second orbital. See also Table S6.

Molecule	Atom	Total	s	p	d	f
**5-Er**	Bi	0.041	0.001	0.039	0.000	0.000
	Er	0.272	0.002	0.001	0.256	0.013
**5-Tb**	Bi	0.000	0.000	0.000	0.000	0.000
	Tb	0.292	0.000	-0.000	0.272	0.020
**5-Y**	Bi	0.065	0.001	0.064	0.000	0.000
	Y	0.281	0.004	0.005	0.261	0.011
**3-Er**	Sb	0.182	0.002	0.179	0.001	0.000
	Er	0.275	0.006	0.003	0.253	0.012
**4-Er**	Sb	0.557	0.001	0.551	0.004	0.000
	Er	0.261	0.016	0.009	0.228	0.009
**3-Y**	Sb	0.160	0.001	0.157	0.001	0.000
	Y	0.276	0.006	0.010	0.249	0.011
**4-Y**	Sb	0.657	0.001	0.650	0.005	0.001
	Y	0.257	0.021	0.039	0.188	0.008

Table S8: Molecular structures. The Cartesian coordinates are listed in angstrom. The structure optimizations were done at the def2-TZVP/PBE0/MARI-*J* level in C_s symmetry. Energies were calculated without exploiting the symmetry. They are given in Hartree and are rounded to the eighth digit after the decimal.

This data can be found in a separate text file.

Magnetic Measurements

General Methodology

All measurements were carried out on a Quantum Design MPMS3 SQUID magnetometer. The samples were fixed to the bottom of a quartz tube with eicosane and flame sealed under vacuum afterwards. Ampules where fixed by two smaller glass tubes in a SQUID sample straw. Static magnetization and AC SQUID measurements were carried out with the DC scan mode (scan length 30 mm, 4 s scan time, 3 scans per measurement). Hysteresis measurements were carried out with a sweep rate of 200 Oe/s with stabilization at each field. AC squid measurements used a three-point measurement and a mixed time/cycle averaging with an applied alternating magnetic field of 2 Oe. Static relaxation decay measurements were performed in VSM mode with a peak amplitude of 1 mm and an averaging time of 0.5 s as described in [30]. Zero-field cooled (ZFC) and field cooled (FC) measurements were performed in VSM mode with a peak amplitude of 1 mm and an averaging time of 0.5 s. The ZFC measurements were performed by rapidly cooling down from room temperature to 1.8 K, applying a field of 1000 Oe measuring continuously and heating with a rate of 0.5 K/min. FC measurements were done analogous to the ZFC measurements, but with an applied field of 1000 Oe.

DC SQUID measurements were corrected for impurities with the aid of the overlap region in the susceptibility measurements between 50 and 70 K. Susceptibility measurements used different fields, 1000 Oe for low temperatures and 10000 Oe for high temperatures. The Overlap region was measured with both fields.

Analysis of the SQUID data used MATLAB version R2023a with the Curve Fitting Toolbox version 3.9, the Global Optimization toolbox version 4.8.1, the Optimization Toolbox version 9.5. and the Symbolic Math Toolbox version 9.3.

DC SQUID measurements

3-Er

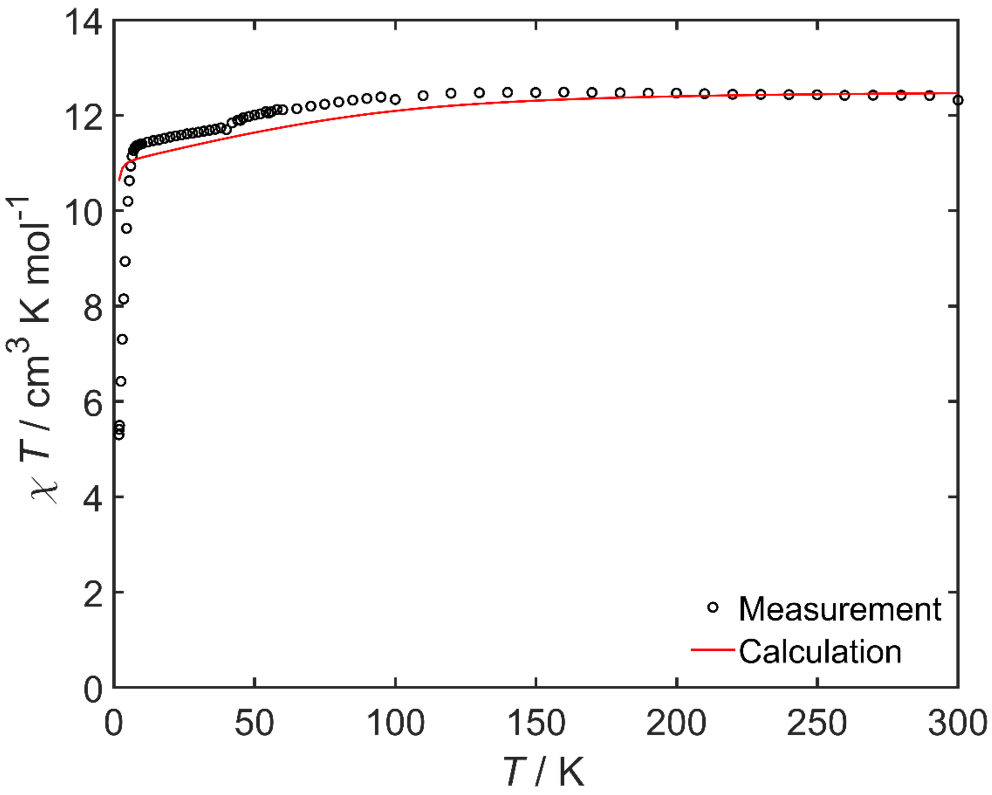

Figure S36: Temperature dependent magnetic susceptibility measurements of **3-Er** plotted as the product of molar susceptibility with the temperature. Calculated χT values for each temperature were scaled with a factor of 1.09 and are shown in red. Measurement was done starting at high temperature and slowly cooling down by stabilizing at each temperature.

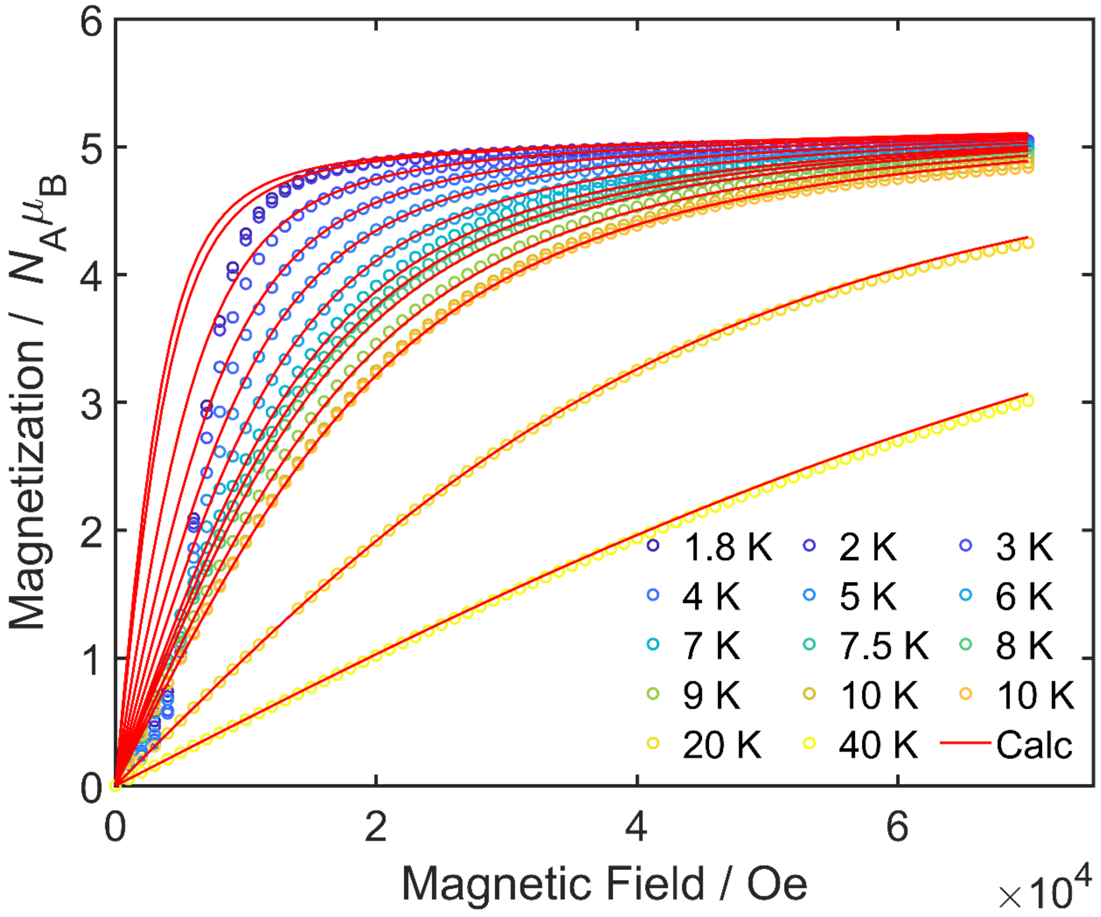

Figure S37: Measurement of the magnetization of **3-Er** dependent on the applied DC field at different temperatures. Calculated Magnetization values for each temperature and field were scaled with a factor of 1.09 and are shown in red. Measurement data below 10 K except for the measurement at 7.5 K was directly extracted from the Hysteresis measurement.

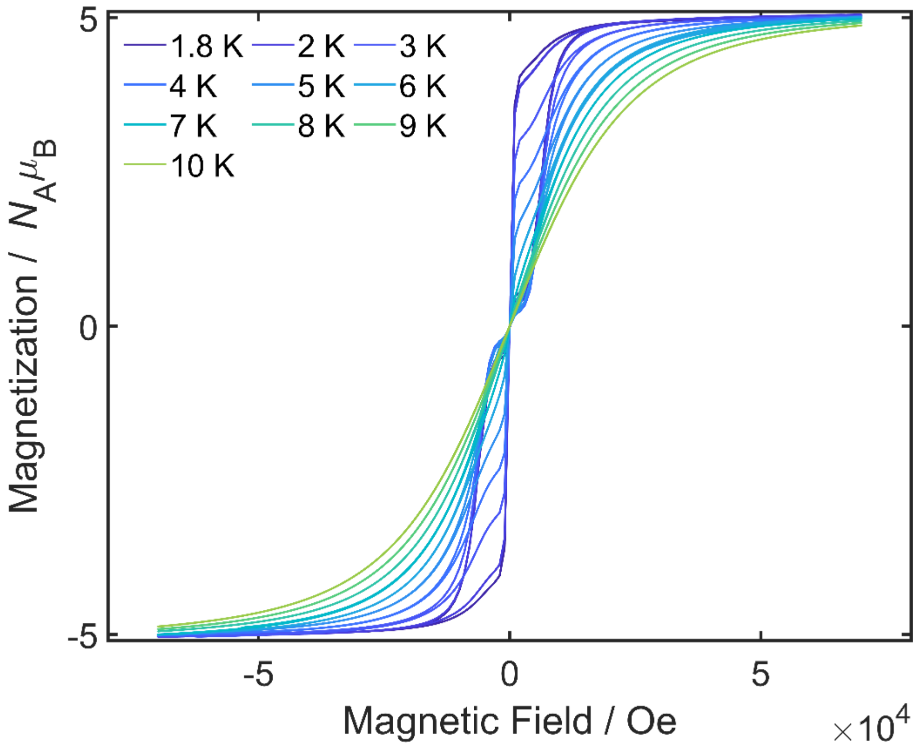

Figure S38: Temperature dependent hysteresis measurements of **3-Er**. The magnetic field was stabilized at each measured data point.

4-Er

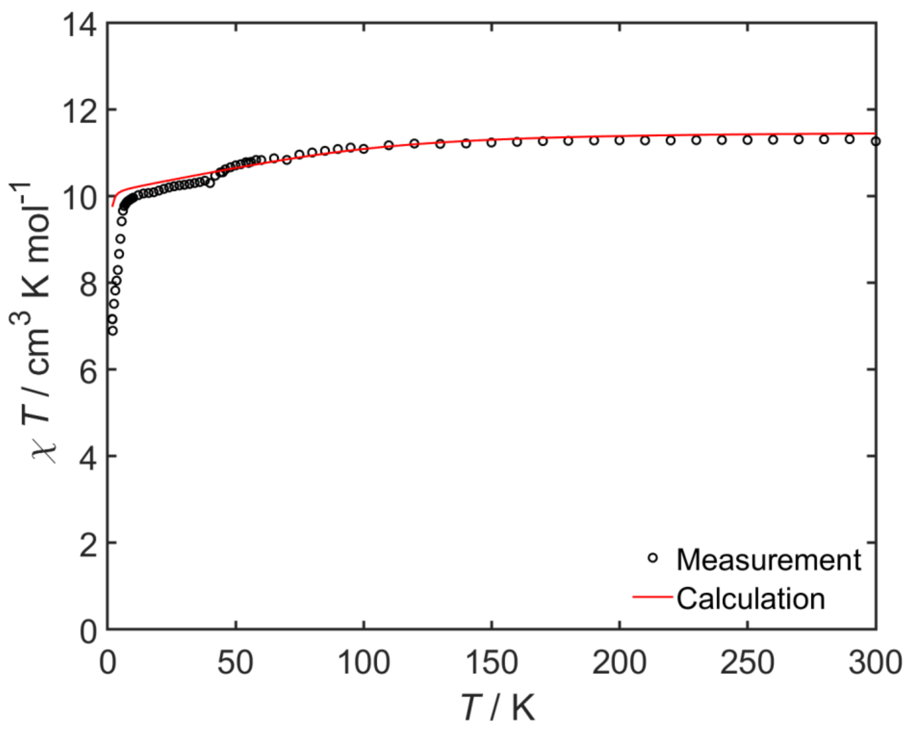

Figure S39: Temperature dependent magnetic susceptibility measurements of **4-Er** plotted as the product of molar susceptibility with the temperature. Calculated χT values for each temperature are shown in red. Measurement was done starting at high temperature and slowly cooling down by stabilizing at each temperature.

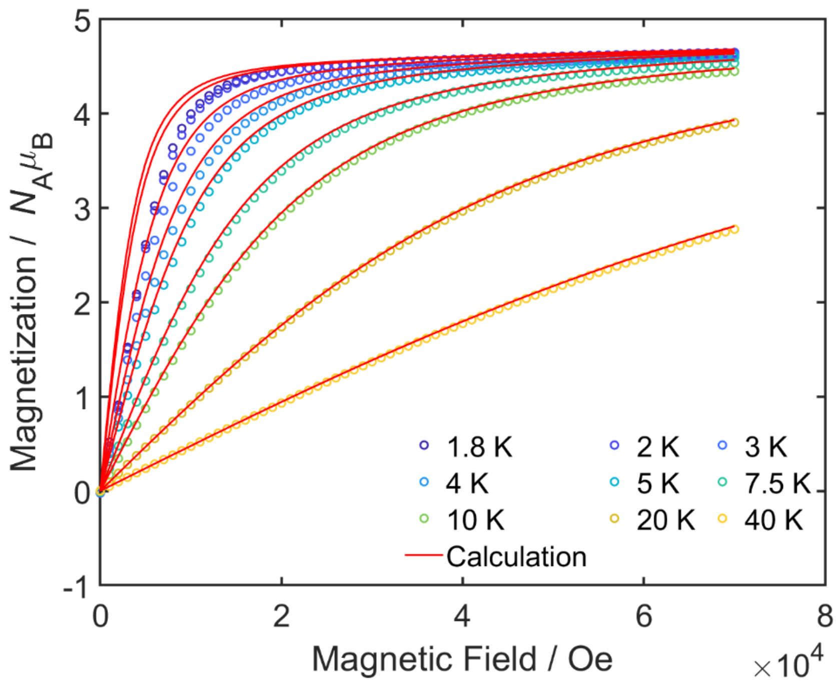

Figure S40: Measurement of the magnetization of **4-Er** dependent on the applied DC field at different temperatures. Calculated Magnetization values for each temperature and field are shown in red. Measurement Data below 5 K was directly extracted from the hysteresis measurement.

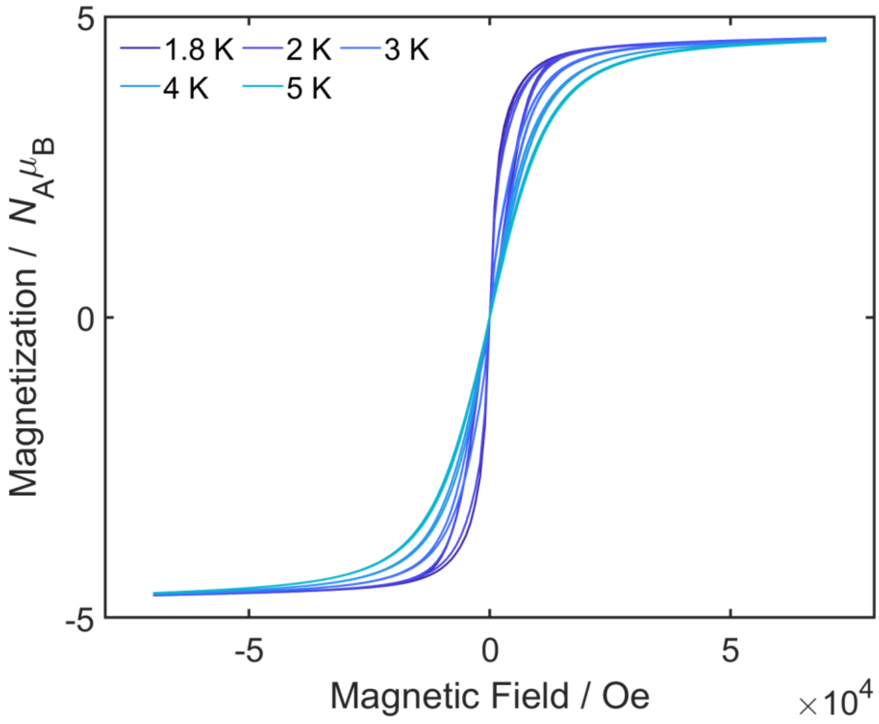

Figure S41: Temperature dependent hysteresis measurements of **4-Er**. The magnetic field was stabilized at each measured data point.

5-Er

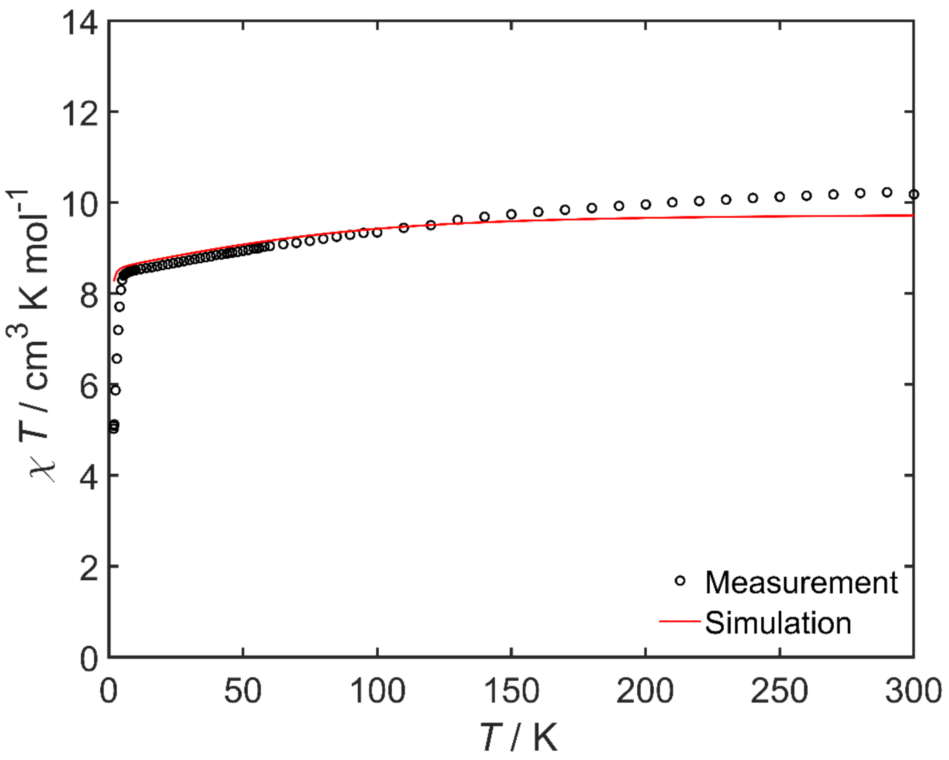

Figure S42: Temperature dependent magnetic susceptibility measurements of **5-Er** plotted as the product of molar susceptibility with the temperature. Calculated χT values for each temperature were scaled with a factor of 0.85 and are shown in red. Measurement was done starting at high temperature and slowly cooling down by stabilizing at each temperature.

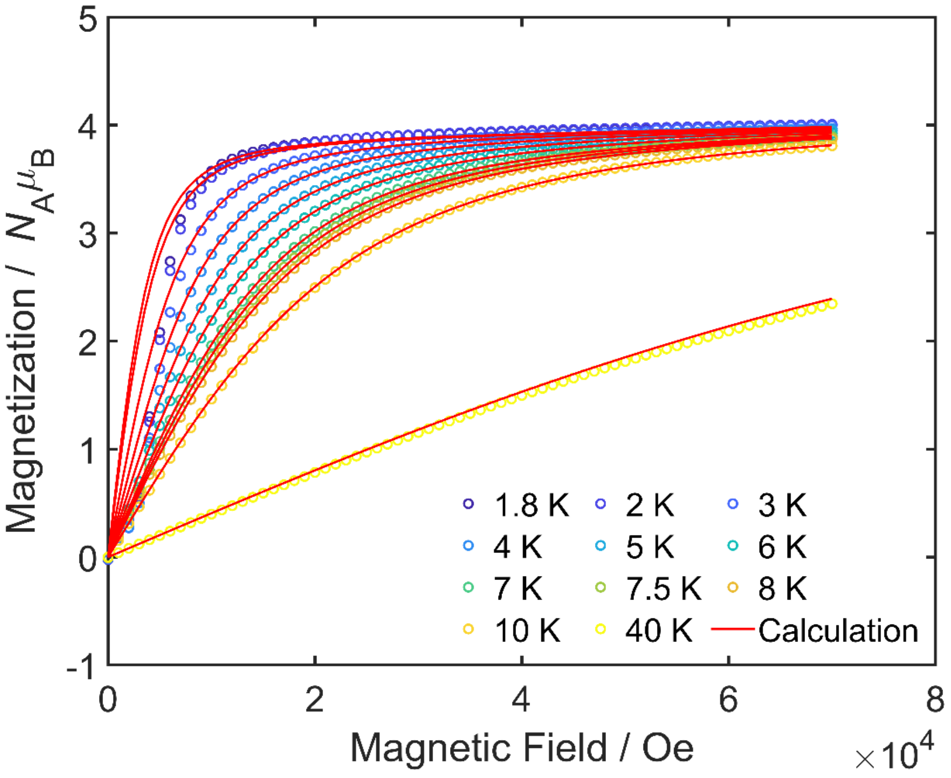

Figure S43: Measurement of the magnetization of **5-Er** dependent on the applied DC field at different temperatures. Calculated Magnetization values for each temperature and field were scaled with a factor of 1.09 and are shown in red. Measurement Data below 8 K, except at 7.5 K, was directly extracted from the hysteresis measurement.

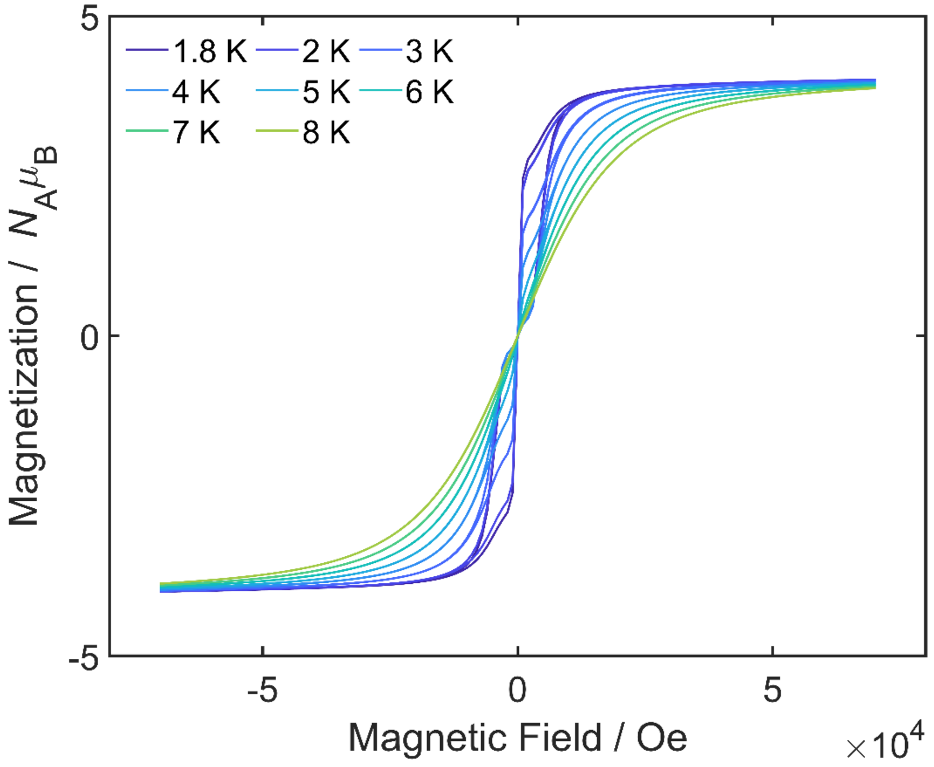

Figure S44: Temperature dependent hysteresis measurements of **5-Er**. The magnetic field was stabilized at each measured data point.

AC SQUID and DC relaxation measurements

The dynamic magnetization data was corrected for diamagnetic and impurity contributions using the same values as the static field measurements. The measured data was fit using the extended Debye model with Equation S1 for the in-phase molar susceptibility and Equation S2 for the out-of-phase molar susceptibility.[31] In this case $\omega = 2\pi\nu$, where ν is the frequency of the applied alternating magnetic field.

$$\chi'(\omega) = \chi_S + (\chi_T - \chi_S)\frac{1+(\omega\tau)^{1-\alpha}\, sin\left(\frac{\pi\alpha}{2}\right)}{1+2(\omega\tau)^{1-\alpha}\, sin\left(\frac{\pi\alpha}{2}\right)+(\omega\tau)^{2-2\alpha}} \quad \text{(S1)}$$

$$\chi''(\omega) = (\chi_T - \chi_S)\frac{(\omega\tau)^{1-\alpha}\, cos\left(\frac{\pi\alpha}{2}\right)}{1+2(\omega\tau)^{1-\alpha}\, sin\left(\frac{\pi\alpha}{2}\right)+(\omega\tau)^{2-2\alpha}} \quad \text{(S2)}$$

The relaxation times were extracted by simultaneous fit these formulas to the in- and out-of-phase data. For the relaxation times, the standard deviation was calculated using the fitted α parameter and Equation S3 as described in [30] and [32].

$$\sigma^2_{\langle\ln\tau\rangle} = \left(\frac{1}{(1-\alpha)^2} - 1\right)\frac{\pi^2}{3} \quad \text{(S3)}$$

Using Equation S3, the upper and lower limit of the estimated standard deviation of τ was calculated according to Equation S4

$$\tau_{\pm} = \exp\left(\langle\ln\tau\rangle \pm \sqrt{\sigma^2_{\langle\ln\tau\rangle}}\right) \quad \text{(S4)}$$

To determine the ideal applied static field strength for in-field AC SQUID susceptometry, field-dependent measurements were carried out. For **3-Er** a large out of phase signal was measured at 16 K and fields above 1000 Oe (Figure S49 and Figure S50). Similar observations were made for 4-Er (Figure S60 and Figure S61) and 5-Er (Figure S70 and Figure S71). Hence, for further in-field AC susceptometry measurements, a static field strength of 1500 Oe was chosen for **3-Er** and 1000 Oe for **4-Er** and **5-Er.**

For the DC relaxation measurements, the analysis was performed as described in[30]. Samples were magnetized for 15 min. Afterwards; the field was ramped down to the target field value with 700 Oe/s. During this, data was collected continuously and averaged every 0.5 seconds. Data analysis was carried out on the basis of Eq. S5

$$\mathrm{M}(t) = M_{\mathrm{eq}} + \left(M_0 - M_{\mathrm{eq}}\right)\exp\left[-\left(\frac{t}{\tau^*}\right)^{\beta}\right] \quad \text{(S5)}$$

Here, M_{eq} is the equilibrium magnetization at the target field, M_0 is the initial magnetization when the field has reached a stable value, t is the waited time, τ^* is the relaxation time and β the stretch factor. β can vary between 0 and 1, with $\beta=1$ being a monoexoponential decay. Low values of β indicate a wide spread of relaxation times. For M_0, values were chosen for the timestamp of the magnetization decay at which both the applied field and the third derivative of the magnetization curve reached 0. For M_{eq} the mean of the magnetization of the last 10 datapoints was used for the experiments where a full decay was measured. For the not completely measured decays, the value was, in dependence of the goodness of the fit, either allowed to fit freely, or a boundary restraint chosen by hand. As no measurement followed a strict monoexponential decay, the relaxation times needed to be converted

to compare them with the ones from the extended Debye model (Tables S9, S13 and S15). Thus, the values were converted to the logarithmic expectation value using Equation S6.[30,32]

$$\langle \ln[\tau] \rangle = \left(1 - \frac{1}{\beta}\right) \mathrm{Eu} + \ln[\tau^*] \qquad \text{(S6)}$$

Eu is the Euler constant.

All calculated values and errors are listed in their respective table.

3-Er

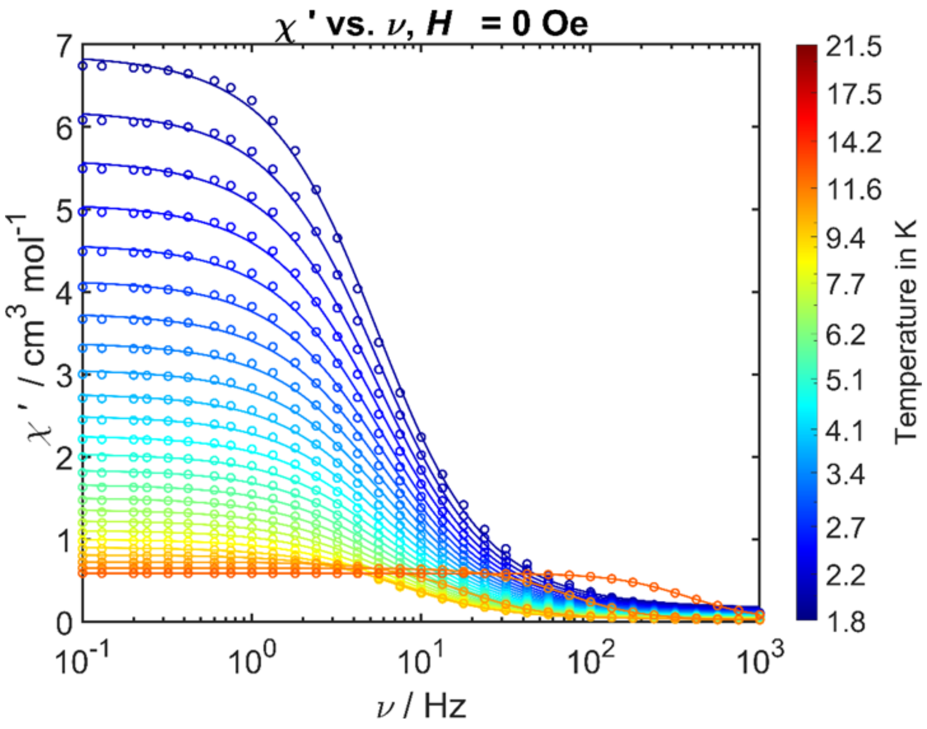

Figure S45: In-Phase molar magnetic susceptibility measurement of **3-Er** in dependence on the frequency of an applied alternating field with a magnitude of 2 Oe at different temperatures. The lines are fits according to Eq. S1. The resulting parameters are listed in Table S9.

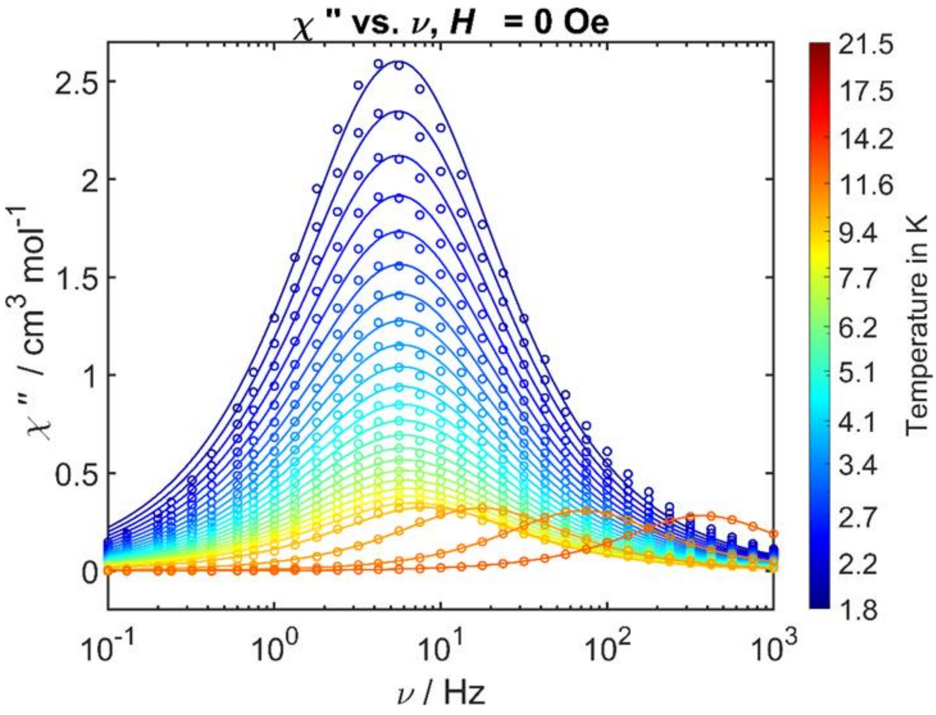

Figure S46: Out-of-Phase molar magnetic susceptibility measurement of **3-Er** in dependence on the frequency of an applied alternating field with a magnitude of 2 Oe at different temperatures. The lines are fits according to Eq. S2. The resulting parameters are listed in Table S9.

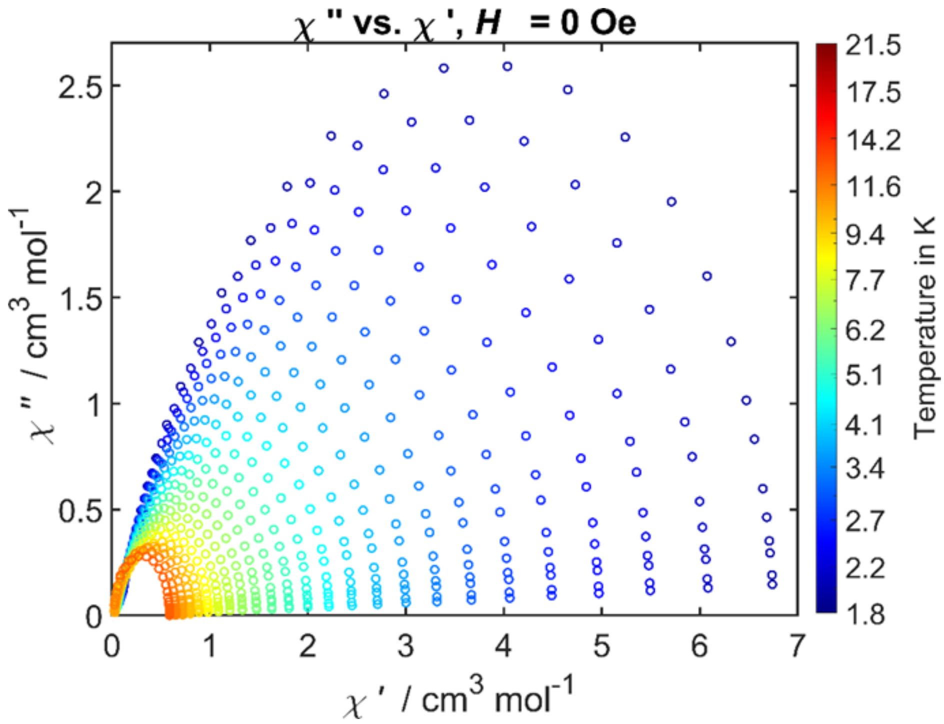

Figure S47: Cole-Cole plot of the in-phase and out-of-phase molar magnetic susceptibility of **3-Er** at different temperatures.

Table S9: Fitted parameters of Figure S45 and Figure S46 using the simultaneous fit with equations S1 and S2. Also listed are the errors calculated on basis of equation S4.

T / K	α	τ / s	χ_T / cm^3 mol^{-1}	χ_S / cm^3 mol^{-1}	τ lower limit / s	τ upper limit / s
1.8	0.161770	0.028824	6.888212	0.158997	0.008857	0.093802
2.0	0.161725	0.028762	6.215058	0.146657	0.008839	0.093580
2.2	0.161413	0.028656	5.616458	0.135302	0.008820	0.093099
2.5	0.161932	0.028478	5.081429	0.125296	0.008744	0.092748
2.7	0.161110	0.028298	4.590232	0.115665	0.008723	0.091804
3.0	0.161305	0.028040	4.149875	0.106962	0.008635	0.091050
3.4	0.161075	0.027767	3.751197	0.099012	0.008560	0.090066
3.7	0.161351	0.027540	3.392375	0.092041	0.008479	0.089446
4.1	0.161209	0.027350	3.066632	0.084489	0.008427	0.088769
4.6	0.161119	0.027242	2.771577	0.078466	0.008397	0.088380
5.1	0.161292	0.027131	2.504931	0.072212	0.008356	0.088092
5.6	0.160672	0.027002	2.262606	0.067157	0.008340	0.087417
6.2	0.160780	0.026880	2.044060	0.061694	0.008299	0.087068
6.9	0.160779	0.026737	1.847194	0.056697	0.008255	0.086604
7.7	0.160263	0.026579	1.669216	0.052680	0.0082257	0.085880
8.5	0.159757	0.026375	1.507925	0.048383	0.0081823	0.085018
9.4	0.158433	0.026080	1.361741	0.044708	0.0081417	0.083541
10.4	0.156818	0.025742	1.229931	0.041427	0.008098	0.081830
11.6	0.154046	0.025304	1.110219	0.038564	0.008066	0.079386
12.8	0.149392	0.024616	1.002277	0.036034	0.008022	0.075534
14.2	0.142980	0.023413	0.904022	0.033778	0.007868	0.069675
15.8	0.116847	0.019306	0.812783	0.031798	0.007367	0.050593
17.5	0.054772	0.009048	0.725723	0.028220	0.004836	0.016926
19.4	0.017731	0.002216	0.652348	0.021711	0.001568	0.003133
21.5	0.010437	0.000423	0.588543	0.016102	0.000325	0.000550

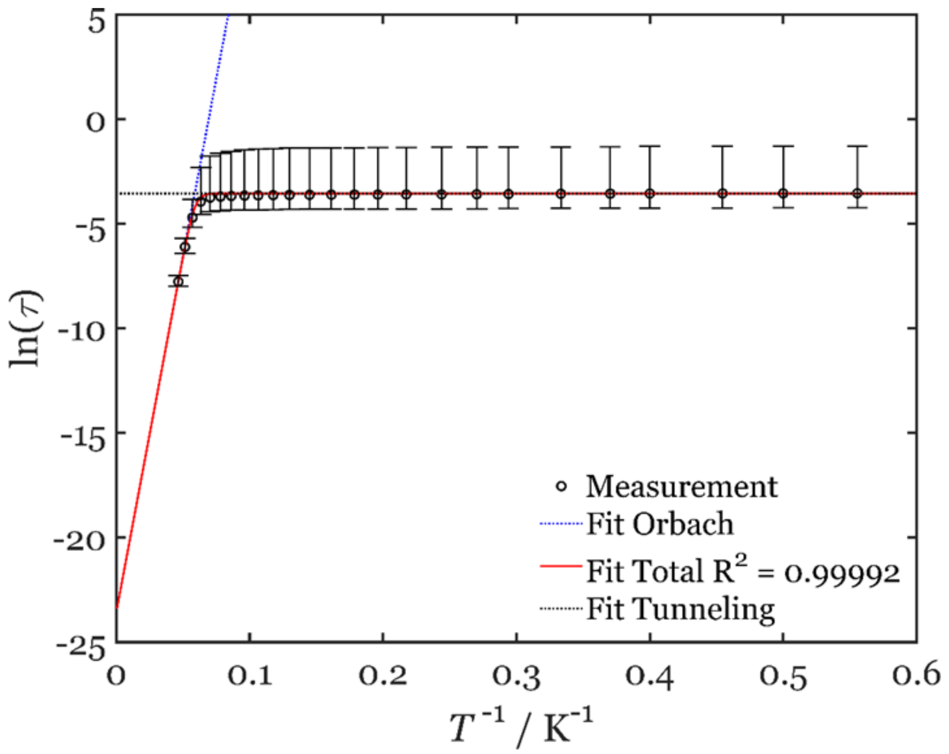

Figure S48: Arrhenius plot of **3-Er** using the data and uncertainties/ error bars from Table S9. The fit function is shown in equation 1 and the parameters in Table 1. In addition to the complete function (red line), the contributions from the Orbach process (blue dotted line) and the QTM (black dotted line) are shown.

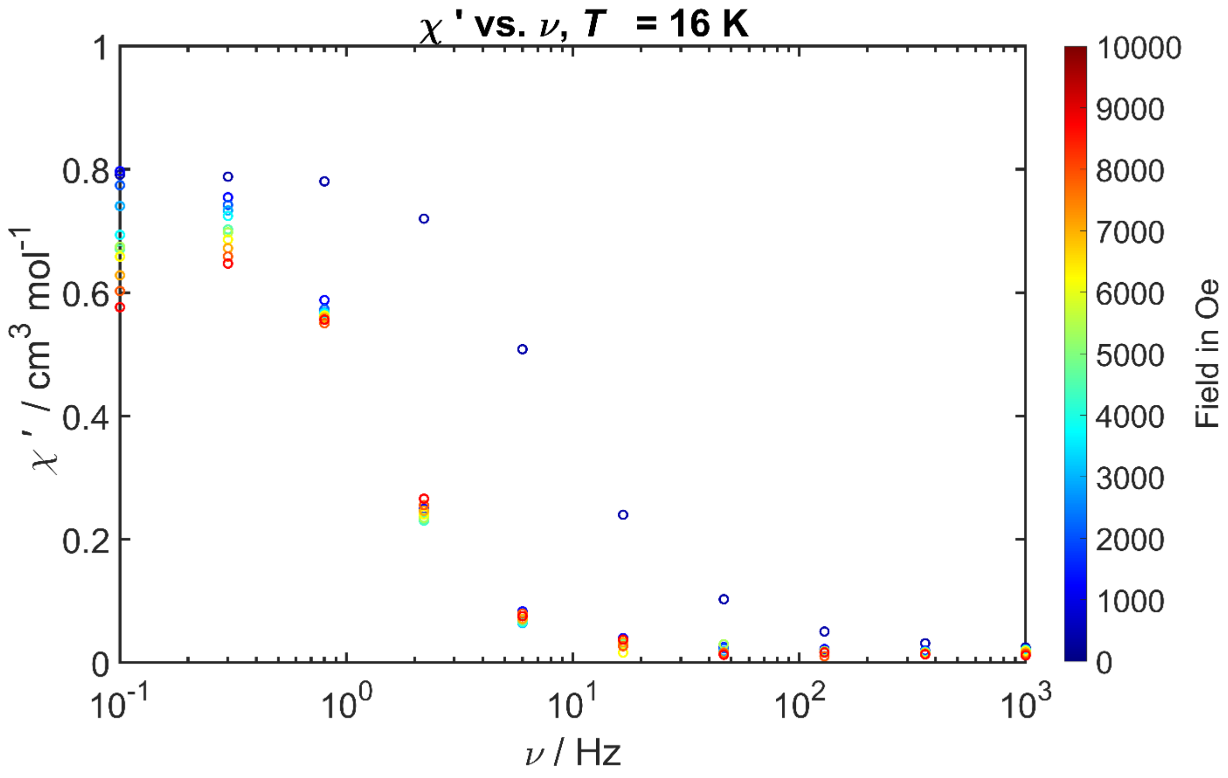

Figure S49: In-Phase molar magnetic susceptibility measurement at 16 K of **3-Er** in dependence on the frequency of an applied alternating field with a magnitude of 2 Oe at different applied static magnetic fields.

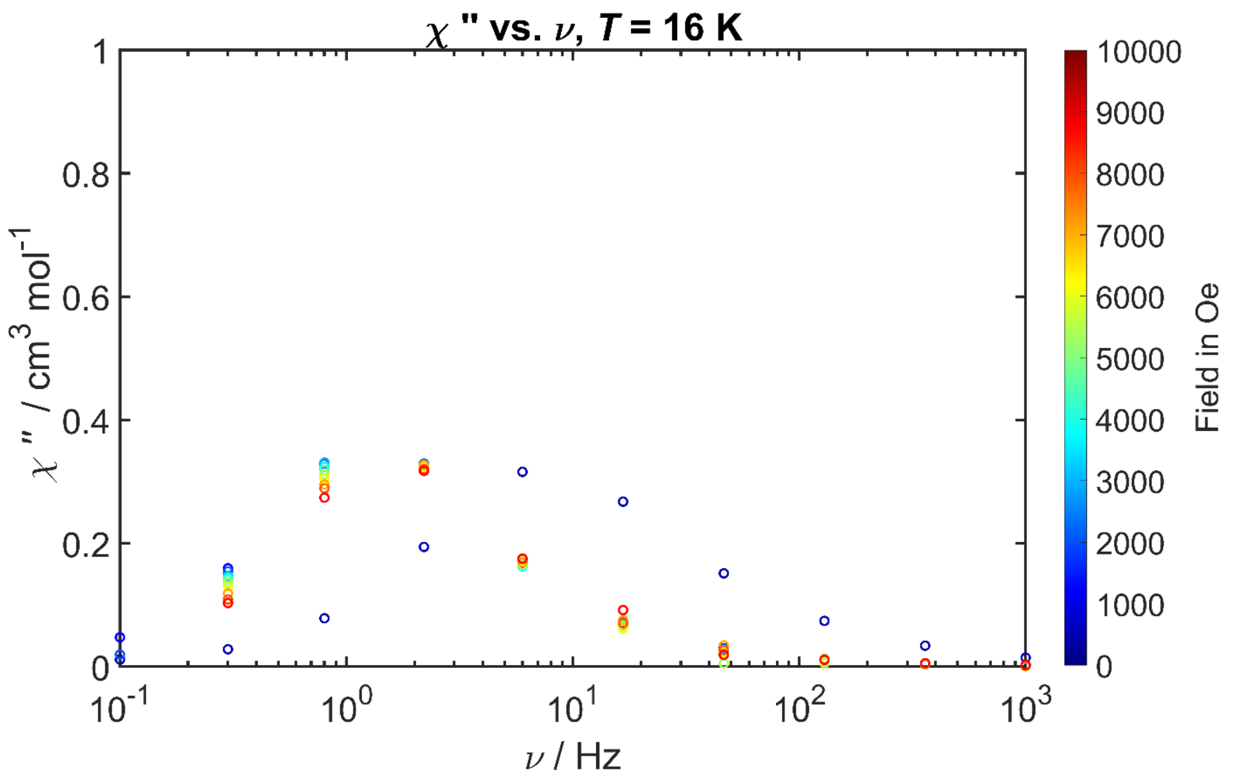

Figure S50: Out-of-Phase molar magnetic susceptibility measurement at 16 K of **3-Er** in dependence on the frequency of an applied alternating field with a magnitude of 2 Oe at different applied static magnetic fields.

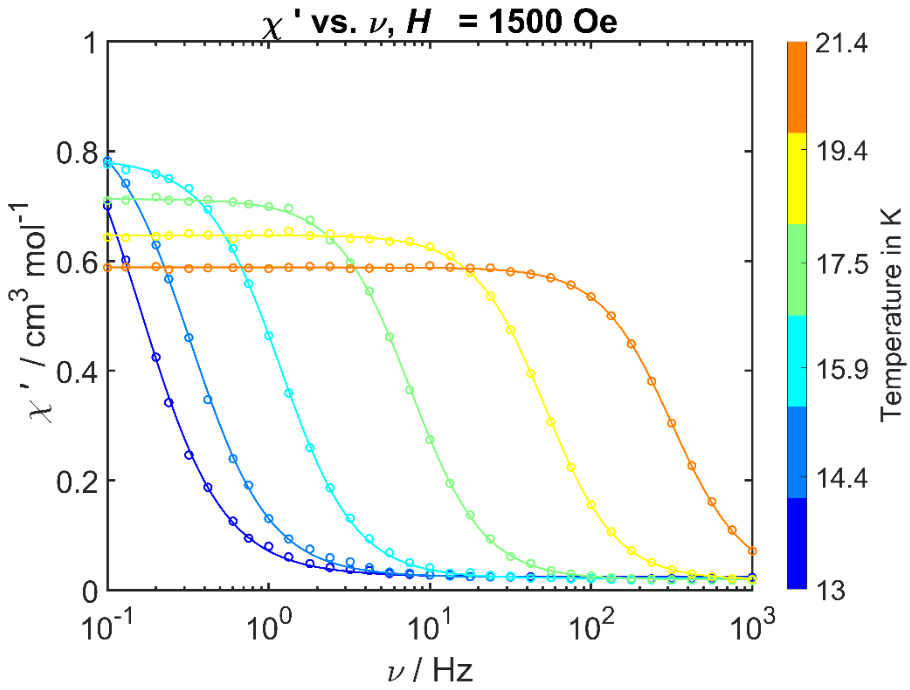

Figure S51: In-Phase molar magnetic susceptibility measurement of **3-Er** with an applied static magnetic field of 1500 Oe in dependence on the frequency of an applied alternating magnetic field with a magnitude of 2 Oe at different temperatures. The lines are fits according to Eq. S1. The resulting parameters are listed in Table S10.

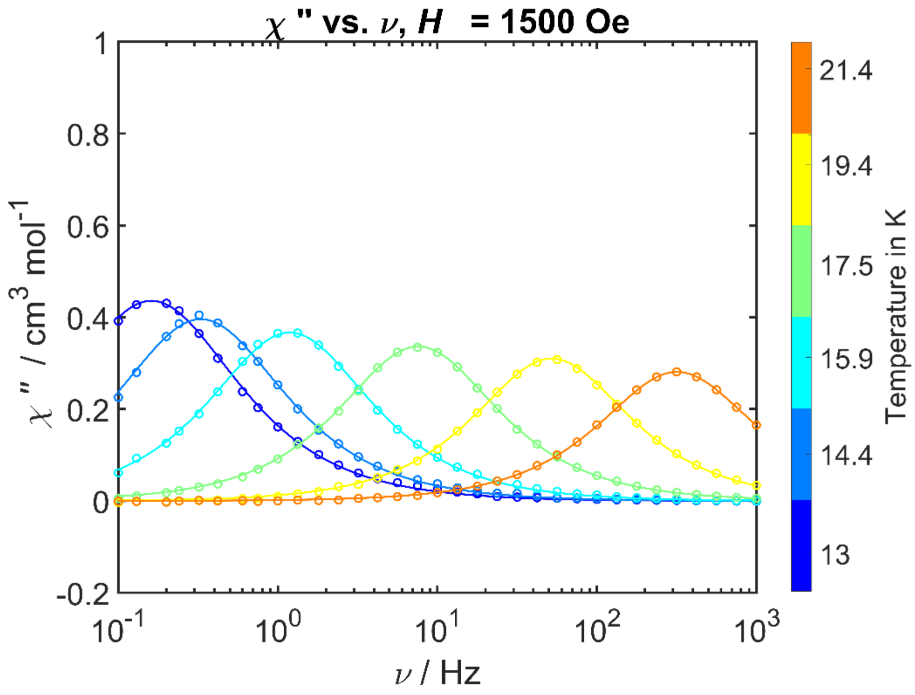

Figure S52: Out-of-Phase molar magnetic susceptibility measurement of **3-Er** with an applied static magnetic field of 1500 Oe in dependence on the frequency of an applied alternating magnetic field with a magnitude of 2 Oe at different temperatures. The lines are fits according to Eq. S2. The resulting parameters are listed in Table S10.

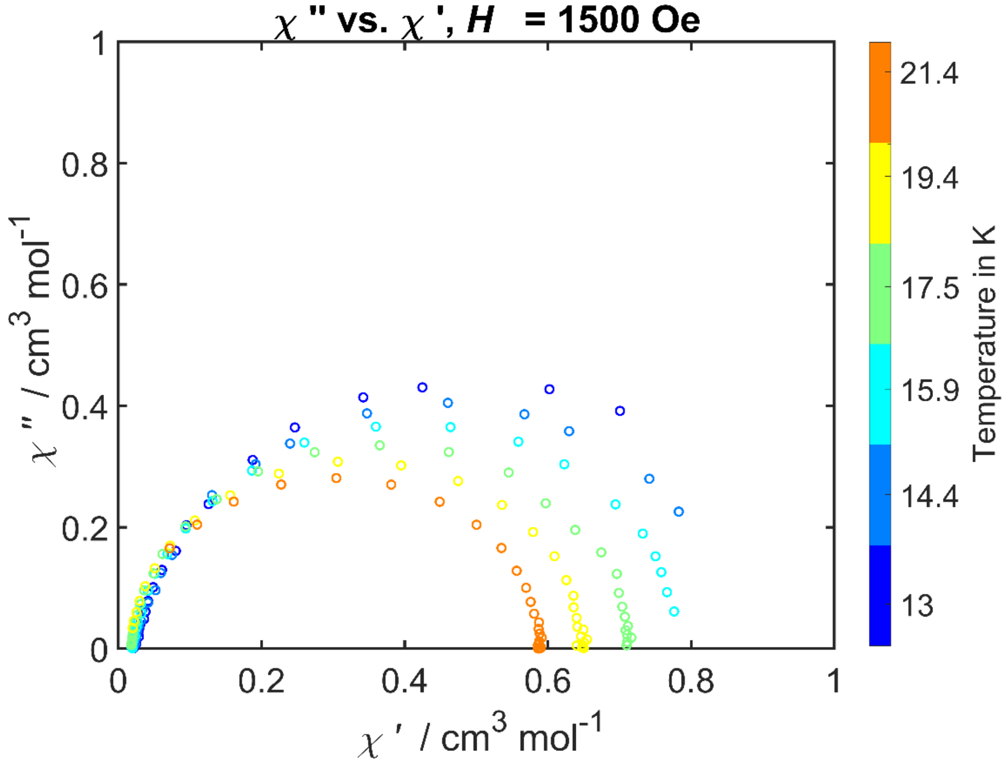

Figure S53: Cole-Cole plot of the in-phase and out-of-phase molar magnetic susceptibility of **3-Er** at different temperatures and an applied static magnetic field of 1500 Oe.

Table S10: Fitted parameters of Figure S51 and Figure S52 using the simultaneous fit with equations S1 and S2. Also listed are the errors calculated on basis of equation S4.

T / K	α	τ / s	χ_T / cm³ mol⁻¹	χ_S / cm³ mol⁻¹	τ lower limit / s	τ upper limit / s
13	0.0675729	0.9867297	0.9937579	0.0243114	0.4885618	1.9928606
14.4	0.0494393	0.4835859	0.8789125	0.0232894	0.2673820	0.8746113
15.9	0.0280841	0.1341284	0.7882084	0.0216528	0.0864551	0.2080899
17.5	0.0196431	0.0210052	0.7133033	0.0195643	0.0145831	0.0302555
19.4	0.0075738	0.0030639	0.6469536	0.0196847	0.0024477	0.0038351
21.4	0.0094975	0.0005014	0.5884378	0.0178532	0.0003898	0.0006450

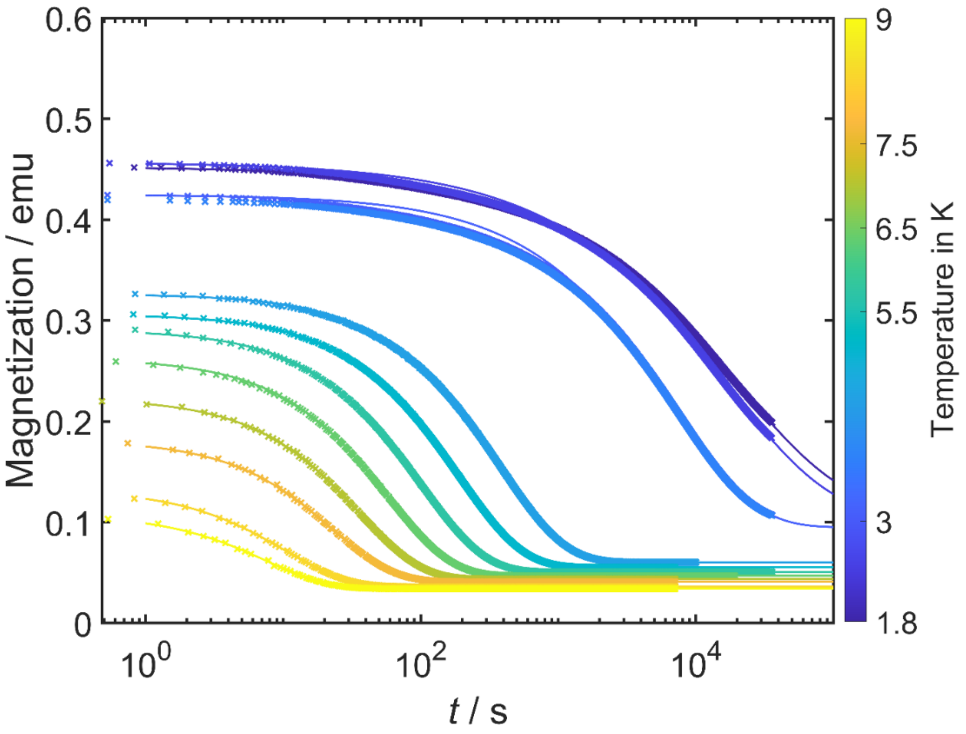

Figure S54: DC relaxation measurements of **3-Er** at different temperatures and a target field of 1500 Oe. Data was fitted using equation S5. Fit parameters are listed in Table S11. Converted relaxation times and their error are listed in Table S12.

Table S11: Fit parameters of the DC relaxation Data from Figure S54 fitted with equation S5.

T / K	M_0 / emu	τ* / s	M_{eq} / emu	β
1.79999949792210	0.452357941639477	19948.6241359335	0.112637193658222	0.560075873350577
2.00000077494384	0.456736778190710	17015.6096313900	0.106116676186506	0.576003030362311
3.00000201724192	0.424578244019164	6686.09407754056	0.0950000000024456	0.714332775702200
5.00000266451401	0.326328607650185	363.798021896483	0.0604034749292179	0.895234132083106
5.50000250547758	0.306216359827313	184.986238792638	0.0553950619728476	0.918711569570964
6.00000323195290	0.290962022879671	98.2444621997464	0.0508079349485685	0.919697402892581
6.50000370306936	0.262670927499597	56.7939439235440	0.0470522937312090	0.930992224292308
7.00000312107593	0.223127084240861	35.5606187357967	0.0437151039598597	0.959119860399352
7.50000360796500	0.181595415032722	23.1112918840774	0.0410556222162822	0.976040392506414
8.50000523091902	0.132966424871714	10.4415631681064	0.0361783452647269	0.960383700903029
9.00000368691651	0.108244001680721	7.66386474098708	0.0341845040037011	0.987768318651403

Table S12: Converted values from τ* using equation S6. Error limits were calculated using equation S4

T / K	τ / s	τ upper limit / s	τ lower limit / s
1.79999949792210	19347.3904710809	40947.1428962671	13139.1739523478
2.00000077494384	16979.8250773990	34049.8473757291	11329.8875841907
3.00000201724192	8100.94860820105	10128.5917751975	4501.00220404053
5.00000266451401	518.963625788220	285.827274065206	184.313662747750
5.50000250547758	268.269804826908	124.607414945640	85.0861419422725
6.00000323195290	142.571570772700	65.6961480254417	44.9728986890244
6.50000370306936	83.0488656333489	34.7065683522982	24.4773513554879
7.00000312107593	52.9538478325643	16.0530253924755	12.3186202208186
7.50000360796500	34.7763064735259	7.73978780944048	6.33080807257967
8.50000523091902	15.5610065594519	4.63039518118715	3.56852935288222
9.00000368691651	11.6133230744649	1.78158452047475	1.54462555819013

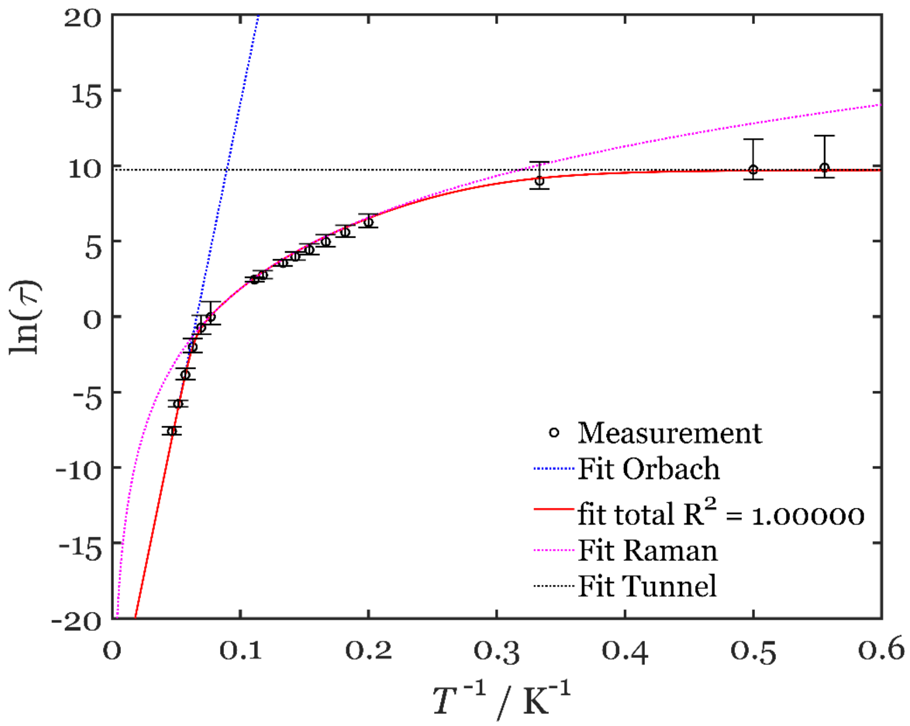

Figure S55: Arrhenius plot at 1500 Oe of **3-Er** using the data and uncertainties/ error bars from Table S10 and Table S12. The fit function is shown in equation 1 and the parameters in table 2. In addition to the complete function (red line), the contributions from the Orbach process (blue dotted line), Raman process (pink dotted line) and the QTM (black dotted line) are shown.

4-Er

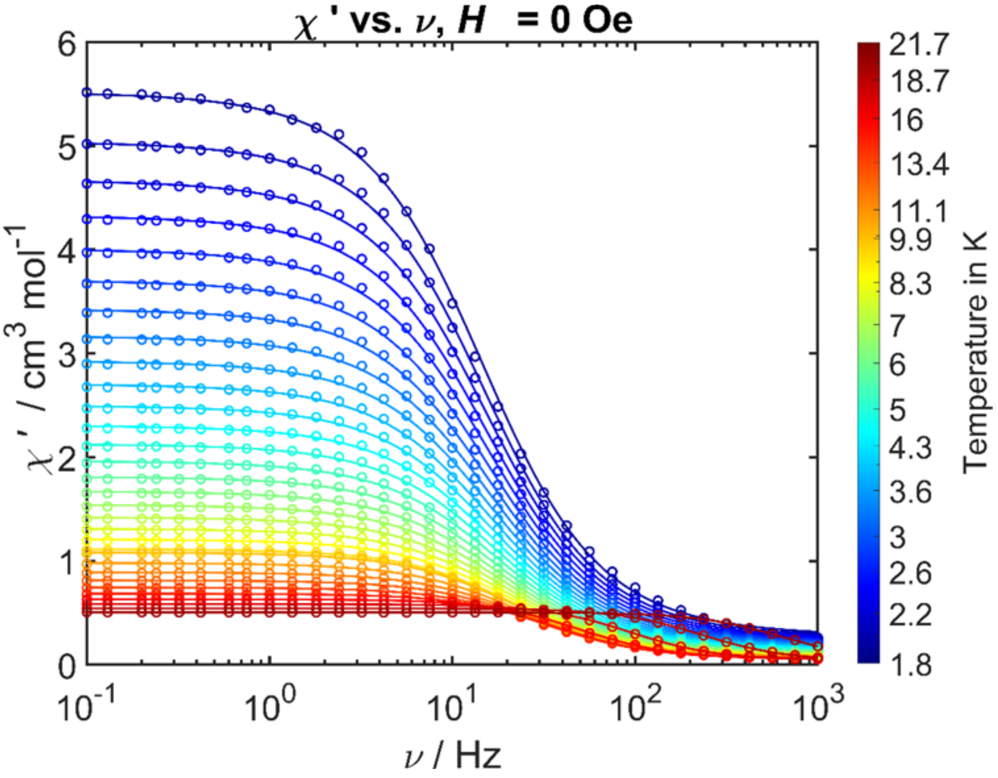

Figure S56: In-Phase molar magnetic susceptibility measurement of **4-Er** in dependence on the frequency of an applied alternating field with a magnitude of 2 Oe at different temperatures. The lines are fits according to Eq. S1. The resulting parameters are listed in Table S13.

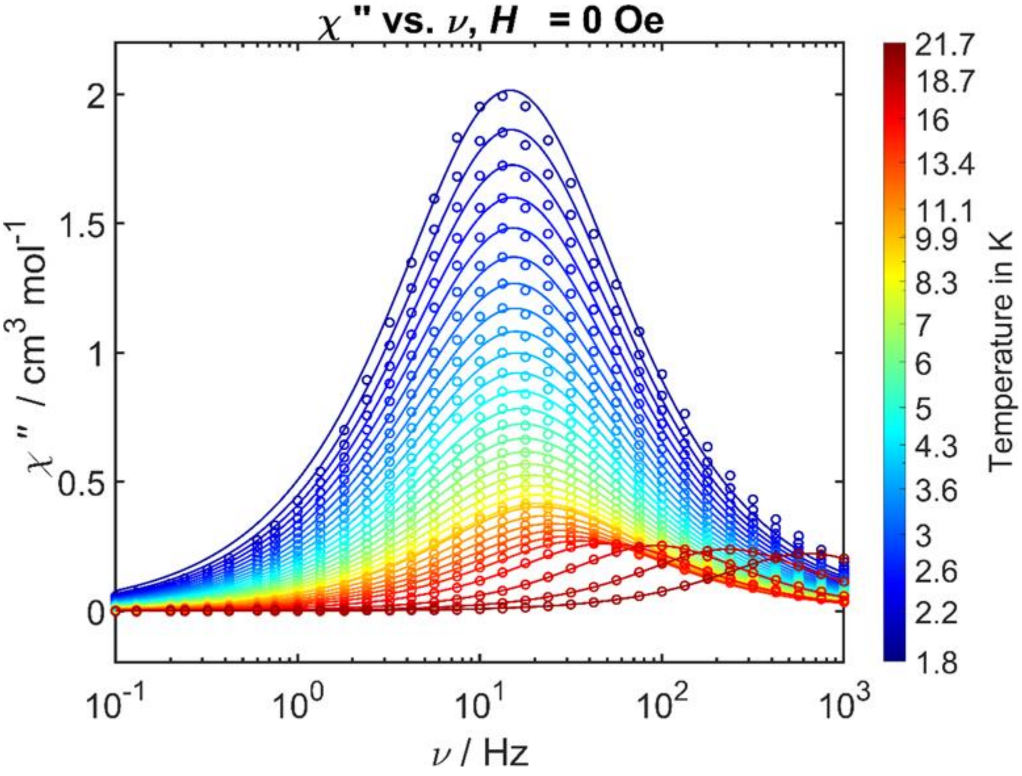

Figure S57: Out-of-Phase molar magnetic susceptibility measurement of **4-Er** in dependence on the frequency of an applied alternating field with a magnitude of 2 Oe at different temperatures. The lines are fits according to Eq. S2. The resulting parameters are listed in Table S13.

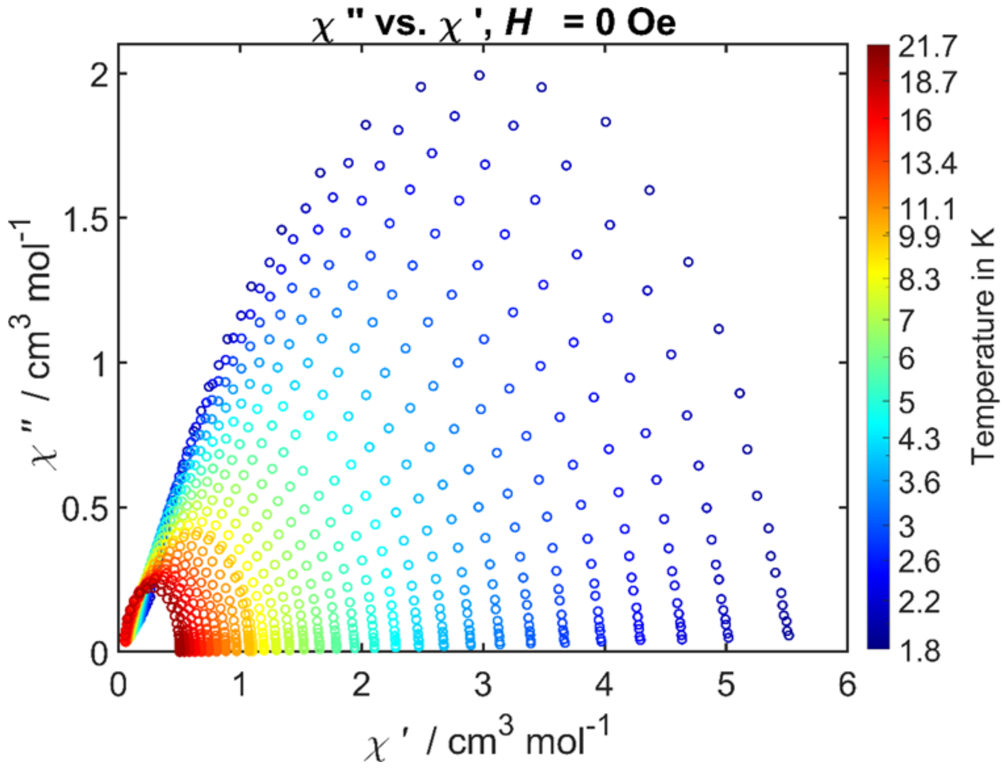

Figure S58: Cole-Cole plot of the in-phase and out-of-phase molar magnetic susceptibility of **4-Er** at different temperatures.

Table S13: Fitted parameters of Figure S56 and Figure S57 using the simultaneous fit with equations S1 and S2. Also listed are the errors calculated on basis of equation S4.

T / K	α	τ / s	χ_T / cm^3 mol^{-1}	χ_S / cm^3 mol^{-1}	τ lower limit / s	τ upper limit / s
1.8	0.16613995	0.01085757	5.52112604	0.27356686	0.00326811	0.03607180
2.0	0.15726014	0.01067095	5.04194960	0.26134164	0.00334985	0.03399230
2.2	0.15619978	0.01058185	4.66934520	0.24605170	0.00333864	0.03353929
2.4	0.15560555	0.01053475	4.32845175	0.23225318	0.00333317	0.03329594
2.6	0.15442855	0.01045553	4.00667353	0.21910148	0.00332665	0.03286131
2.8	0.15419541	0.01038409	3.70485517	0.20561466	0.00330758	0.03260065
3.0	0.15316527	0.01029605	3.42653702	0.19402141	0.00329564	0.03216635
3.3	0.15312396	0.01018957	3.16782516	0.18184119	0.00326220	0.03182741
3.6	0.15272484	0.01006915	2.92734925	0.17060934	0.00322977	0.03139165
3.9	0.15284095	0.00993969	2.70447950	0.16012581	0.00318649	0.03100516
4.3	0.15255470	0.00979216	2.49746613	0.15024352	0.00314347	0.03050340
4.6	0.15208387	0.00962590	2.30526875	0.14097837	0.00309702	0.02991839
5.0	0.15302589	0.00942919	2.12756321	0.13131901	0.00302017	0.02943861
5.5	0.15147408	0.00927012	1.96422065	0.12390853	0.00299123	0.02872908
6.0	0.15104283	0.00907068	1.81262740	0.11601005	0.00293289	0.02805331
6.5	0.15021286	0.00885666	1.67213135	0.10837390	0.00287503	0.02728333
7.0	0.14861740	0.00863038	1.54236917	0.10219447	0.00282297	0.02638480
7.7	0.14718791	0.00837316	1.42259585	0.09559985	0.00275758	0.02542435
8.3	0.14533187	0.00808746	1.31180135	0.08930611	0.00268722	0.02433999
9.1	0.14249998	0.00777144	1.20938386	0.08403341	0.00261748	0.02307389
9.9	0.13868243	0.00741948	1.11457218	0.07876082	0.00254518	0.02162865
10.0	0.14053867	0.00735389	1.08527996	0.07688062	0.00250026	0.02162961
11.1	0.13436187	0.00687362	0.98238016	0.07156529	0.00240758	0.01962416
12.2	0.12708522	0.00635775	0.89271631	0.06666815	0.00230698	0.01752114
13.4	0.11912679	0.00580749	0.81504701	0.06254781	0.00219125	0.01539165
14.7	0.11049185	0.00520103	0.74622218	0.05853932	0.00204859	0.01320456
16.0	0.09388636	0.00444199	0.68614322	0.05533502	0.00190465	0.01035952
17.3	0.06590287	0.00319673	0.63204778	0.05135587	0.00159821	0.00639411
18.7	0.03949867	0.00170002	0.58418015	0.04602789	0.00100516	0.00287522
20.2	0.03277154	0.00069353	0.54248238	0.03911390	0.00043080	0.00111648
21.7	0.03176220	0.00025254	0.50496446	0.03742904	0.00015809	0.00040341

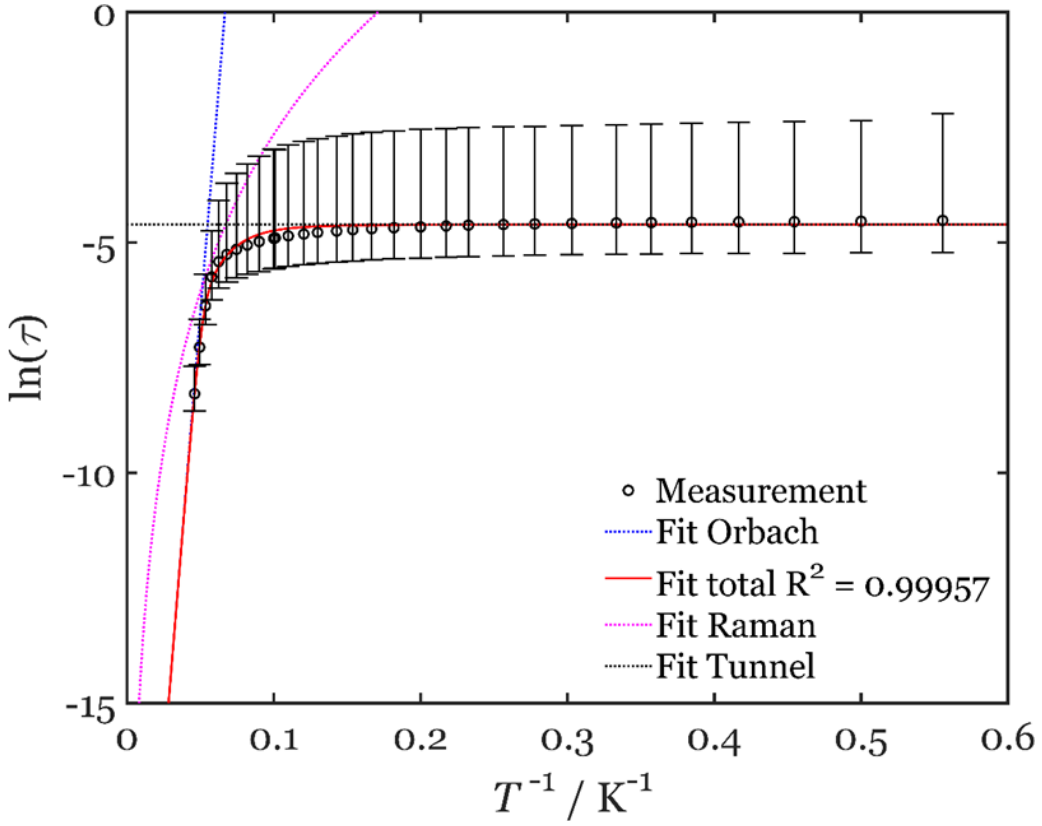

Figure S59: Arrhenius plot of **4-Er** using the data and uncertainties/ error bars from Table S13. The fit function is shown in equation 1 and the parameters in Table 1. In addition to the complete function (red line), the contributions from the Orbach process (blue dotted line), The Raman process (pink dotted line) and the QTM (black dotted line) are shown.

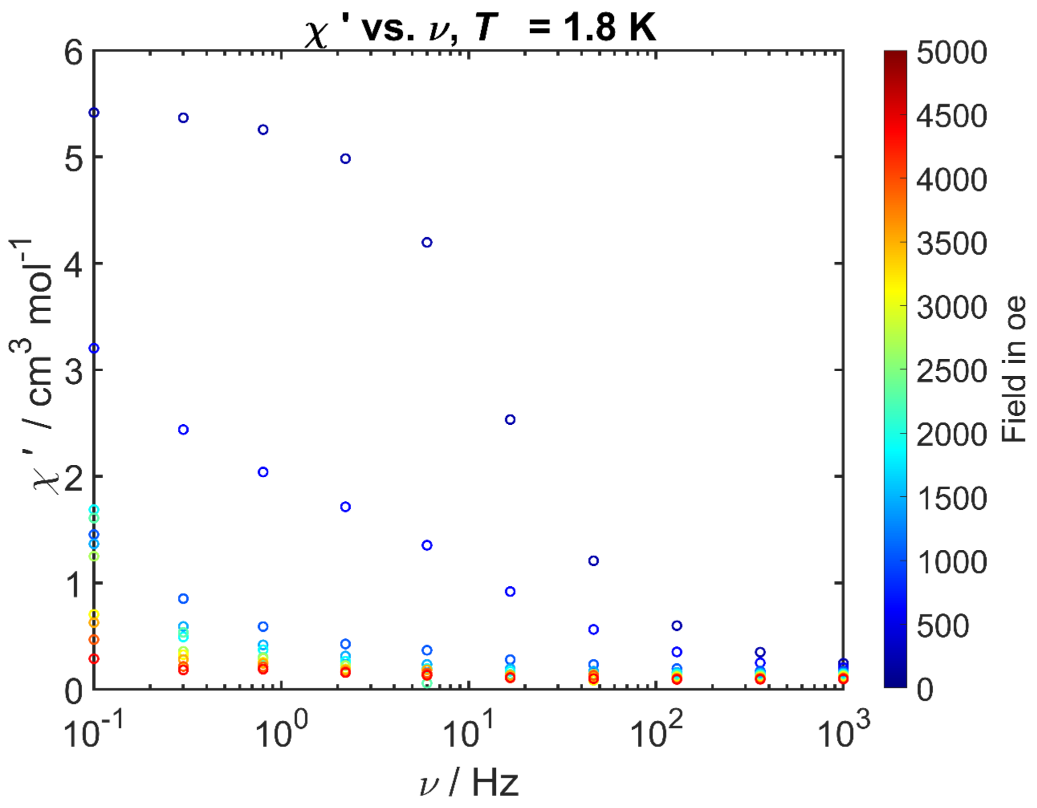

Figure S60: In-Phase molar magnetic susceptibility measurement at 1.8 K of **4-Er** in dependence on the frequency of an applied alternating field with a magnitude of 2 Oe at different applied static magnetic fields.

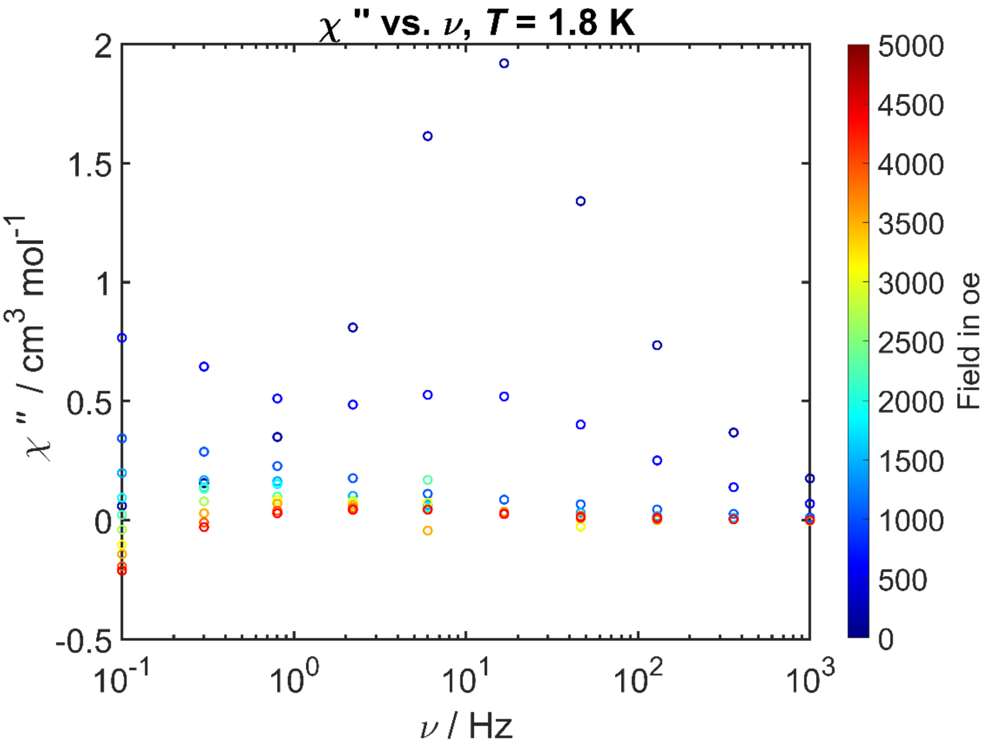

Figure S61: Out-of-Phase molar magnetic susceptibility measurement at 1.8 K of **4-Er** in dependence on the frequency of an applied alternating field with a magnitude of 2 Oe at different applied static magnetic fields.

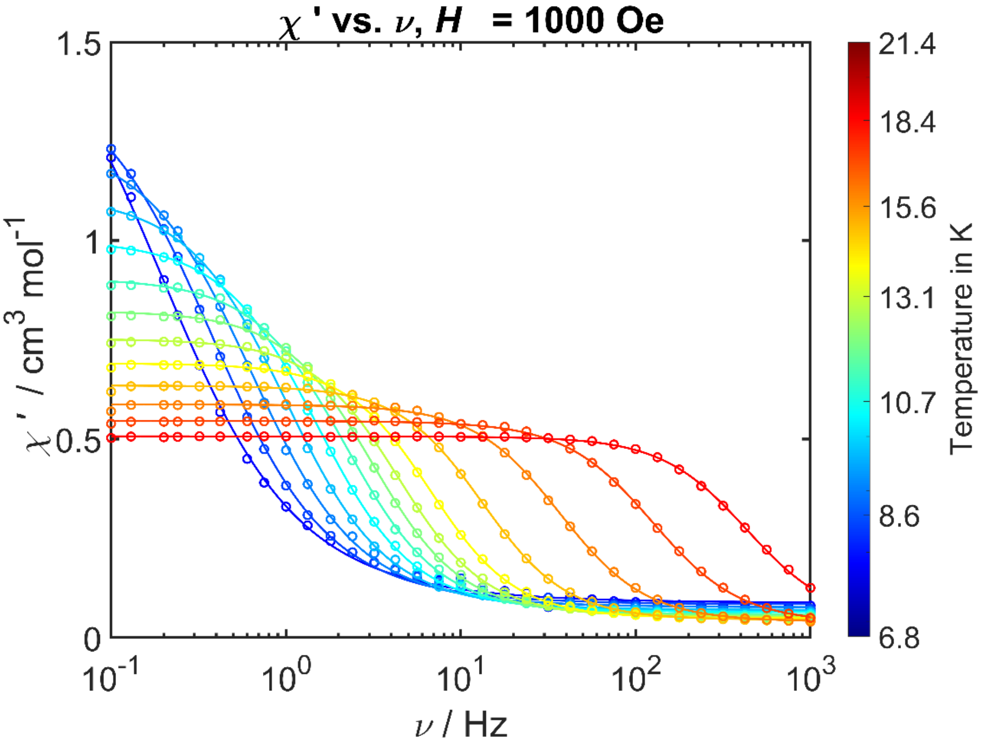

Figure S62: In-Phase molar magnetic susceptibility measurement of **4-Er** with an applied static magnetic field of 1000 Oe in dependence on the frequency of an applied alternating magnetic field with a magnitude of 2 Oe at different temperatures. The lines are fits according to Eq. S1. The resulting parameters are listed in Table S14.

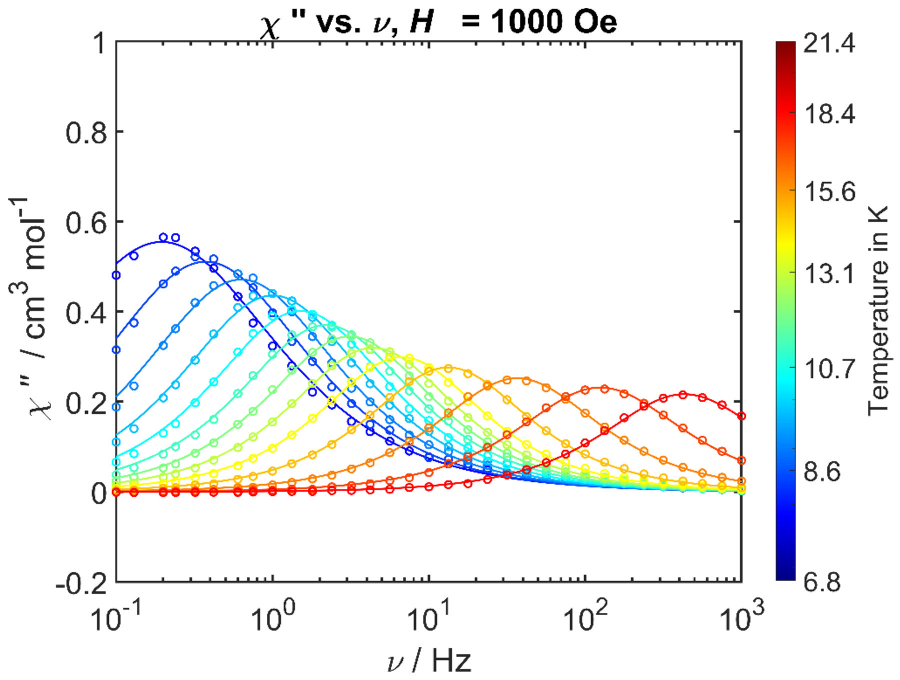

Figure S63: Out-of-Phase molar magnetic susceptibility measurement of **4-Er** with an applied static magnetic field of 1000 Oe in dependence on the frequency of an applied alternating magnetic field with a magnitude of 2 Oe at different temperatures. The lines are fits according to Eq. S2. The resulting parameters are listed in Table S14.

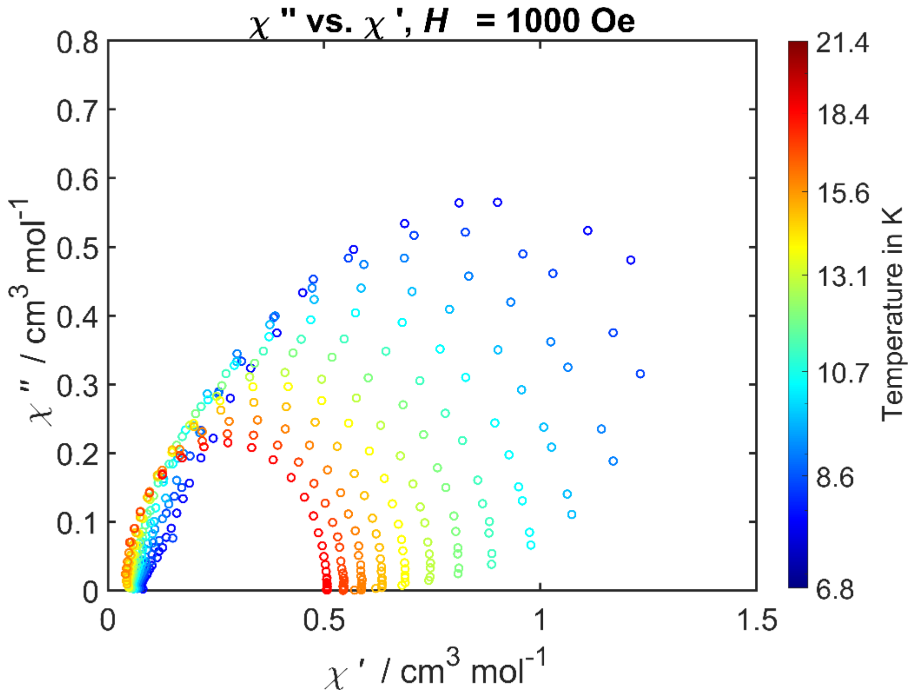

Figure S64: Cole-Cole plot of the in-phase and out-of-phase molar magnetic susceptibility of **4-Er** at different temperatures and an applied static magnetic field of 1000 Oe.

Table S14: Fitted parameters of Figure S62 and Figure S63 using the simultaneous fit with equations S1 and S2. Also listed are the errors calculated on basis of equation S4.

T / K	α	τ / s	χ_T / cm^3 mol^{-1}	χ_S / cm^3 mol^{-1}	τ lower limit / s	τ upper limit / s
6.8	0.242601	0.812807	1.727550	0.088529	0.170174	3.882237
7.7	0.180846	0.425540	1.446055	0.084090	0.119502	1.515325
8.6	0.146341	0.252334	1.264738	0.076855	0.083439	0.763096
9.7	0.117017	0.158328	1.118109	0.069957	0.060366	0.415261
10.7	0.100306	0.105217	1.004558	0.064697	0.043641	0.253676
11.9	0.086633	0.072353	0.906777	0.059095	0.032235	0.162402
13.1	0.068299	0.051158	0.823997	0.056183	0.025224	0.103755
14.3	0.058804	0.036164	0.752912	0.052167	0.018858	0.069350
15.6	0.045875	0.023728	0.691211	0.048954	0.013430	0.041923
17	0.042628	0.011988	0.635253	0.044909	0.006936	0.020722
18.4	0.053313	0.004328	0.587786	0.039824	0.002335	0.008023
19.9	6.17E-02	1.28E-03	5.46E-01	3.62E-02	6.54E-04	2.49E-03
21.4	4.91E-02	3.70E-04	5.07E-01	3.92E-02	2.05E-04	6.68E-04

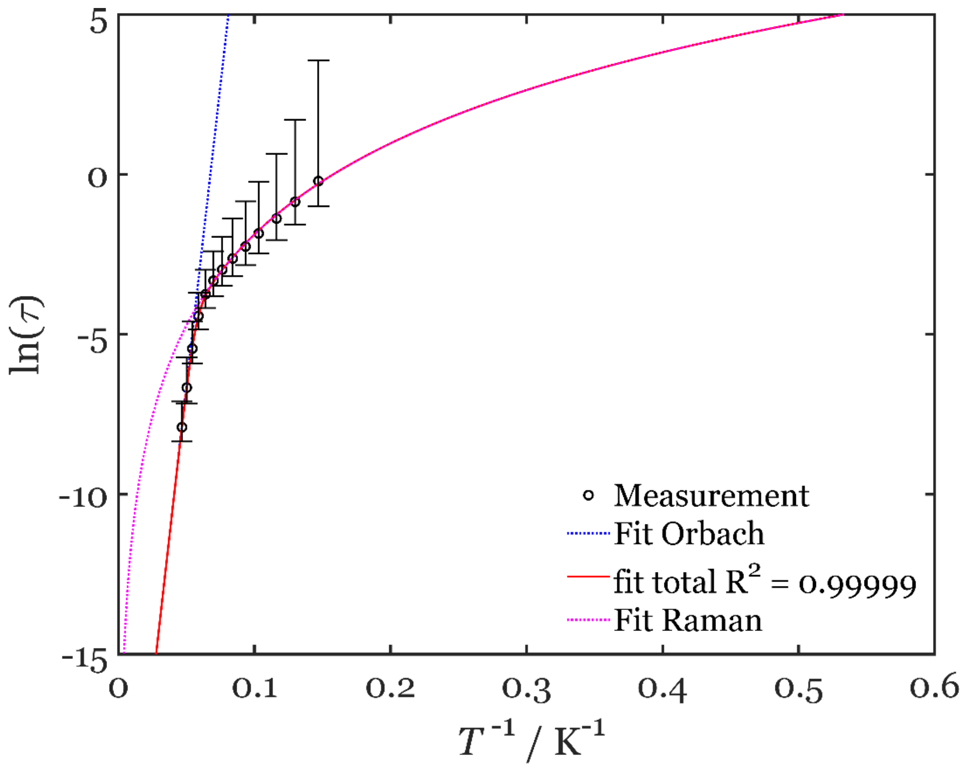

Figure S65: Arrhenius plot at 1000 Oe of **4-Er** using the data and uncertainties/ error bars from Table S14. The fit function is shown in equation 1 and the parameters in Table 2. In addition to the complete function (red line), the contributions from the Orbach process (blue dotted line) and Raman process (pink dotted line) are shown.

5-Er

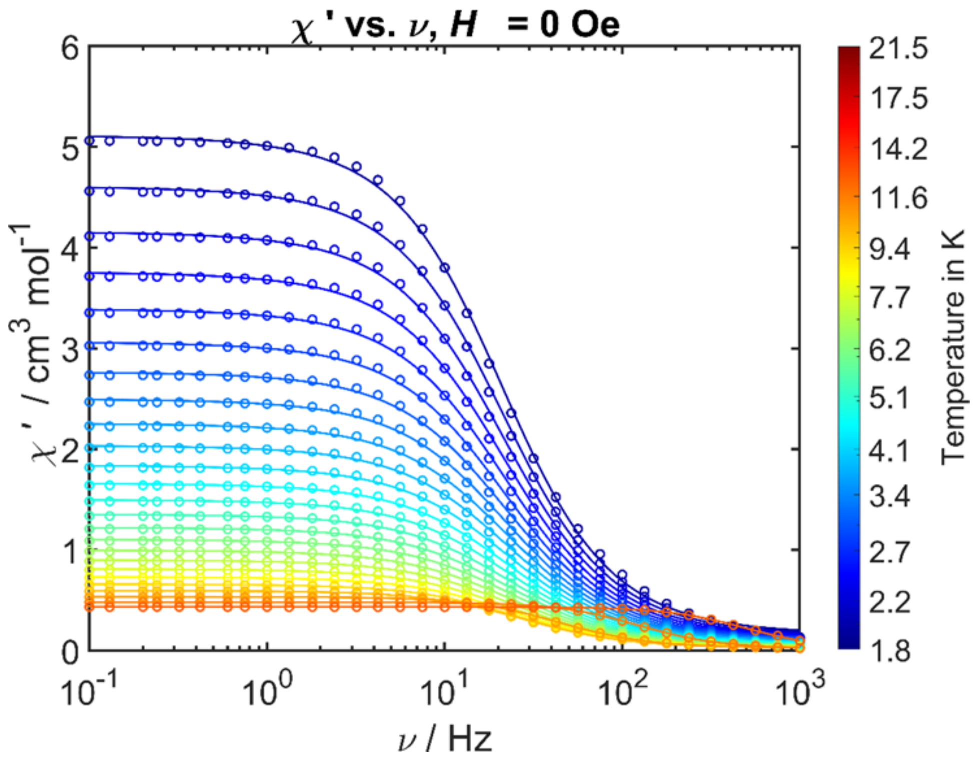

Figure S66: In-Phase molar magnetic susceptibility measurement of **5-Er** in dependence on the frequency of an applied alternating field with a magnitude of 2 Oe at different temperatures. The lines are fits according to Eq. S1. The resulting parameters are listed in Table S15.

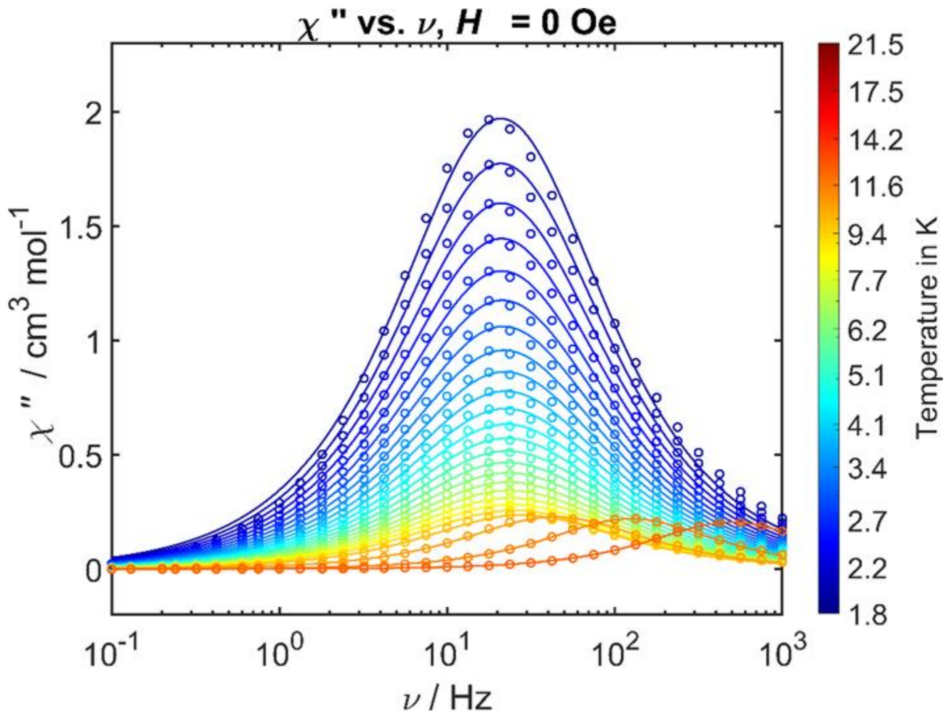

Figure S67: Out-of-Phase molar magnetic susceptibility measurement of **5-Er** in dependence on the frequency of an applied alternating field with a magnitude of 2 Oe at different temperatures. The lines are fits according to Eq. S2. The resulting parameters are listed in Table S15.

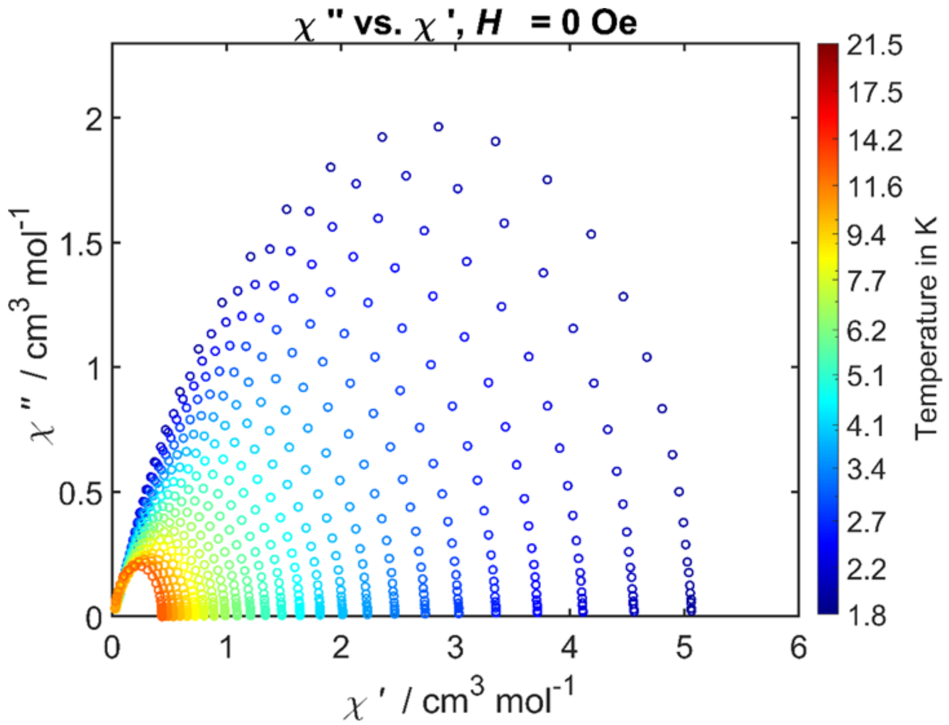

Figure S68: Cole-Cole plot of the in-phase and out-of-phase molar magnetic susceptibility of **5-Er** at different temperatures.

Table S15: Fitted parameters of Figure S66 and Figure S67 using the simultaneous fit with equations S1 and S2. Also listed are the errors calculated on basis of equation S4.

T / K	α	τ / s	χ_T / cm^3 mol^{-1}	χ_S / cm^3 mol^{-1}	τ lower limit / s	τ upper limit / s
1.8	0.1449808	0.0076044	5.1140817	0.1569968	0.0025310	0.0228477
2.0	0.1451247	0.0075864	4.6064756	0.1431774	0.0025232	0.0228095
2.2	0.1450840	0.0075682	4.1575828	0.1314953	0.0025177	0.0227503
2.5	0.1448617	0.0075424	3.7568715	0.1211588	0.0025118	0.0226487
2.7	0.1448151	0.0075081	3.3914249	0.1122337	0.0025009	0.0225406
3.0	0.1452810	0.0074502	3.0624914	0.1033595	0.0024761	0.0224168
3.4	0.1452626	0.0073739	2.7655611	0.0954053	0.0024509	0.0221851
3.7	0.1453862	0.0073002	2.4970717	0.0883911	0.0024250	0.0219763
4.1	0.1460833	0.0072207	2.2556225	0.0814918	0.0023906	0.0218097
4.6	0.1463642	0.0071578	2.0364309	0.0750694	0.0023666	0.0216487
5.1	0.1464680	0.0071042	1.8388645	0.0692240	0.0023477	0.0214973
5.6	0.1466655	0.0070705	1.6613898	0.0635620	0.0023344	0.0214155
6.2	0.1463607	0.0070336	1.5000605	0.0587787	0.0023256	0.0212728
6.9	0.1463164	0.0069994	1.3550800	0.0541515	0.0023148	0.0211648
7.7	0.1459212	0.0069537	1.2236784	0.0501115	0.0023040	0.0209870
8.5	0.1449346	0.0068987	1.1049230	0.0466739	0.0022966	0.0207229
9.4	0.1438114	0.0068403	0.9981365	0.0432162	0.0022894	0.0204371
10.4	0.1419214	0.0067741	0.9011011	0.0403363	0.0022879	0.0200568
11.6	0.1403080	0.0066801	0.8130868	0.0373913	0.0022737	0.0196261
12.8	0.1363726	0.0065563	0.7337431	0.0348405	0.0022742	0.0189008
14.2	0.1306916	0.0063399	0.6623761	0.0323512	0.0022605	0.0177813
15.8	0.1146765	0.0056614	0.5969491	0.0300295	0.0021838	0.0146769
17.5	0.0713145	0.0036020	0.5367748	0.0268647	0.0017457	0.0074323
19.4	0.0298268	0.0012789	0.4826935	0.0214111	0.0008128	0.0020121
21.5	0.0151086	0.0003222	0.4356780	0.0168862	0.0002342	0.0004433

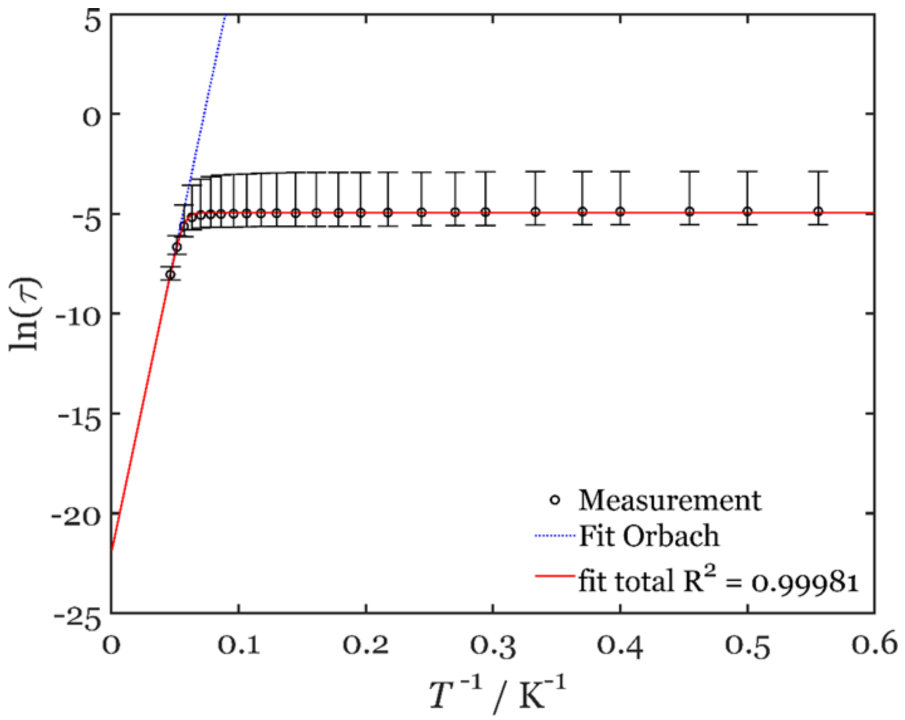

Figure S69: Arrhenius plot of **5-Er** using the data and uncertainties/ error bars from Table S15. The fit function is shown in equation 1 and the parameters in Table 1. In addition to the complete function (red line), the contributions from the Orbach process (blue dotted line) and the QTM (black dotted line) are shown.

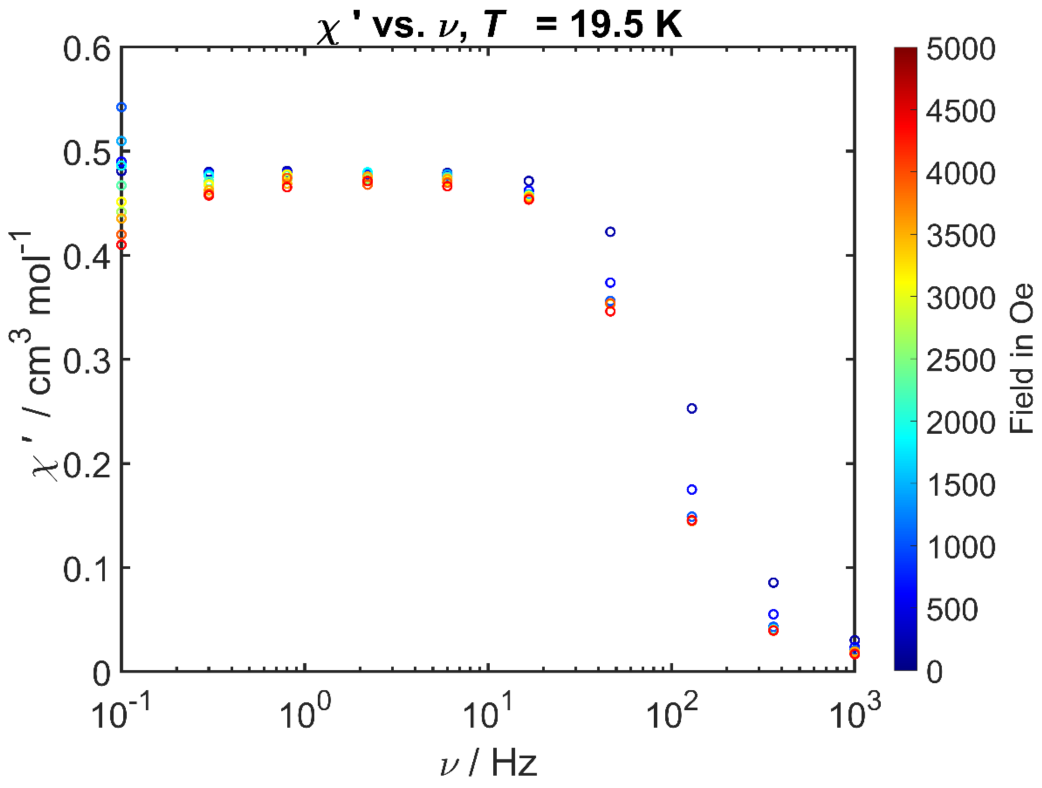

Figure S70: In-Phase molar magnetic susceptibility measurement at 19.5 K of **5-Er** in dependence on the frequency of an applied alternating field with a magnitude of 2 Oe at different applied static magnetic fields.

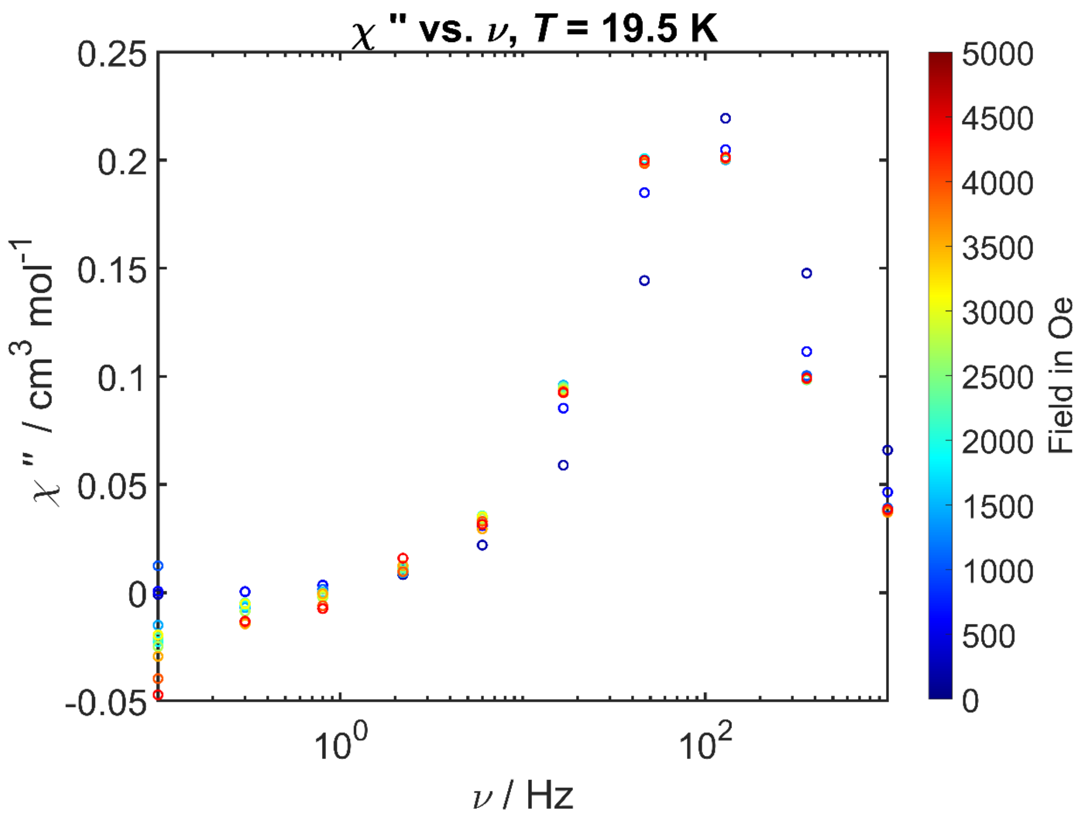

Figure S71: Out-of-Phase molar magnetic susceptibility measurement at 19.5 K of **5-Er** in dependence on the frequency of an applied alternating field with a magnitude of 2 Oe at different applied static magnetic fields.

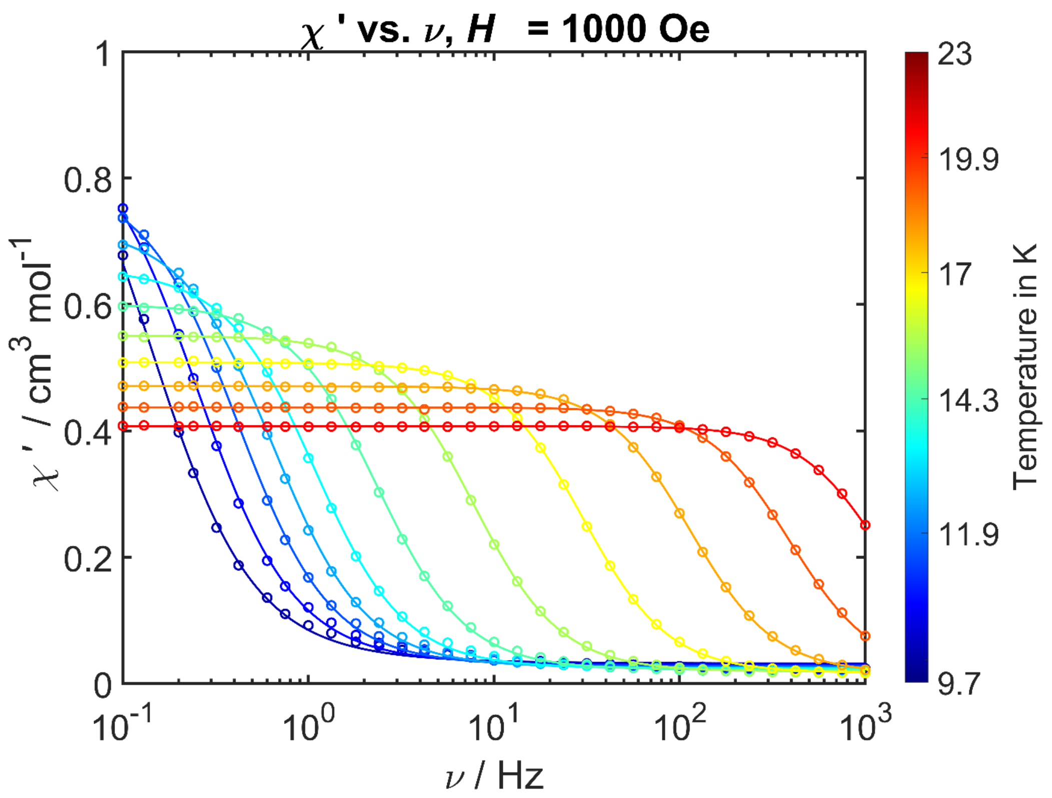

Figure S72: In-Phase molar magnetic susceptibility measurement of **5-Er** with an applied static magnetic field of 1000 Oe in dependence on the frequency of an applied alternating magnetic field with a magnitude of 2 Oe at different temperatures. The lines are fits according to Eq. S1. The resulting parameters are listed in Table S16.

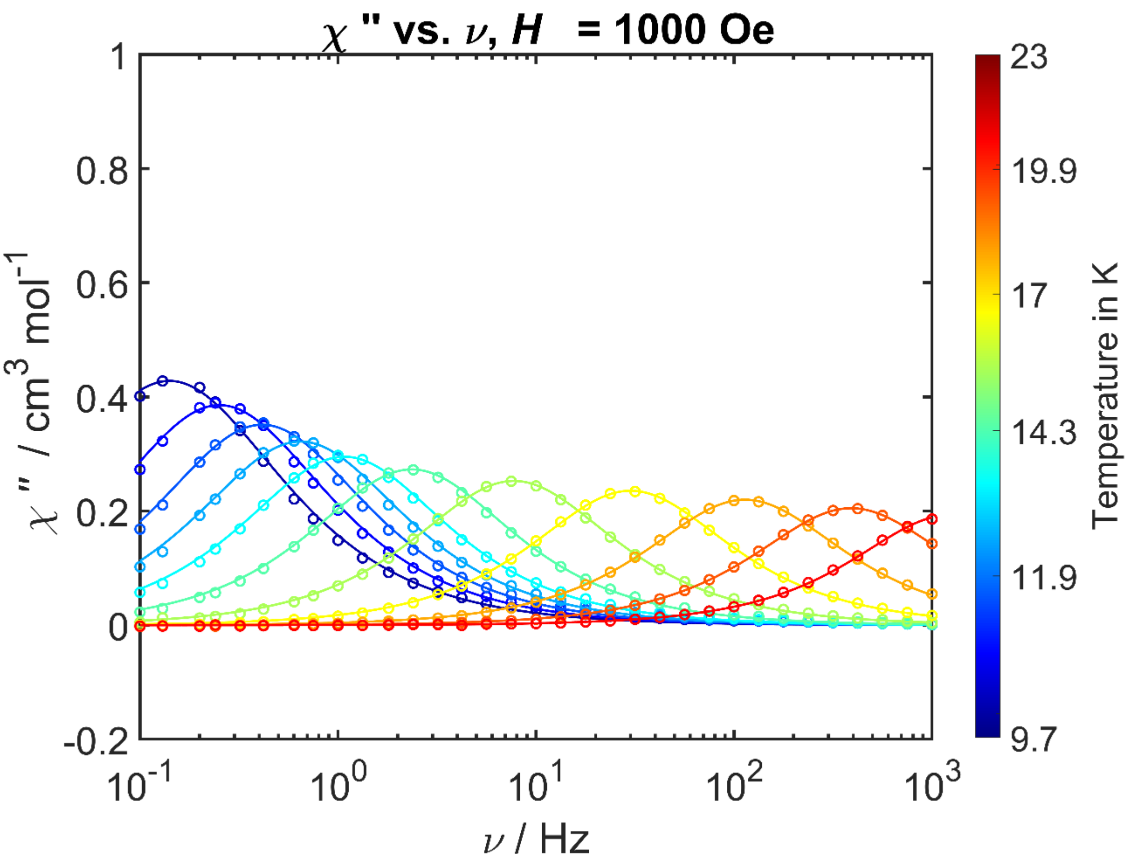

Figure S73: Out-of-Phase molar magnetic susceptibility measurement of **5-Er** with an applied static magnetic field of 1000 Oe in dependence on the frequency of an applied alternating magnetic field with a magnitude of 2 Oe at different temperatures. The lines are fits according to Eq. S2. The resulting parameters are listed in Table S16.

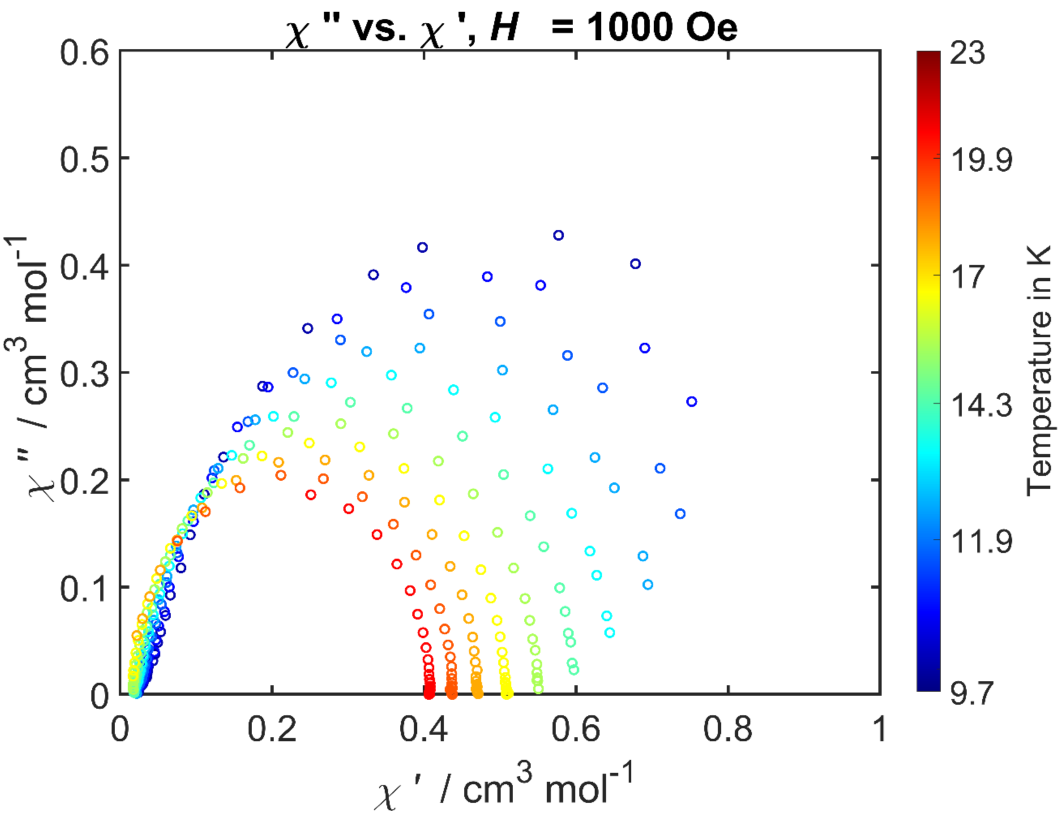

Figure S74: Cole-Cole plot of the in-phase and out-of-phase molar magnetic susceptibility of **5-Er** at different temperatures and an applied static magnetic field of 1000 Oe.

Table S16: Fitted parameters of Figure S72 and Figure S73 using the simultaneous fit with equations S1 and S2. Also listed are the errors calculated on basis of equation S4.

T / K	α	τ / s	χ_T / cm³ mol⁻¹	χ_S / cm³ mol⁻¹	τ lower limit / s	τ upper limit / s
9.7	0.102038	1.129577	1.037817	0.032125	0.464375	2.747659
10.7	0.077192	0.631236	0.901420	0.030485	0.296025	1.346035
11.9	0.060813	0.384491	0.801210	0.028033	0.198090	0.746295
13.1	0.053692	0.245914	0.725576	0.025364	0.132340	0.456957
14.3	0.044467	0.147778	0.657288	0.024060	0.084433	0.258646
15.6	0.037752	0.067523	0.601027	0.021711	0.040424	0.112787
17	0.032707	0.020601	0.551318	0.019053	0.012803	0.033148
18.4	0.027060	0.005303	0.508615	0.017048	0.003447	0.008158
19.9	0.021107	0.001403	0.470814	0.016290	0.000961	0.002049
21.4	0.017646	0.000408	0.437415	0.015893	0.000289	0.000576
23	0.008962	0.000130	0.407587	0.017157	0.000102	0.000166

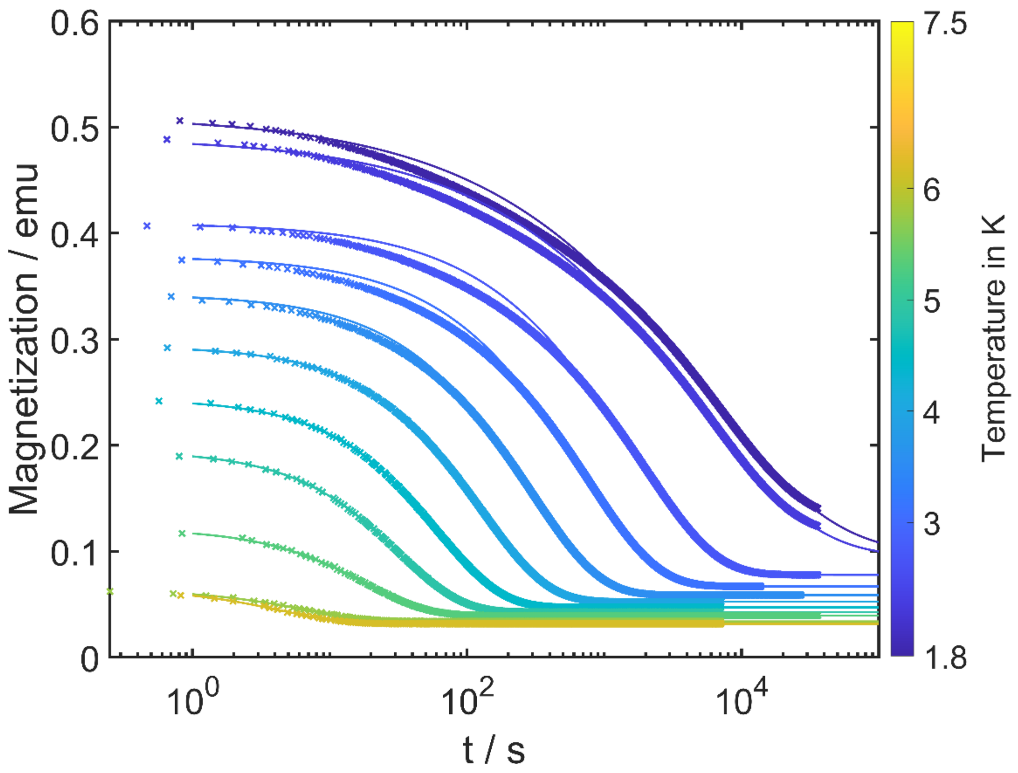

Figure S75: DC relaxation measurements of **5-Er** at different temperatures and a target field of 1000 Oe. Data was fitted using equation S5. Fit parameters are listed in Table S17. Converted relaxation times and their error are listed in Table S18.

Table S17: Fit parameters of the DC relaxation Data from Figure S75 fitted with equation S5.

T / K	M_0 / emu	τ^* / s	M_{eq} / emu	β
1.8	0.510849202878839	5424.60596201979	0.0994221485022287	0.459904251778751
2	0.490032636358513	4618.38432043614	0.0959293483966738	0.495529892666388
3	0.409355287025262	1476.64300238168	0.0778995154171395	0.696657850515263
3.5	0.378116979694508	625.659056610404	0.0670251493771512	0.752716652159222
4	0.342484055136278	264.472901040500	0.0588437758445248	0.809186462421649
4.5	0.293591005141085	123.303995575253	0.0525159067863463	0.882771057234756
5	0.243808823654742	60.7891372030584	0.0473263948298043	0.927543480529726
5.5	0.195228825086618	31.7955636349486	0.0429041871784526	0.947389264070046
6	0.121675615456993	18.3316230615335	0.0395643000493290	0.969186736067702
7	0.0644766579137428	6.57953653260986	0.0339579416679871	0.932125605755122
7.5	0.0656922397627843	4.42498760805860	0.0317813229738691	0.947925175909760

Table S18: Converted values from τ^* using equation S6. Error limits were calculated using equation S4.

T / K	τ / s	τ upper limit / s	τ lower limit / s
1.8	4203.28918419048	12670.1865474682	3156.22334862087
2	3916.50483728096	10369.3153596231	2842.78208779757
3	1752.81234583778	2333.05592182809	1000.86663480376
3.5	789.944239398110	857.661486512481	411.205630066449
4	352.274399796439	304.420102958499	163.301822393501
4.5	174.301212070274	103.849256419049	65.0764723307726
5	88.6862919275210	38.2353086841648	26.7168687722215
5.5	46.9957407094645	16.5839238624203	12.2582242456254
6	27.4690681125070	7.05809895122689	5.61527102638286
7	9.62840564153051	3.98164334748988	2.81680670660785
7.5	6.54264902323988	2.29437827316560	1.69868342196653

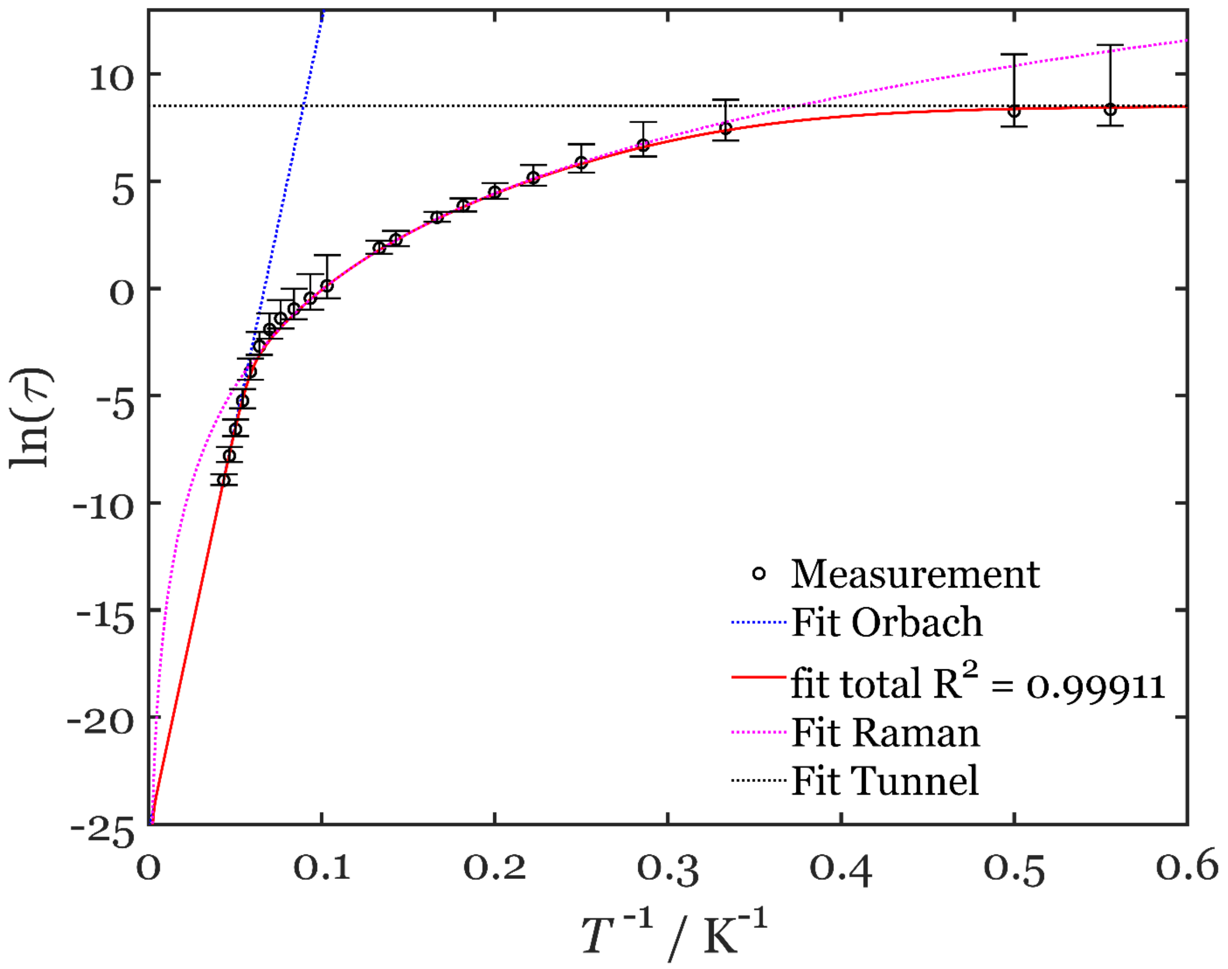

Figure S76: Arrhenius plot at 1000 Oe of **5-Er** using the data and uncertainties/ error bars from Table S16 and Table S18. The fit function is shown in equation 1 and the parameters in Table 2. In addition to the complete function (red line), the contributions from the Orbach process (blue dotted line), Raman process (pink dotted line) and the QTM (black dotted line) are shown.

ZFC and FC measurements

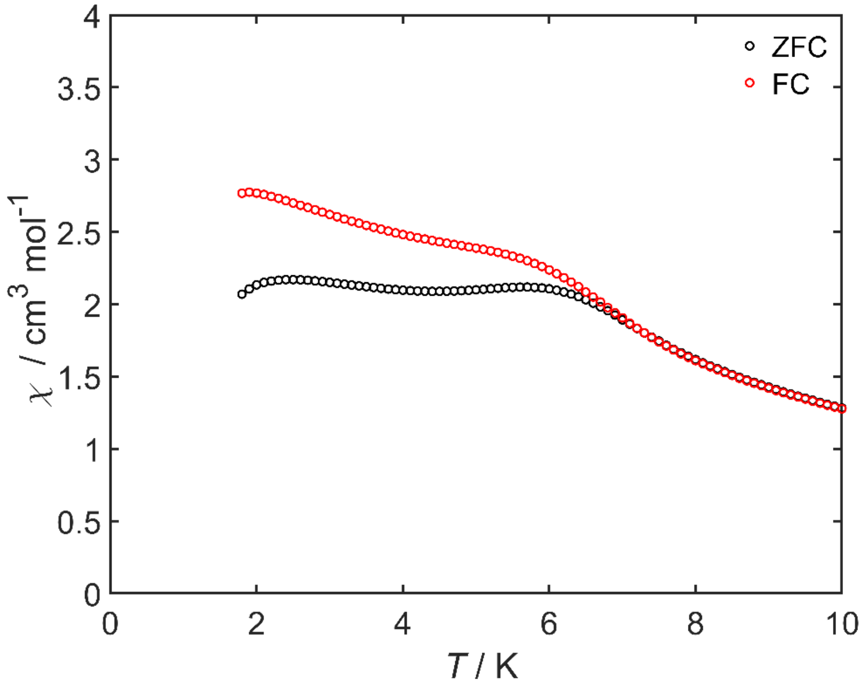

Figure S77: ZFC and FC measurements of **3-Er.** The sample was heated with a rate of 0.5 K/min and magnetization was measured continuously

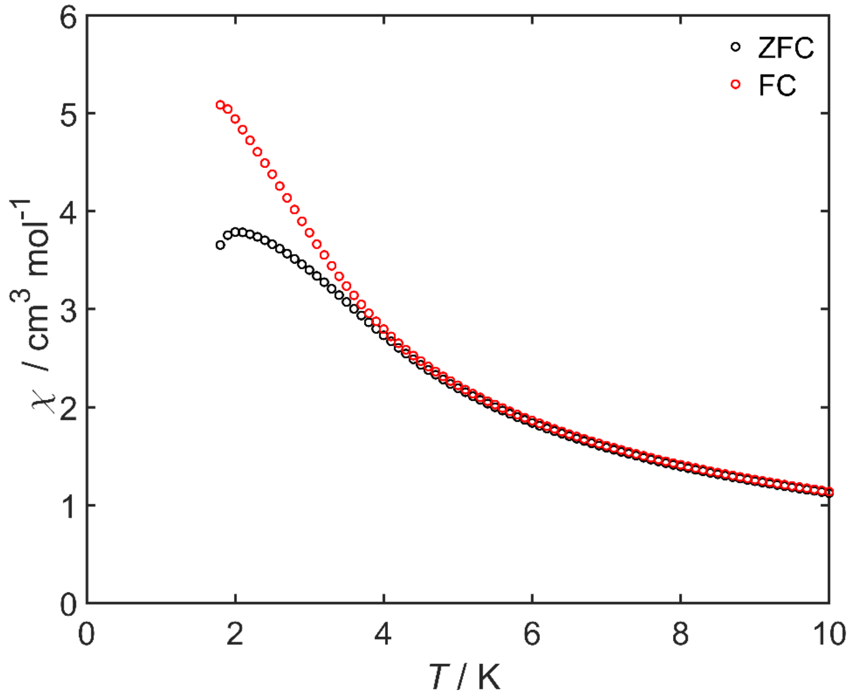

Figure S78: ZFC and FC measurements of **4-Er.** The sample was heated with a rate of 0.5 K/min and magnetization was measured continuously.

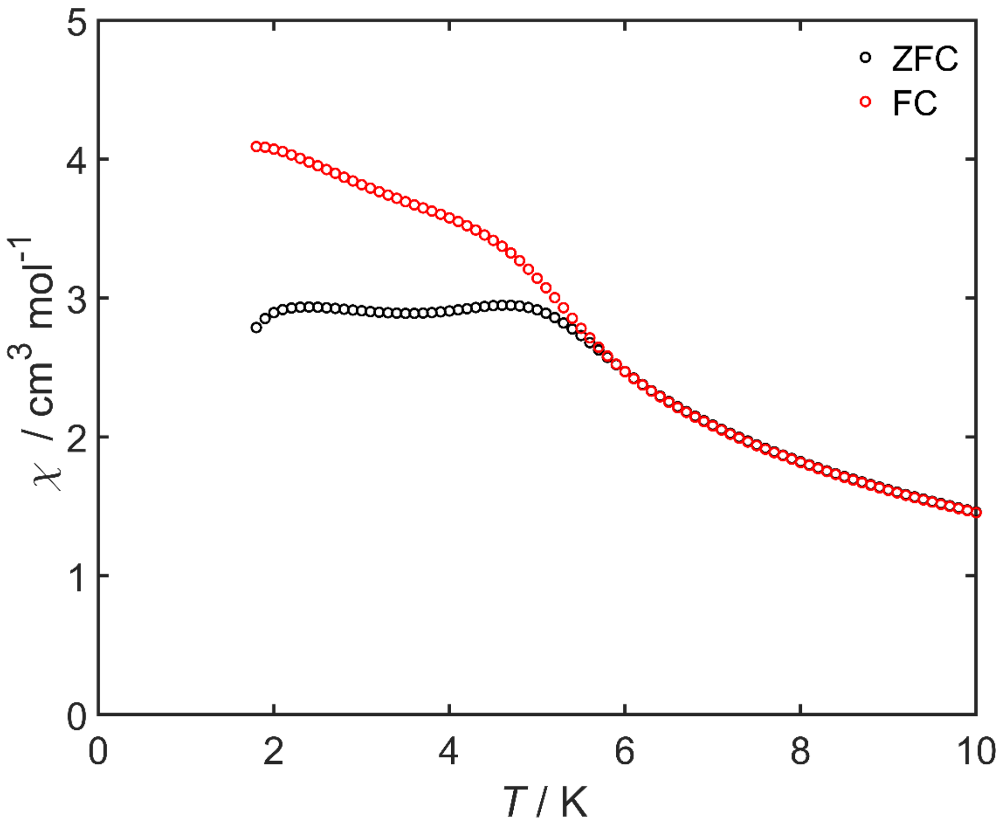

Figure S79: ZFC and FC measurements of **5-Er.** The sample was heated with a rate of 0.5 K/min and magnetization was measured continuously.

CASSCF Calculations

CASSCF-SO calculations were performed for **3-Er**, **4-Er** and **5-Er** employing the experimental structure of the complexes as experimentally determined. The CASSCF-SO electronic structure calculation of compounds were carried out employing OpenMolcas.[33-36] The basis sets from ANO-RCC library were employed[35,37,38] with VTZP quality for Er, VDZP quality for all atoms directly bound to the Er^{III} ion, while VDZ quality for all remaining atoms was employed, using the second-order DKH transformation.[39] The molecular orbitals (MOs) were optimized in state-averaged CASSCF calculations with an active space defined by the eleven 4f electrons in the seven 4f orbitals of Er^{III}. Three calculations were performed independently for each possible spin state, where 35 roots were included for S = 3/2 and 112 roots were included for S = 1/2 (RASSCF routine). The wavefunctions obtained from these CASSCF calculations were subsequently mixed by spin-orbit coupling, where all 35 S = 3/2 states and the 112 of the S = 1/2 states were included (RASSI routine[40]). The resulting spin-orbit wavefunctions were decomposed into their CF wavefunctions on the $^6I_{15/2}$ basis, employing the SINGLE_ANISO routine.[41,42]

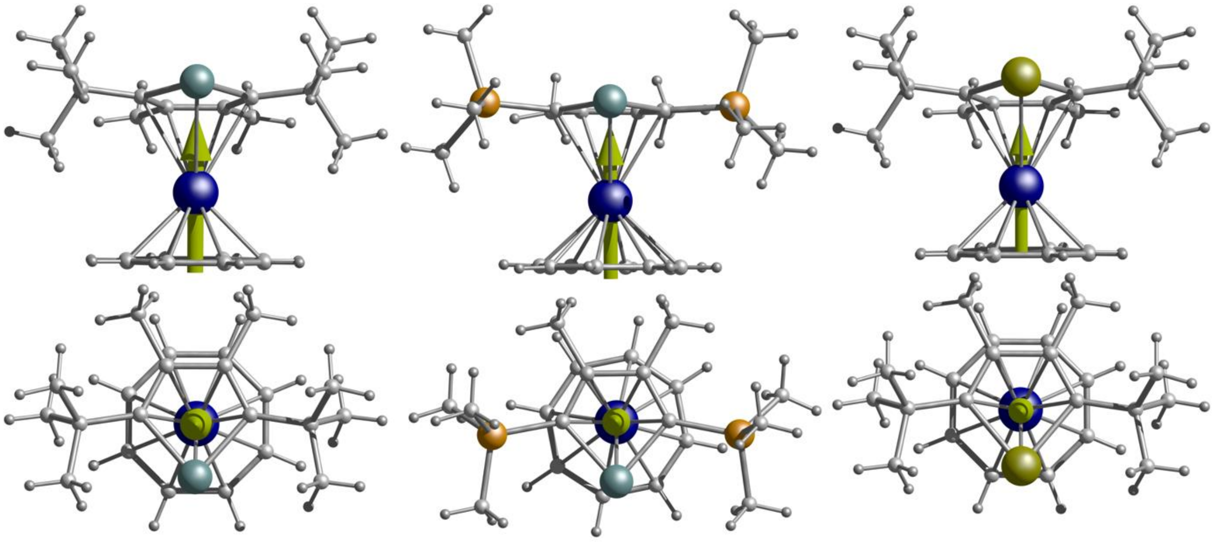

Figure S80: Magnetic axes for **3-Er**, **4-Er** and **5-Er** as obtained from CASSCF calculations (green arrow). Color code: Er, blue; C, light grey, H, grey; Sb, turquoise; Bi, green; Si, orange.

Table S19: Computed energy levels (the ground state is set at zero) composition of the g-tensor (g_x, g_y, g_z) and the main components (>10%) of the wavefunction for each m_J state of the ground-state multiplet $^4I_{15/2}$ for **3-Er**, at the CASSCF level.

Energy (cm^{-1})	g_x	g_y	g_z	Wavefunction
0	0.0005	0.0006	17.9120	99.5% |±15/2>
149.394	0.0087	0.0132	15.4372	83.4% |±13/2>+12.5%|±11/2>
199.270	9.0679	8.4180	2.3559	11.9%|±5/2>+44.5%|±3/2>+16.4% |±1/2>
207.556	1.3379	1.9453	8.6759	14.0%|±11/2>+14.6%|±9/2>+16.4%|±5/2>+24.8% |±1/2>
236.633	0.1721	2.2556	11.1809	16.1%|±11/2>+23.7%|±9/2>+26.4%|±3/2>
251.431	0.2918	2.8962	9.9458	10.1%|±13/2>+15.2%|±11/2>+28.3%|±7/2>+39.9%|±5/2>
264.407	0.3204	1.3603	14.0486	46.0%|±7/2>+28.5%|±5/2>+18.1%|±3/2>+19.0%|±1/2>
294.656	0.0088	0.0447	15.9044	31.9%|±11/2>+55.0%|±9/2>+10.9%|±7/2>

Table S20: Computed energy levels (the ground state is set at zero) composition of the g-tensor (g_x, g_y, g_z) and the main components (>10%) of the wavefunction for each m_J state of the ground-state multiplet $^4I_{15/2}$ for 4-Er, at the CASSCF level.

Energy (cm^{-1})	g_x	g_y	g_z	Wavefunction
0	0.0010	0.0011	17.9188	99.5% |±15/2>
169.932	0.0999	0.1597	15.0354	94.2% |±13/2>
220.830	3.1778	3.7449	9.6289	29.2%|±11/2>+32.5% |±3/2>+33.2% |±1/2>
230.997	1.9805	4.4153	10.9149	31.1% |±3/2>+53.0% |±1/2>
240.558	1.3677	2.47501	9.6146	10.1% |±7/2>+72.9% |±5/2>
247.332	1.4868	4.3887	7.8321	31.9%|±11/2>+28.1%|±9/2>+22.2% |±3/2>
252.613	8.2099	4.6296	0.9989	11.9%|±11/2>+13.3%|±9/2>+13.6% |±5/2>
258.624	1.0442	3.46289	11.0993	10.9%|±11/2>+68.1%|±9/2>

Table S21: Computed energy levels (the ground state is set at zero) composition of the g-tensor (g_x, g_y, g_z) and the main components (>10%) of the wavefunction for each m_J state of the ground-state multiplet $^4I_{15/2}$ for 5-Er, at the CASSCF level.

Energy (cm^{-1})	g_x	g_y	g_z	Wavefunction
0	0.0017	0.0020	17.8927	99.3% |±15/2>
145.034	0.0245	0.0262	15.2438	86.2% |±13/2>
204.306	0.5029	3.3052	13.0125	24.2%|±3/2>+58.6% |±1/2>
209.710	3.9446	5.49534	7.63582	18.5%|±11/2>+15.2%|±5/2>+30.4% |±3/2>+23.8% |±1/2>
246.275	1.0056	2.38983	11.3179	22.6%|±11/2>+21.0%|±9/2>+31.6% |±3/2>+15.1% |±1/2>
261.729	1.0879	4.28344	8.87521	16.1%|±11/2>+21.4%|±7/2>+48.9% |±5/2>
275.528	1.3800	2.44738	12.8370	53.0%|±7/2>+23.2%|±5/2>+10.6% |±3/2>
303.243	0.0521	0.13338	15.6339	57.8%|±9/2>+17.4%|±7/2>

Table S22: Crystal field Hamiltonian is given as $\hat{\boldsymbol{H}}_{\boldsymbol{CF}} = \sum_{k,q} \boldsymbol{B}_k^q \boldsymbol{O}_k^q$ and the extended Stevens operator coefficients $\boldsymbol{B}_k^q$ are extracted from CASSCF calculations for all Er-containing complexes.

			B_k^q	
k	*q*	**3-Er**	**4-Er**	**5-Er**
2	-2	-1.02852E-4	0.00697	6.25563E-4
2	-1	-1.29528E-4	0.03889	-0.02348
2	0	-1.07236	-1.11229	-1.12829
2	1	0.86356	-0.07699	0.79786
2	2	0.18879	0.10355	0.1802
4	-4	5.16316E-6	-0.00111	-4.26971E-5
4	-3	1.56639E-6	-0.00422	2.05618E-4
4	-2	9.35473E-7	2.64002E-4	-6.20585E-5
4	-1	-3.20518E-6	6.93227E-4	3.69345E-5
4	0	-0.00513	-0.00461	-0.00534
4	1	0.00517	4.98602E-4	0.0045
4	2	-6.41971E-4	0.00228	-6.57631E-4
4	3	-0.00746	8.51729E-4	-0.00927
4	4	-0.00289	-0.00278	-0.00353
6	-6	-2.57619E-8	-8.00234E-6	-1.37408E-6
6	-5	-4.03959E-7	-1.81555E-4	1.24983E-5
6	-4	1.8992E-7	-2.43141E-5	1.0036E-6
6	-3	1.18903E-7	-5.83387E-5	3.42153E-6
6	-2	1.9413E-8	5.59644E-6	1.60235E-6
6	-1	6.69419E-8	-2.02436E-5	-3.18593E-6
6	0	-2.78196E-6	-1.01027E-5	2.60859E-6
6	1	-1.8973E-4	4.25934E-7	-1.69684E-4
6	2	-4.1228E-5	2.23167E-5	-5.3244E-5
6	3	-1.23795E-4	1.09147E-5	-1.59654E-4
6	4	-9.05516E-5	-7.02157E-5	-1.12646E-4
6	5	4.14349E-5	1.07547E-4	1.68785E-4
6	6	1.74237E-5	-1.8212E-5	3.30924E-5

Table S23: CASSCF calculated average transition magnetic moment matrix elements in units μ² for **3-Er**.

Multiplet	Matrix Elements	Average	Multiplet	Matrix Elements	Average
	+I* to *-I			***I* to *I*+3**	
1	⟨+1.1|−1.1⟩	1.87025E-4	1	⟨+1.1|+4.1⟩	0.18843
2	⟨+2.1|−2.1⟩	0.00372	1	⟨+1.1|−4.1⟩	0.05636
3	⟨+3.1|−3.1⟩	2.48903	2	⟨+2.1|+5.1⟩	0.9558
4	⟨+4.1|−4.1⟩	0.72852	2	⟨+2.1|−5.1⟩	0.11096
5	⟨+5.1|−5.1⟩	0.65537	3	⟨+3.1|+6.1⟩	1.66191
6	⟨+6.1|−6.1⟩	0.54486	3	⟨+3.1|−6.1⟩	0.31185
7	⟨+7.1|−7.1⟩	0.56259	4	⟨+4.1|+7.1⟩	0.81061
8	⟨+8.1|−8.1⟩	0.00952	4	⟨+4.1|−7.1⟩	0.15532
	***I* to *I*+1**		5	⟨+5.1|+8.1⟩	0.59888
1	⟨+1.1|+2.1⟩	1.45649	5	⟨+5.1|−8.1⟩	0.30763
1	⟨+1.1|−2.1⟩	9.36949E-4		***I* to *I*+4**	
2	⟨+2.1|+3.1⟩	1.03803	1	⟨+1.1|+5.1⟩	0.22068
2	⟨+2.1|−3.1⟩	0.34377	1	⟨+1.1|−5.1⟩	0.0493
3	⟨+3.1|+4.1⟩	2.40658	2	⟨+2.1|+6.1⟩	0.87641
3	⟨+3.1|−4.1⟩	1.6068	2	⟨+2.1|−6.1⟩	0.10402
4	⟨+4.1|+5.1⟩	1.88523	3	⟨+3.1|+7.1⟩	0.47203
4	⟨+4.1|−5.1⟩	0.75969	3	⟨+3.1|−7.1⟩	0.27847
5	⟨+5.1|+6.1⟩	1.22912	4	⟨+4.1|+8.1⟩	0.40223
5	⟨+5.1|−6.1⟩	1.13124	4	⟨+4.1|−8.1⟩	0.17982
6	⟨+6.1|+7.1⟩	1.68059		***I* to *I*+5**	
6	⟨+6.1|−7.1⟩	1.03263	1	⟨+1.1|+6.1⟩	0.37151
7	⟨+7.1|+8.1⟩	0.80464	1	⟨+1.1|−6.1⟩	0.02533
7	⟨+7.1|−8.1⟩	0.51495	2	⟨+2.1|+7.1⟩	0.27483
	***I* to *I*+2**		2	⟨+2.1|−7.1⟩	0.1695
1	⟨+1.1|+3.1⟩	0.23007	3	⟨+3.1|+8.1⟩	0.52626
1	⟨+1.1|−3.1⟩	0.02239	3	⟨+3.1|−8.1⟩	0.1486
2	⟨+2.1|+4.1⟩	1.4448		***I* to *I*+6**	
2	⟨+2.1|−4.1⟩	0.13272	1	⟨+1.1|+7.1⟩	0.10571
3	⟨+3.1|+5.1⟩	1.19297	1	⟨+1.1|−7.1⟩	0.06751
3	⟨+3.1|−5.1⟩	0.34042	2	⟨+2.1|+8.1⟩	0.51081
4	⟨+4.1|+6.1⟩	1.49875	2	⟨+2.1|−8.1⟩	0.0038
4	⟨+4.1|−6.1⟩	0.3514		***I* to *I*+7**	
5	⟨+5.1|+7.1⟩	1.54857	1	⟨+1.1|−8.1⟩	0.32551
5	⟨+5.1|−7.1⟩	0.73747	1	⟨+1.1|+8.1⟩	6.73774E-4
6	⟨+6.1|+8.1⟩	1.84023			
6	⟨+6.1|−8.1⟩	0.10307			

Table S24: CASSCF calculated average transition magnetic moment matrix elements in units μ² for **4-Er**.

Multiplet	Matrix Elements	Average	Multiplet	Matrix Elements	Average
	**+*I* to -*I***			***I* to *I*+3**	
1	⟨+1.1|−1.1⟩	3.56275E-4	1	⟨+1.1|+4.1⟩	0.06146
2	⟨+2.1|−2.1⟩	0.04333	1	⟨+1.1|−4.1⟩	0.02466
3	⟨+3.1|−3.1⟩	1.63954	2	⟨+2.1|+5.1⟩	0.92383
4	⟨+4.1|−4.1⟩	1.98278	2	⟨+2.1|−5.1⟩	0.21696
5	⟨+5.1|−5.1⟩	0.79111	3	⟨+3.1|+6.1⟩	1.14324
6	⟨+6.1|−6.1⟩	1.07614	3	⟨+3.1|−6.1⟩	0.55959
7	⟨+7.1|−7.1⟩	1.03383	4	⟨+4.1|+7.1⟩	1.03466
8	⟨+8.1|−8.1⟩	0.92276	4	⟨+4.1|−7.1⟩	0.73416
	***I* to *I*+1**		5	⟨+5.1|+8.1⟩	0.99948
1	⟨+1.1|+2.1⟩	1.54866	5	⟨+5.1|−8.1⟩	0.52016
1	⟨+1.1|−2.1⟩	0.00184		***I* to *I*+4**	
2	⟨+2.1|+3.1⟩	1.6886	1	⟨+1.1|+5.1⟩	0.19743
2	⟨+2.1|−3.1⟩	0.20907	1	⟨+1.1|−5.1⟩	0.0319
3	⟨+3.1|+4.1⟩	2.11649	2	⟨+2.1|+6.1⟩	1.03383
3	⟨+3.1|−4.1⟩	1.32448	2	⟨+2.1|−6.1⟩	0.21226
4	⟨+4.1|+5.1⟩	1.36598	3	⟨+3.1|+7.1⟩	0.44011
4	⟨+4.1|−5.1⟩	0.57676	3	⟨+3.1|−7.1⟩	0.32525
5	⟨+5.1|+6.1⟩	1.45372	4	⟨+4.1|+8.1⟩	0.69649
5	⟨+5.1|−6.1⟩	0.70604	4	⟨+4.1|−8.1⟩	0.68227
6	⟨+6.1|+7.1⟩	1.59516		***I* to *I*+5**	
6	⟨+6.1|−7.1⟩	0.80813	1	⟨+1.1|+6.1⟩	0.14333
7	⟨+7.1|+8.1⟩	2.31103	1	⟨+1.1|−6.1⟩	0.03761
7	⟨+7.1|−8.1⟩	1.05601	2	⟨+2.1|+7.1⟩	0.42472
	***I* to *I*+2**		2	⟨+2.1|−7.1⟩	0.24397
1	⟨+1.1|+3.1⟩	0.05705	3	⟨+3.1|+8.1⟩	0.69275
1	⟨+1.1|−3.1⟩	0.06408	3	⟨+3.1|−8.1⟩	0.24219
2	⟨+2.1|+4.1⟩	0.74685		***I* to *I*+6**	
2	⟨+2.1|−4.1⟩	0.1052	1	⟨+1.1|+7.1⟩	0.11002
3	⟨+3.1|+5.1⟩	1.85456	1	⟨+1.1|−7.1⟩	0.04959
3	⟨+3.1|−5.1⟩	0.70942	2	⟨+2.1|+8.1⟩	0.41745
4	⟨+4.1|+6.1⟩	1.40315	2	⟨+2.1|−8.1⟩	0.21875
4	⟨+4.1|−6.1⟩	0.27564		***I* to *I*+7**	
5	⟨+5.1|+7.1⟩	2.0052	1	⟨+1.1|−8.1⟩	0.09404
5	⟨+5.1|−7.1⟩	1.07291	1	⟨+1.1|+8.1⟩	0.02758
6	⟨+6.1|+8.1⟩	1.81793			
6	⟨+6.1|−8.1⟩	1.03072			

Table S25: CASSCF calculated average transition magnetic moment matrix elements in units μ² for **5-Er**.

Multiplet	Matrix Elements	Average	Multiplet	Matrix Elements	Average
	+I* to *-I			***I* to *I*+3**	
1	⟨+1.1|−1.1⟩	6.15737E-4	1	⟨+1.1|+4.1⟩	0.24345
2	⟨+2.1|−2.1⟩	0.00856	1	⟨+1.1|−4.1⟩	0.05744
3	⟨+3.1|−3.1⟩	2.6569	2	⟨+2.1|+5.1⟩	1.00536
4	⟨+4.1|−4.1⟩	1.92694	2	⟨+2.1|−5.1⟩	0.15238
5	⟨+5.1|−5.1⟩	0.85689	3	⟨+3.1|+6.1⟩	1.33321
6	⟨+6.1|−6.1⟩	0.9657	3	⟨+3.1|−6.1⟩	0.39414
7	⟨+7.1|−7.1⟩	1.08191	4	⟨+4.1|+7.1⟩	0.84486
8	⟨+8.1|−8.1⟩	0.03492	4	⟨+4.1|−7.1⟩	0.57046
	***I* to *I*+1**		5	⟨+5.1|+8.1⟩	0.60733
1	⟨+1.1|+2.1⟩	1.49047	5	⟨+5.1|−8.1⟩	0.52741
1	⟨+1.1|−2.1⟩	0.00269		***I* to *I*+4**	
2	⟨+2.1|+3.1⟩	0.83545	1	⟨+1.1|+5.1⟩	0.15205
2	⟨+2.1|−3.1⟩	0.69278	1	⟨+1.1|−5.1⟩	0.06032
3	⟨+3.1|+4.1⟩	2.28105	2	⟨+2.1|+6.1⟩	0.83104
3	⟨+3.1|−4.1⟩	1.00244	2	⟨+2.1|−6.1⟩	0.14625
4	⟨+4.1|+5.1⟩	1.56435	3	⟨+3.1|+7.1⟩	0.3327
4	⟨+4.1|−5.1⟩	1.07351	3	⟨+3.1|−7.1⟩	0.56918
5	⟨+5.1|+6.1⟩	1.23841	4	⟨+4.1|+8.1⟩	0.61596
5	⟨+5.1|−6.1⟩	1.43877	4	⟨+4.1|−8.1⟩	0.22341
6	⟨+6.1|+7.1⟩	1.56084		***I* to *I*+5**	
6	⟨+6.1|−7.1⟩	1.53623	1	⟨+1.1|+6.1⟩	0.28957
7	⟨+7.1|+8.1⟩	0.96816	1	⟨+1.1|−6.1⟩	0.042
7	⟨+7.1|−8.1⟩	0.87596	2	⟨+2.1|+7.1⟩	0.30185
	***I* to *I*+2**		2	⟨+2.1|−7.1⟩	0.23323
1	⟨+1.1|+3.1⟩	0.16947	3	⟨+3.1|+8.1⟩	0.40398
1	⟨+1.1|−3.1⟩	0.02307	3	⟨+3.1|−8.1⟩	0.18167
2	⟨+2.1|+4.1⟩	1.50202		***I* to *I*+6**	
2	⟨+2.1|−4.1⟩	0.26544	1	⟨+1.1|+7.1⟩	0.12933
3	⟨+3.1|+5.1⟩	1.18561	1	⟨+1.1|−7.1⟩	0.09835
3	⟨+3.1|−5.1⟩	0.89491	2	⟨+2.1|+8.1⟩	0.46496
4	⟨+4.1|+6.1⟩	1.7382	2	⟨+2.1|−8.1⟩	0.0181
4	⟨+4.1|−6.1⟩	0.19355		***I* to *I*+7**	
5	⟨+5.1|+7.1⟩	1.3132	1	⟨+1.1|−8.1⟩	0.28998
5	⟨+5.1|−7.1⟩	0.73896	1	⟨+1.1|+8.1⟩	0.00342
6	⟨+6.1|+8.1⟩	1.67828			
6	⟨+6.1|−8.1⟩	0.16825			

Supplementary References

1 Münzfeld, L. *et al.* Synthesis, structures and magnetic properties of [(η^9-C_9H_9)Ln(η^8-C_8H_8)] super sandwich complexes. *Nat. Commun.* **10**, 3135 (2019).

2 Turcitu, D., Nief, F. & Ricard, L. Structure and Reactivity of Homoleptic Samarium(II) and Thulium(II) Phospholyl Complexes. *Chem. Eur. J.* **9**, 4916-4923 (2003).

3 Yamaguchi, S., Jin, R.-Z. & Tamao, K. Modification of the electronic structure of silole by the substituents on the ring silicon. *J. Organomet. Chem.* **559**, 73-80 (1998).

4 Chalmers, B. A., Bühl, M., Athukorala Arachchige, K. S., Slawin, A. M. Z. & Kilian, P. Structural, Spectroscopic and Computational Examination of the Dative Interaction in Constrained Phosphine–Stibines and Phosphine–Stiboranes. *Chem. Eur. J.* **21**, 7520-7531 (2015).

5 Hirayama, T. *et al.* Bismuth-rhodamine: a new red light-excitable photosensitizer. *Dalton. Trans.* **46**, 15991-15995 (2017).

6 Münzfeld, L. *et al.* Molecular Lanthanide Switches for Magnetism and Photoluminescence. *Angew. Chem. Int. Ed.* **62**, e202218107 (2023).

7 Schwarz, N. *Dissertation, Karlsruher Institut für Technologie (KIT)*: *Synthese von Seltenerd-Sandwichkomplexen mit Gruppe 15-Heterocyclopentadienyl-Liganden und Redoxtransmetallierung von Lanthanoiden und Erdalkalimetallen*; Cuvillier Verlag Göttingen, **2024**.

8 Dolomanov, O. V., Bourhis, L. J., Gildea, R. J., Howard, J. A. K. & Puschmann, H. OLEX2: a complete structure solution, refinement and analysis program. *J. Appl. Crystallogr.* **42**, 339-341 (2009).

9 Sheldrick, G. A short history of SHELX. *Acta Crystallogr. A* **64**, 112-122 (2008).

10 Sheldrick, G. SHELXT - Integrated space-group and crystal-structure determination. *Acta Crystallogr. A* **71**, 3-8 (2015).

11 Sheldrick, G. Crystal structure refinement with SHELXL. *Acta Crystallogr. C* **71**, 3-8 (2015).

12 TURBOMOLE V7.7 2022, a development of University of Karlsruhe and Forschungszentrum Karlsruhe GmbH, 1989–2007, TURBOMOLE GmbH, since 2007; available from https://www.turbomole.org.

13 Weigend, F. & Ahlrichs, R. Balanced basis sets of split valence, triple zeta valence and quadruple zeta valence quality for H to Rn: Design and assessment of accuracy. *PCCP* **7**, 3297-3305 (2005).

14 Gulde, R., Pollak, P. & Weigend, F. Error-Balanced Segmented Contracted Basis Sets of Double-ζ to Quadruple-ζ Valence Quality for the Lanthanides. *J. Chem. Theory Comput.* **8**, 4062-4068 (2012).

15 Perdew, J. P., Burke, K. & Ernzerhof, M. Generalized Gradient Approximation Made Simple. *Phys. Rev. Lett.* **77**, 3865-3868 (1996).

16 Adamo, C. & Barone, V. Toward reliable density functional methods without adjustable parameters: The PBE0 model. *J. Chem. Phys.* **110**, 6158-6170 (1999).

17 Treutler, O. & Ahlrichs, R. Efficient molecular numerical integration schemes. *J. Chem. Phys.* **102**, 346-354 (1995).

18 Metz, B., Stoll, H. & Dolg, M. Small-core multiconfiguration-Dirac–Hartree–Fock-adjusted pseudopotentials for post-d main group elements: Application to PbH and PbO. *J. Chem. Phys.* **113**, 2563-2569 (2000).

19 Dolg, M., Stoll, H. & Preuss, H. Energy-adjusted ab initio pseudopotentials for the rare earth elements. *J. Chem. Phys.* **90**, 1730-1734 (1989).

20 Andrae, D., Häußermann, U., Dolg, M., Stoll, H. & Preuß, H. Energy-adjusted ab initio pseudopotentials for the second and third row transition elements. *Theor. Chim. Acta* **77**, 123-141 (1990).

21 Caldeweyher, E., Bannwarth, C. & Grimme, S. Extension of the D3 dispersion coefficient model. *J. Chem. Phys.* **147** (2017).

22 Reiter, K., Mack, F. & Weigend, F. Calculation of Magnetic Shielding Constants with meta-GGA Functionals Employing the Multipole-Accelerated Resolution of the Identity: Implementation and Assessment of Accuracy and Efficiency. *J. Chem. Theory Comput.* **14**, 191-197 (2018).

23 Sierka, M., Hogekamp, A. & Ahlrichs, R. Fast evaluation of the Coulomb potential for electron densities using multipole accelerated resolution of identity approximation. *J. Chem. Phys.* **118**, 9136-9148 (2003).

24 Weigend, F. Accurate Coulomb-fitting basis sets for H to Rn. *PCCP* **8**, 1057-1065 (2006).

25 Fliegl, H., Taubert, S., Lehtonen, O. & Sundholm, D. The gauge including magnetically induced current method. *PCCP* **13**, 20500-20518 (2011).

26 Jusélius, J., Sundholm, D. & Gauss, J. Calculation of current densities using gauge-including atomic orbitals. *J. Chem. Phys.* **121**, 3952-3963 (2004).

27 GIMIC Version 2.1.4, available via https://github.com/qmcurrents/gimic.

28 Gillhuber, S., Franzke, Y. J. & Weigend, F. Paramagnetic NMR Shielding Tensors and Ring Currents: Efficient Implementation and Application to Heavy Element Compounds. *J. Phys. Chem. A* **125**, 9707-9723 (2021).

29 Mulliken, R. S. Electronic Population Analysis on LCAO–MO Molecular Wave Functions. I. *J. Chem. Phys.* **23**, 1833-1840 (1955).

30 Blackmore, W. J. A. *et al.* Characterisation of magnetic relaxation on extremely long timescales. *PCCP* **25**, 16735-16744 (2023).

31 Gatteschi, D., Sessoli, R. & Villain, J. *Molecular Nanomagnets*. (Oxford University Press, 2006).

32 Zorn, R. Logarithmic moments of relaxation time distributions. *J. Chem. Phys.* **116**, 3204-3209 (2002).

33 Fdez. Galván, I. *et al.* OpenMolcas: From Source Code to Insight. *J. Chem. Theory Comput.* **15**, 5925-5964 (2019).

34 Aquilante, F. *et al.* Molcas 8: New capabilities for multiconfigurational quantum chemical calculations across the periodic table. *J. Comput. Chem.* **37**, 506-541 (2016).

35 Roos, B. O., Lindh, R., Malmqvist, P.-Å., Veryazov, V. & Widmark, P.-O. Main Group Atoms and Dimers Studied with a New Relativistic ANO Basis Set. *J. Phys. Chem. A* **108**, 2851-2858 (2004).

36 Roos, B. O., Lindh, R., Malmqvist, P.-Å., Veryazov, V. & Widmark, P.-O. New Relativistic ANO Basis Sets for Transition Metal Atoms. *J. Phys. Chem. A* **109**, 6575-6579 (2005).

37 Roos, B. O. *et al.* New Relativistic Atomic Natural Orbital Basis Sets for Lanthanide Atoms with Applications to the Ce Diatom and LuF_3. *J. Phys. Chem. A* **112**, 11431-11435 (2008).

38 Widmark, P.-O., Malmqvist, P.-Å. & Roos, B. O. Density matrix averaged atomic natural orbital (ANO) basis sets for correlated molecular wave functions. *Theor. Chim. Acta* **77**, 291-306 (1990).

39 Peng, D. & Hirao, K. An arbitrary order Douglas–Kroll method with polynomial cost. *J. Chem. Phys.* **130** (2009).

40 Malmqvist, P. Å., Roos, B. O. & Schimmelpfennig, B. The restricted active space (RAS) state interaction approach with spin–orbit coupling. *Chem. Phys. Lett.* **357**, 230-240 (2002).

41 Chibotaru, L. F. & Ungur, L. Ab initio calculation of anisotropic magnetic properties of complexes. I. Unique definition of pseudospin Hamiltonians and their derivation. *J. Chem. Phys.* **137**, 064112 (2012).

42 Ungur, L. & Chibotaru, L. F. Ab Initio Crystal Field for Lanthanides. *Chem. Eur. J.* **23**, 3708-3718 (2017).
